# Predicted rat interactome database and gene set linkage analysis

**DOI:** 10.1093/database/baaa086

**Published:** 2020-11-20

**Authors:** Yu-Tian Tao, Xiao-Bao Ding, Jie Jin, Hai-Bo Zhang, Wen-Ping Guo, Li Ruan, Qiao-Lei Yang, Peng-Cheng Chen, Heng Yao, Xin Chen

**Affiliations:** Institute of Big data and Artificial Intelligence in Medicine, School of Electronics & Information Engineering, Taizhou University, 1139 Shifu Avenue, Taizhou, 318000, China; Institute of Big data and Artificial Intelligence in Medicine, School of Electronics & Information Engineering, Taizhou University, 1139 Shifu Avenue, Taizhou, 318000, China; Institute of Big data and Artificial Intelligence in Medicine, School of Electronics & Information Engineering, Taizhou University, 1139 Shifu Avenue, Taizhou, 318000, China; Institute of Big data and Artificial Intelligence in Medicine, School of Electronics & Information Engineering, Taizhou University, 1139 Shifu Avenue, Taizhou, 318000, China; Institute of Big data and Artificial Intelligence in Medicine, School of Electronics & Information Engineering, Taizhou University, 1139 Shifu Avenue, Taizhou, 318000, China; Institute of Big data and Artificial Intelligence in Medicine, School of Electronics & Information Engineering, Taizhou University, 1139 Shifu Avenue, Taizhou, 318000, China; Institute of Pharmaceutical Biotechnology, School of Medicine, Zhejiang University, 866 Yuhangtang Road, Hangzhou, 310058, China; Institute of Pharmaceutical Biotechnology, School of Medicine, Zhejiang University, 866 Yuhangtang Road, Hangzhou, 310058, China; Institute of Pharmaceutical Biotechnology, School of Medicine, Zhejiang University, 866 Yuhangtang Road, Hangzhou, 310058, China; Institute of Big data and Artificial Intelligence in Medicine, School of Electronics & Information Engineering, Taizhou University, 1139 Shifu Avenue, Taizhou, 318000, China; Institute of Pharmaceutical Biotechnology, School of Medicine, Zhejiang University, 866 Yuhangtang Road, Hangzhou, 310058, China; Joint Institute for Genetics and Genome Medicine between Zhejiang University and University of Toronto, Zhejiang University, 866 Yuhangtang Road, Hangzhou, 310058, China

## Abstract

*Rattus norvegicus*, or the rat, has been widely used as animal models for a diversity of human diseases in the last 150 years. The rat, as a disease model, has the advantage of relatively large body size and highly similar physiology to humans. In drug discovery, rat models are routinely used in drug efficacy and toxicity assessments. To facilitate molecular pharmacology studies in rats, we present the predicted rat interactome database (PRID), which is a database of high-quality predicted functional gene interactions with balanced sensitivity and specificity. PRID integrates functional gene association data from 10 public databases and infers 305 939 putative functional associations, which are expected to include 13.02% of all rat protein interactions, and 52.59% of these function associations may represent protein interactions. This set of functional interactions may not only facilitate hypothesis formulation in molecular mechanism studies, but also serve as a reference interactome for users to perform gene set linkage analysis (GSLA), which is a web-based tool to infer the potential functional impacts of a set of changed genes observed in transcriptomics analyses. In a case study, we show that GSLA based on PRID may provide more precise and informative annotations for investigators to understand the physiological mechanisms underlying a phenotype and lead investigators to testable hypotheses for further studies. Widely used functional annotation tools such as Gene Ontology (GO) analysis, and Database for Annotation, Visualization and Integrated Discovery (DAVID) did not provide similar insights.

**Database URL**: http://rat.biomedtzc.cn

## Introduction

The rat was the first mammalian species domesticated in scientific research in the mid-1800s and soon became the most widely used biomedical model for >150 years (1). As a model for human diseases, rats are more advanced to mice and other organisms in many fields, such as neurobiology, cardiobiology, physiology and toxicology (2, 3). The large body size and more human-like physiology have made rats a good choice for biological mechanisms and drug discovery in human disease studies (4).

Over the last few years, the development of omics approaches in rats has provided novel insights into the molecular mechanisms of human diseases and has promoted the development of successful precision medicine (5, 6). Compared with the reductionist approaches in traditional technology, omics technology can comprehensively analyze the whole process in the biology system, which can provide information qualitatively and quantitatively on diseases, toxicities and therapies (7). However, the considerable and complex omics data also create unprecedented challenges in interpreting the underlying design logic of physiological processes from molecular-level descriptions.

To address these challenges, the existing approaches mostly rely on enrichment analysis to derive high-level biological sense from the omics data. These approaches evaluate whether the observed set of changed genes (SCG) between two physiological statuses are enriched or clustered in a typical biological process. To date, some enrichment-based gene set annotation tools have been developed and widely used, including gene ontology (8), Kyoto Encyclopedia of Genes and Genomes (KEGG) (9) and DAVID (10). In many cases, this strategy can summarize the observed SCG into the established biological concepts. However, in practice, these enrichment-based approaches frequently report no annotation term or report conceptually very general terms (such as GO:0019538, protein metabolic process) because no established annotation terms can be found to explain why such changes occur. Such results provide little value to elucidate the molecular mechanisms of the observed SCG to formulate hypotheses and design further experiments. However, when no established concepts accurately describe the observed SCG, we may still use established annotation terms to interpret the functional impacts of the expression changed gene sets. For instance, observed SCG may lead collectively to GO:0097411 (hypoxia-inducible factor [HIF]-1 alpha signaling pathway), even when the SCGs are not enriched in these terms (see ‘Discussion’ for details). Previously, we developed the gene set linkage analysis (GSLA) tool to interpret the potential functional impacts of observed SCG even though no established biological concepts are available to define these changes. GSLA evaluates whether the observed SCG has strong functional associations with the other gene sets representing established biological processes. If genes in SCG are densely associated with genes in a biological process, this SCG is expected to interfere with this biological function. GSLA has been successfully used in human and Arabidopsis transcriptome analyses (11, 12). The success of GSLA in these two species relies critically on the high-quality interactomes that were specially developed for GSLA in these species (11, 13). In this study, we adapted and applied the GSLA tool to the high-quality rat interactome predicted rat interactome database (PRID), to extend its capability for interpretation of the potential functional impacts of rat SCG.

In this study, we present the high-quality functional association gene network called the PRID for rats and its associated GSLA web tool in terms of their functional impacts at the biological process level. PRID integrates 6 types of evidence from 10 public databases with the date before 2018 to infer the functional associations between genes. The inference accuracy of PRID is evaluated using experimentally confirmed protein–protein interactions that were recently reported with the date after 2018. The current version of PRID includes 305 939 gene associations, which include 302 693 predicted functional associations and 3246 experimentally reported interactions. These 302 693 functional associations are expected to cover ∼13.02% protein–protein interactions of rats. Approximately 52.59% of these functional associations are expected to represent protein–protein interactions. We provide a web interface for PRID so that users can search the functional associations of their interested genes. We also provide the web interface for the PRID-based GSLA tool for users to interpret the collective functional impact of multiple simultaneously changed genes. Finally, a case study is presented to illustrate the use of PRID/GSLA.

## Materials and methods

### Data integration

To build the interaction prediction model, we selected six types of evidence for the suggestion of functional associations between genes by support vector machine (SVM) algorithm. Six types of indirect evidence were collected from 7 public databases before the year 2018, including 17 353 expression profiles (Coxpresdb), 434 836 gene annotations (GOC), 55 462 domain interactions (IDDI and Pfam), 15 922 subcellular gene localizations (Compartments), 20 120 phylogenetic profiles (DIOPT) and 14 807 homologs interaction in other species (Inparanoid).

**Figure 1.**
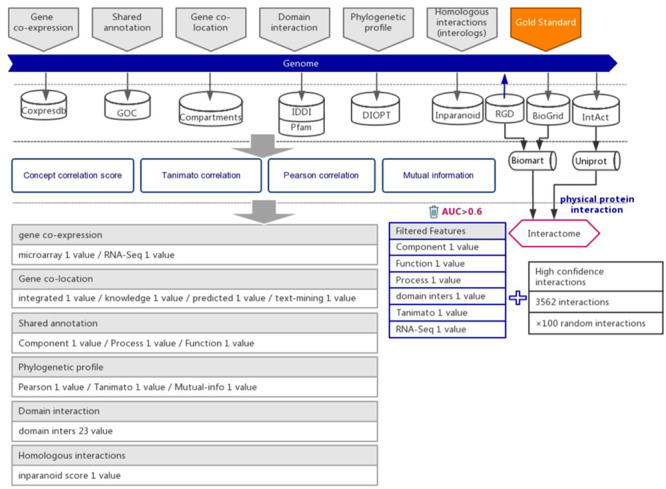
The workflow diagram for the influence of functional interactions between rat genes. The training dataset consisted of high-quality experimental confirmed protein interactions from three databases that were used as positive examples. Random gene pairs that did not overlap with positive examples were used as negative examples. The positive to negative ratio was 1:100. Six types of functional association evidence were collected from 10 databases. Six high-quality feature values were selected from 36 feature values that characterize the functional association evidence with different mathematical representations.

Protein–protein interactions are considered to be one type of strong functional association. Here, we retrieved and integrated 6251 experimental reported protein–protein interactions of rats from three public databases, including the Rat Genome Database (RGD) (14), BioGrid (15) and IntAct (16) (Supplementary Table 1). To ensure that the protein–protein interactions are experimentally confirmed rather than predicted, the original data were further curated and retained the interactions with more than one independent study, at least one of which was reported in low-throughput experiments. Because protein–protein interactions were integrated from different databases, we used UniProt (17) and BioMart (18) software to convert diverse gene IDs to the uniform RGD ID (Figure 1). After filtration, 2693 highly reliable protein–protein interactions with uniform RGD ID served as positive examples to train SVM models. A recently study proposed a categorization of detection methods for PPIs (19). After filtration, the fraction of our gold-standard data that are detected by at least one binary method from one independent study was increased from 11.98% to 17.21%, which suggested that, from this perspective, our filtering method also increased the reliability of our gold-standard dataset.

### The computation and evaluation of feature values

From these 6 types of functional association evidence, 36 feature values were computed to describe the functional associations between genes, including 3 shared annotation features, 2 co-expression features, 4 co-localization features, 23 domain interaction features, 3 phylogenetic profile features and 1 homologous interaction feature (Supplementary Table 2) (11, 12). Their detailed methods of computation can be found at the PRID website under Help/Indirect Evidence. To evaluate the quality of these 36 features in functional association suggestions, we used the area under the curve (AUC) of the receiver operating characteristic (ROC) test. When the feature value was used to predict the protein interactions, it produced a series of sensitivities and specificities by different cut-offs. Therefore, we plotted the ROC curve by these sensitivities and specificities generated by different cut-offs (X-axis, 1-specificity; Y-axis, sensitivity). We used an AUC > 0.6 as the criterion for feature selection. After evaluation, 6 features with AUC higher than 0.6 were considered to be high enough quality for strong functional association inference (Supplementary Table 3 and Supplementary Figure 1). These six features were used in subsequent model training. These features are set to zero if data necessary for calculating these features were missing for a pair of genes.

### Evaluation of interactions in PRID

PRID was inferred from data that were released before 1 January 2018. We selected 11 810 genes from the GO database with new annotations added after 2018. These 11 810 genes have a total of 376 904 annotations, 113 105 of which were newly updated since 2018. These genes and their new annotations (4891) were used to evaluate the quality of the interaction networks. For each of the 11 810 target genes, we first found all first-degree neighbors of a target gene in an interactome to create a gene set. This gene set was analyzed by Protein ANalysis THrough Evolutionary Relationships (PANTHER) to find enriched annotation terms For each *P*-value cut-off reported in PANTHER, we counted (i) how many terms were predicted by PANTHER (N); (ii) how many PANTHER predicted terms are among the 376 904 known annotations or their more specific terms (X) and (iii) how many of the 113 105 new terms have themselves or their more specific terms predicted by PANTHER (Y). The precision and recall are calculated as follows:

$$\begin{equation*}{\rm{Precision }} = {\ }{{\rm{X}} \over {\rm{N}}}\,\,\,{\rm{ Recall}} = {{\rm{Y}} \over {113,105}}\end{equation*}$$

### Microarray data analysis

The microarray data GSE80339 was retrieved from the Gene Expression Omnibus (GEO) database (20). In the original article, the authors identified 838 downregulated transcripts and 786 upregulated transcripts in low oxygen conditions compared with the ambient condition. We reanalyzed the expression dataset using the online tool GEO2R with default parameters. We selected the top 250 upregulated genes for interpretation using DAVID, PANTHER and PDIR/GSLA. All upregulated genes have adjusted *P*-value < 0.05 (Supplementary Table 4).

## Results

### Data integration for the prediction of functional associations between rat genes

Six types of evidence were selected for the prediction of interactions between rat genes, each suggesting a specific type of functional association (21, 22). We collected the indirect evidence from seven public databases, including Coxpresdb (23), Gene Ontology Consortium (GOC) (24), Compartments (25), IDDI (26), Pfam (27), DIOPT (28) and Inparanoid (29) (Figure 1).

From these 6 types of indirect evidence, 36 feature values were computed to characterize the strength of functional associations between rat genes (Supplementary Table 2). Actually, not all 36 of these feature values are expected to separate the protein interactions from the random gene pairs. Here, the protein interactions were considered a strong type of functional association. To increase the prediction power in the later functional association inference step, we evaluated these feature values through the AUC in the ROC test. The ROC curve was used to assess whether the feature is capable of indicating protein interactions. Among the 36 features, 6 with AUC >0.6 were selected for the subsequent inference of functional gene associations (Supplementary Table 3 and Supplementary Figure 1).

Protein–protein interactions are one type of strong functional association. Here, we preferred to use protein interactions as positive examples in model training. Experimentally reported protein–protein interactions in rats were integrated from three databases, which included RGD (14), BioGRID (15) and IntAct (16) (Figure 1 and Supplementary Table 1). To ensure the quality of collected protein interactions, we filtered by the evidence provided in each database, keeping the experimentally confirmed high-confidence protein interactions, which were used as positive examples in training the prediction model (Figure 1 and Supplementary Table 1).

### Prediction of functional gene associations

The functional association prediction model was built using the libSVM package, which is an integrated software package for support vector classification (30, 31). In the prediction model training, 2693 experimentally confirmed protein–protein interactions were collected from before the year 2018 and used as positive examples. Negative examples were those randomly generated gene pairs that do not overlap with the positive examples. In fact, two random gene pairs still had a low probability of having functional associations. To reduce the impact of few false positive examples existing in the negative examples, we set the negative to positive ratio in the training dataset to 100:1.

We used the soft-margin Gaussian kernel SVM algorithm to train the prediction model. A 5-fold cross-validation was implemented to optimize the kernel width parameter σ and soft-margin parameter C. With the optimized σ and C, all the training data were used to train the prediction model, which was then validated by the protein–protein interactions (published after 31 December 2017) and randomly generated negative examples (positive:negative = 1:100). The final optimized model showed a total of 302 693 functional associations with a sensitivity of 13.02% and a specificity of 99.98%. For comparison, we also evaluated how well the predicted interaction in MIST and STRING covered these new interactions. The results are shown in Supplementary Table 5.

Applying this model to all rat gene pairs produced 302 693 inferred functional associations. These inferred functional interactions together with the 3246 known protein interactions make the PRID dataset, which consists of 305 939 interactions. Based on the inferred 305 939 functional associations, we were also curious about the proportion of protein–protein interactions covered by the predicted functional interactome. Therefore, we solved the following equation:

\begin{align*} &{N_{{\rm{interactome}}}} \times {\rm{Sensitivity}} + ({N_{{\rm{all-pairs}}}} - {N_{{\rm{interactome}}}}) \nonumber\\ &\times \left( {1 - {\rm{specificity}}} \right) = {N_{{\rm{predict}}}}\end{align*}

In the equation, ${\rm{ }}{N_{{\rm{interactome}}}}$ is the expected number of all protein–protein interactions in rats; ${N_{all-pairs}}$ is the number of all gene pairs in rats; ${N_{predict}}$ is the number of predicted gene associations; sensitivity and specificity are the accuracy measures produced when the prediction model is validated with newly published protein interactions. Solving this equation gave an estimated size of rat protein interactome1.22 × 10^6^. Based on the estimated interactome size (1.22 × 10^6^) and the estimated sensitivity (13.02%, the conservative estimation from the training stage sensitivity 13.38% and the evaluation stage sensitivity 13.02%), the predicted interactions in PRID is expected to include 159 618 true protein interactions. Therefore, 52.59% of the PRID functional interactions (159 618 out of 302 693) are expected to represent protein interactions.

### Evaluation of functional gene association network

To assess the quality of our predicted functional association network of PRID, we evaluated how well it connects functional associated genes and compared its quality with five other existing rat interactomes, including Mentha (32), MIST (33), STRING (34), RGD (14) and HitPredict (35). In this study, the quality evaluation of those interactomes was measured by the accuracy to predict a gene’s function by this gene’s network neighbors. The PANTHER term enrichment tool (36) was used to measure the prediction accuracy of each interactome.

Because PRID was inferred based on data available before 2018 (31 December 2017), we collected 11 810 genes from the GO database (up to 1 August 2018) for which new annotations were added after 31 December 2017. These genes contained a total of 376 904 annotations, 113 105 of which were newly reported after 2018. Based on these genes and their annotations, we compared the performance of six interactomes by PANTHER to infer new GO biological processes. To evaluate the overall accuracy of the new annotation prediction, we used the precision–recall curve in this study. Precision measured the proportion of PANTHER reported annotations that were correct with the known annotations (all 376 904 annotations), while recall measured the proportion of PANTHER reported annotations that were covered with the newly added 113 105 annotations. In reality, the number of PANTHER reported annotations will change if we alter the significance cut-off in PANTHER. A loose cut-off in PANTHER will report more annotations but with a higher false positive rate. In contrast, a strict cut-off in PANTHER will give fewer new annotations but with higher precision. In general, a loose cut-off will lead to higher recall, and a strict cut-off will lead to lower recall. Therefore, the precision–recall curve has the advantage of displaying the precision and recall rates at different cut-offs.

**Figure 2.**
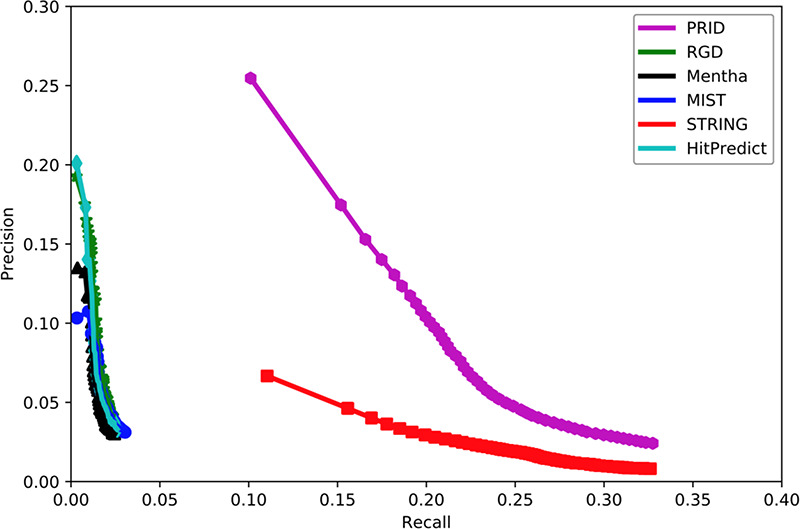
Assessment of six interactomes for their capabilities to group functionally related genes together. The precision–recall curves were drawn with a series of precision and recall pairs obtained by predicting a gene’s function with its network neighbors. Precision measures the fraction of correct annotations predicted using an interactome. Recall measures the fraction of new annotations predicted using an interactome.

Figure 2 shows that PRID had the highest AUC among six interactomes, suggesting its superior quality in gene function prediction compared with the remaining five interactomes. When the curves reached the high-recall region, PRID was the only database that maintained high precision. The curves of Mentha (32), MIST (33), RGD (14) and HitPredict (35) reached high-precision regions; however, these curves always remained in low-precision regions. However, STRING reached a high-recall region as did PRID, but its precision was always below PRID. This suggests that the STRING interactome may contain a large proportion of weak functional associations and result in a higher false positive rate when predicting the functional associations between rat genes. In conclusion, PRID showed the best performance with balanced coverage and accuracy during gene function prediction compared with the other interactomes.

### The web interface of PRID/GSLA

**Figure 3.**
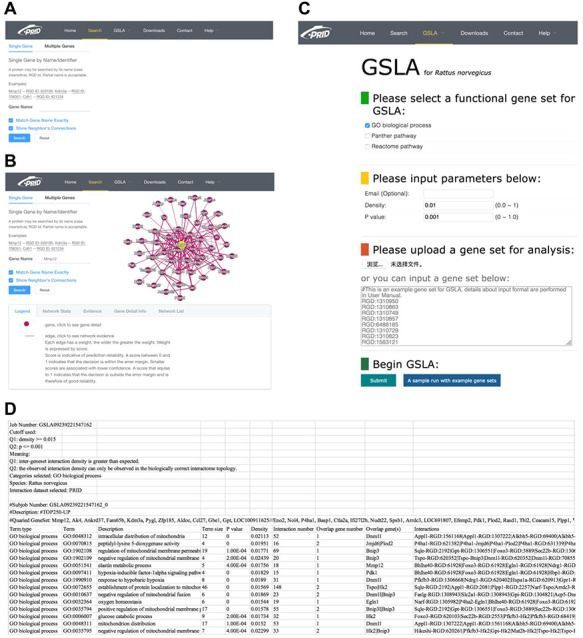
PRID website. (A) Single gene search and multiple gene search. (B) Search result page. Right-clicking on an interaction in the diagram will show its details. (C) GSLA interface. (D) GSLA result file.

We developed a user-friendly interface of PRID, which supports two search options: a single gene search and a multiple gene search (Figure 3A). The difference between these two search modes is the number of submitted genes. A single gene search provides the inferred functional associations involving the query gene, while a multiple gene search provides the functional associations between the query genes. The results reported by PRID are listed in a tabular form (Figure 3B). In addition, the inferred functional associations are also provided in a graphic view on the right side of the query interface. If users are curious about the detailed annotations of a gene in the predicted function association network, they can click the node corresponding to the gene of interest. We also provided the feature values that were used to predict the function associations; users can right-click the edges to see the details. All the reported functional associations in the predicted network are provided for users to download. Users can also download the full dump of the PRID interactome on the download page.

**Figure 4.**
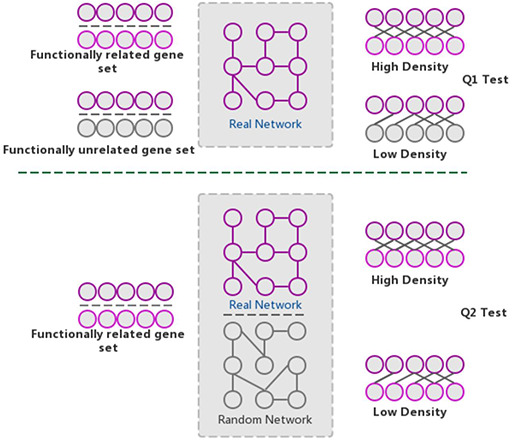
The GSLA algorithm. GSLA uses two hypothesis tests to identify biologically significant functional associations between gene sets. Q1 evaluates whether the intergene-set interaction density between two gene sets is higher than that between random gene pairs. Q2 evaluates whether the dense functional interactions between gene sets can only be observed within the correct network, rather than random interactomes.

The GSLA web tool was first developed based on the predicted Arabidopsis interactome resource (PAIR) to functionally interpret SCG in Arabidopsis (12). It evaluates whether a SCG has more frequent functional associations with genes that comprise a biological process or biological function. A pair of hypotheses (Q1 and Q2) was used to determine the statistical significance of functional associations between two rat genes (Figure 4). The first hypothesis (Q1) assumes that the density of intergene-set functional associations between two gene sets is higher than the density between two random gene sets. The second hypothesis (Q2) assumes that the density between two gene sets in a biologically meaningful functional gene association network is higher than in a random functional gene association network, which consists of the same genes and same neighbors but different interactions. In a biological sense, Q1 tests the strength of the functional association between two gene sets, while Q2 confirms that the strong functional association is the result of the biologically correct functional network (i.e. which represents our knowledge of molecular mechanisms) rather than a result of the gene set compositions. In reality, in an interactome, some genes, such as hubs, may have more neighbors than other genes. Gene sets containing many hubs may easily have more intergene-set functional associations compared with other gene sets. Therefore, we used Q2 to remove the confounding factor of gene set composition and ensure the biological significant functional associations between two gene sets. In general, Q1 and Q2 are different but complementary. They work together to increase the sensitivity and specificity of GSLA. For the GSLA web tool, the default significant cut-offs to report the interaction of a gene set are density > 0.01 (Q1) and *P* < 0.001 (Q2).

The PRID website also provides a link for users to access GSLA, which uses the PRID interactome to predict the functional impacts of SCG in rat transcriptomic experiments. The main interface is shown in Figure 3C. When users submit a set of SCGs, five types of rat gene IDs are supported, including RGD ID, gene name, UniProt ID, Ensembl gene ID, Ensembl protein ID and NCBI Entrez ID. Here, we suggest users provide SCG directly in RGD IDs because the internal server of GSLA works only with RGD ID so that the submitted IDs are automatically mapped to RGD ID before further computation (Figure 3D). Therefore, to avoid information loss, RGD ID is the best choice. In addition, the cut-offs for Q1 (density) and Q2 (*P*) are able to adjust (Figure 3C). To obtain specific and focused functional impacts, the top 50 to 200 genes of SCG are recommended. The results will be mailed to the user-provided email address. In the result file, the analysis parameters are shown in the top 10 lines, which are followed by a tabular list of identified biological processes and the functional gene interactions between the SCG and the reported biological processes (Figure 3D).

### Using the PRID/GSLA system to reanalyze the low oxygen embryogenesis datasets

During embryogenesis, the establishment of a trophoblast cell lineage is the leading cell differentiation event (37–40). The antecedents of all trophoblast cells, known as trophoblast stem (TS) cells, with their precise program of expansion and differentiation, are essential for the development of the hemochorial placenta (41), which plays an important role in fetal development, preservation of maternal health, and extraction of maternal resources (20). Placental organization plasticity can be achieved through differential regulation of TS cell proliferation and differentiation, which is affected by hypoxia. Low oxygen redirects placental organization, promotes the development of invasive trophoblast cells, remodels the uterine spiral artery and activates the cell response mediated by the HIF (20, 42–44). Chakraborty *et al*. reported that matrix metallopeptidase 12 (MMP12) is upregulated in low oxygen conditions (20). While no evidence supports that MMP12 is a direct target of HIF, the authors identified that lysine demethylase 3A (KDM3A) mediates the regulation of *Mmp12* by hypoxia/HIF. In summary, the authors found that the hypoxia-HIF-KDM3A-MMP12 regulatory pathway is conserved and facilitates placental adaptations.

**Figure 5.**
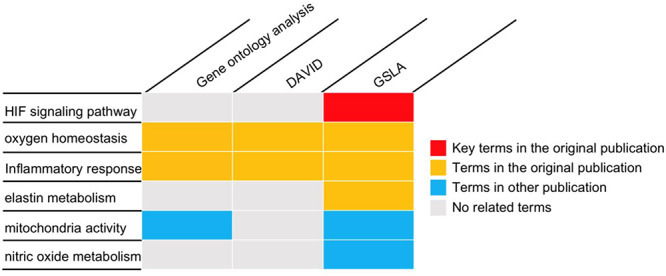
The gene set annotations produced by PRID/GSLA. The annotations produced by GSLA are more comprehensive and inspire further mechanism studies. We performed DAVID and gene ontology analysis for comparison.

To investigate placental adaptation and plasticity, Chakraborty *et al*. performed cDNA microarray analysis on rat TS cells with ambient (21% oxygen) or hypoxic (0.5% oxygen) conditions (GEO database, GSE80339) (20). They reported that 838 transcripts were downregulated, and 786 transcripts were upregulated under low oxygen conditions. We used the PRID/GSLA system to annotate the functional impacts of hypoxia-induced gene changes. The top 250 upregulated genes were analyzed with PRID/GSLA, DAVID and PANTHER (GO ontology analysis). All terms reported by these tools and the terms reported by the original article were summarized into functional categories in Supplementary Table 6. All three tools compared in this study reported oxygen homeostasis and inflammatory response-related biological processes, which were consistent with those documented in the original publication (Figure 5, Supplementary Tables 7–9). Notably, PRID/GSLA system reported HIF and MMP12-involved signaling pathways, which suggested the hypoxia-HIF-KDM3A-MMP12 regulatory circuit (Figure 5 and Supplementary Table 9), which are intuitively relevant to this study and provides a mechanism hypothesis. The results from PRID/GSLA system also showed elastin metabolism related pathways that were also reported in the original publication. In comparison with DAVID and PANTHER, PRID/GSLA reported mitochondrial-related terms, whereas DAVID did not (Supplementary Tables 8 and 9). Some other studies observed that hypoxic stress suppressed mitochondrial membrane potential and decreased total cytochrome c oxidase levels (45, 46). Furthermore, we also found a series of nitric oxide (NO)-related pathways with PRID/GSLA (Figure 5). In an independent study, Park *et al*. observed that hypoxia helped maintain NO production in trophoblast cells (47). In this case study, DAVID reported 193 terms in 34 clusters (Supplementary Table 7), GO ontology analysis reported 267 terms (Supplementary Table 8), in contrast, PRID/GSLA reported only 48 terms (Supplementary Table 9). Therefore, the interpretation provided by PRID/GSLA is broader and more concise, which highlights potentials for experimental researchers to explore molecular mechanisms, whereas other widely used enrichment-based tools did not provide similar insights.

## Discussion

As a powerful disease model organism, many efforts have been made to build a molecular interaction1 network of *Rattus norvegicus*. A comprehensive and accurate reference interactome of rats may facilitate hypothesis formulation in molecular pharmacology studies. To date, many rat interactome databases are available. Some of these databases integrate the experimentally reported protein–protein interactions, such as RGD (14), BioGrid (15) and IntAct (16). Others provided the predicted interactions of rats, including MIST (33), STRING (34) and HitPredict (35). In general, experimentally reported molecular interactions are considered to be more accurate than the predicted interactions. However, the number of experimentally reported interactions is too small. According to the estimated size of the rat interactome (1.22x 10^6^), the highest coverage of protein interactions is the RGD database (up to 1.08%), which represents 7.89% of the rat protein interactome. Actually, the coverage of the RGD database will be lower than 1.08% due to false positive experimental interactions. Therefore, researchers can only obtain limited support from experimental interactions for molecular mechanism analysis. In contrast, databases with predicted interactions may cover a high proportion of the true rat protein interactome. For example, STRING presents 6 624 008 rat gene associations and is estimated to cover as high as 14.83% of the rat interactome. However, these associations cover many types of reported associations (not only protein interactions), and only ∼0.86% of these associations may represent protein interactions. In our new assessment of functional prediction, both the experimentally reported interactomes and the predicted interactomes did not perform as well as the PRID interactome, which showed a balanced coverage and reliability (13.02% coverage and 52.59% reliability if evaluated as a protein interaction network). In conclusion, PRID complements existing rat interactomes as a high-quality network for functional gene association analysis.

The high quality of functional associations in PRID enables the GSLA approach to interpret the SCG in rats. To report significant functional associations between two gene sets, GSLA estimates the density of functional gene interactions between the component genes in two gene sets. Therefore, for the successful application of this strategy, a high-quality reference interactome with balanced coverage and accuracy was required. As discussed above, previous interactomes were unable to meet this need. If we used other interactomes, GSLA could not produce an interpretation of functional impacts as useful as those produced by PRID (data not shown). This was the same case when we assessed our previously developed high-quality functional interactome with other existing databases for humans and Arabidopsis (11, 13).

Our developed PRID/GSLA system extends the capability of current enrichment-based tools to interpret the potential functional impacts from the observed rat SCG and summarize the SCG into known biological processes, especially when no established biological concept can describe the observed SCG, as mentioned in the introduction. Moreover, the functional association resource provided in PRID is a useful reference for researchers for interpreting the molecular mechanisms of their genes of interest.

## Supplementary Material

baaa086_Supp
