# Supplementary material for: Predicted rat interactome database and gene set linkage analysis
Source: Database (Oxford). 2020 Nov 20;2020:baaa086. doi: 10.1093/database/baaa086 (PMC7678787; doi:10.1093/database/baaa086)
Supplement: baaa086_Supp [file baaa086_supp.zip › Table_S1.pdf]

Supplementary Table 1. Number of protein interactions and their component proteins collected from three databases.

Database	All interactions		High confidence interactions*		Date
	Number of interactions	Number of Proteins	Number of interactions	Number of Proteins	
BioGrid	4058	2276	1510	1102	Dec 25th, 2017
Intact	5241	2294	1231	986	Dec 12th, 2017
RGD	3871	1887	1166	829	Jun 27th,2017
Total	6251	3257	2693	2452	

*High confidence interactions: interactions reported in more than two independent studies, at least one of which is low-throughput experiment.
