# Supplementary material for: Predicted rat interactome database and gene set linkage analysis
Source: Database (Oxford). 2020 Nov 20;2020:baaa086. doi: 10.1093/database/baaa086 (PMC7678787; doi:10.1093/database/baaa086)
Supplement: baaa086_Supp [file baaa086_supp.zip › Table_S2.pdf]

Supplementary Table 2. Evidence of functional association and methods to compute feature values.

Feature	Data source (link) / Data set		Statistics
Shared Annotation	GOC (http://geneontology.org/docs/download-go-annotations/)	Cellular component<br>Biological process<br>Molecular function	Maximum Shared Annotation Score
Homologous Interactions	Inparanoid Score (http://inparanoid.sbc.su.se)		Maximum
Domain Interaction	IDDI (http://pcode.kaist.ac.kr/iddi/)<br>Pfam (http://pfam.xfam.org/)	3DID<br>IPFAM<br>PINS<br>TW<br>HIMAP<br>DOMAINGA<br>PVALUE<br>IPPRI<br>RCDP<br>DIPD<br>RDFF<br>DPEA<br>ME<br>PE<br>DIMA_STRING<br>KGIDDI<br>LLZ<br>GPE<br>DIMA_DPROF<br>APMM<br>TOPDOWN<br>DIMA_DPEA<br>INTERDOM	Maximum Shared Domain Score
Co-localization	Compartments (https://compartments.jensenlab.org/)	Knowledge channel<br>Experiments channel<br>Text mining channel<br>Predictions channel	Cosine Similarity Score
Co-expression	COXPRESdb (https://coxpresdb.jp/)	Microarray<br>RNASeq	Pearson's Correlation Score
Phylogenetic Profile	DIOPT (http://www.flyrnai.org/diopt)		Mutual Information Score<br>Pearson's Correlation Score<br>Tanimato Correlation Score
