# Supplementary material for: Predicted rat interactome database and gene set linkage analysis
Source: Database (Oxford). 2020 Nov 20;2020:baaa086. doi: 10.1093/database/baaa086 (PMC7678787; doi:10.1093/database/baaa086)
Supplement: baaa086_Supp [file baaa086_supp.zip › Table_S3.pdf]

Supplementary Table 3. Assessment of feature value qualities.

Evidence	Type feature	AUC of ROC	AUC > 0.6	Mark
Shared Annotation	biological_process_value	0.833	Yes	↓ [a]
	molecular_function_value	0.791	Yes	↓
	cellular_component_value	0.821	Yes	↓
Coexpression	RNAseq_value	0.758	Yes	↑ [b]
	Microarray_value	0.565	No	↑
Phylogenetic profile	phyprofile_tanimato_valu	0.682	Yes	↑
	phyprofile_pearson_value	0.431	No	↑
	phyprofile_mutual_value	0.513	No	↑
Colocation	integrated_value	0.582	No	↑
	knowledge_value	0.565	No	↑
	predictions_value	0.550	No	↑
	textmining_value	0.540	No	↑
Domain Interactions	INTERDOM_value	0.661	Yes	↑
	LLZ_value	0.510	No	↑
	RDFF_value	0.531	No	↑
	DIPD_value	0.519	No	↑
	3DID_value	0.544	No	↑
	PINS_value	0.527	No	↑
	IPPRI_value	0.512	No	↑
	ME_value	0.545	No	↑
	KGIDDI_value	0.560	No	↑
	APMM_value	0.578	No	↑
	IPFAM_value	0.530	No	↑
	DOMAINGA_value	0.501	No	↑
	RCDP_value	0.515	No	↑
	TOPDOWN_value	0.550	No	↑
	DIMA_DPEA_value	0.547	No	↑
	GPE_value	0.572	No	↑
	DIMA_DPROF_value	0.534	No	↑
	PVALUE_value	0.506	No	↑
	TW_value	0.501	No	↑
	HIMAP_value	0.508	No	↑
	PE_value	0.528	No	↑
	DIMA_STRING_value	0.522	No	↑
	DPEA_value	0.505	No	↑
Homologous interactions	inparanoid_value	0.577	No	↑

[a] ↓ : Lower score provides evidence of interaction

[b] ↑ : Higher score provides evidence of interaction
