# Supplementary material for: Predicted rat interactome database and gene set linkage analysis
Source: Database (Oxford). 2020 Nov 20;2020:baaa086. doi: 10.1093/database/baaa086 (PMC7678787; doi:10.1093/database/baaa086)
Supplement: baaa086_Supp [file baaa086_supp.zip › Table_S4.pdf]

Supplementary Table 4: The top 250 up-regulated genes used in microarray data analysis.

Gene symbol	Gene title	adj.P.Val	P.Value	logFC
Mmp12	matrix metallopeptidase 12	3.56E-07	1.14E-11	3.69
Ak4	adenylate kinase 4	9.10E-07	1.65E-10	2.74
Ankrd37	ankyrin repeat domain 37	9.10E-07	1.59E-10	4.63
Fam65b	family with sequence similarity 65, member B	9.10E-07	6.50E-11	3.16
Kdm3a	lysine demethylase 3A	9.10E-07	1.83E-10	2.76
Pygl	phosphorylase, glycogen, liver	9.10E-07	1.12E-10	2.73
Zfp185	zinc finger protein 185	9.10E-07	2.05E-10	4.07
Aldoc	aldolase, fructose-bisphosphate C	1.33E-06	5.54E-10	2.51
Ccl27	C-C motif chemokine ligand 27	1.33E-06	4.21E-10	2.53
Gbe1	glucan (1,4-alpha-), branching enzyme 1	1.33E-06	5.00E-10	2.61
Gpt	glutamic--pyruvic transaminase	1.33E-06	3.46E-10	2.64
LOC100911625///Eno2	gamma-enolase-like///enolase 2, gamma, neuronal	1.33E-06	5.63E-10	2.56
Nol4	nucleolar protein 4	1.33E-06	4.37E-10	2.6
P4ha1	prolyl 4-hydroxylase subunit alpha 1	1.37E-06	7.04E-10	2.32
Basp1	brain abundant, membrane attached signal protein 1	1.39E-06	9.54E-10	2.73
Ctla2a	cytotoxic T lymphocyte-associated protein 2 alpha	1.39E-06	9.24E-10	2.6
Ifi27l2b	interferon, alpha-inducible protein 27 like 2B	1.39E-06	9.01E-10	2.28
Nudt22	nudix hydrolase 22	1.39E-06	9.84E-10	2.37
Spsb1	splA/ryanodine receptor domain and SOCS box containing 1	1.39E-06	9.25E-10	2.33
Arrdc3	arrestin domain containing 3	1.47E-06	1.13E-09	2.43
LOC691807	hypothetical protein LOC691807	1.47E-06	1.10E-09	2.1
Efemp2	EGF-containing fibulin-like extracellular matrix protein 2	1.75E-06	1.63E-09	2.02
Pdk1	pyruvate dehydrogenase kinase 1	1.75E-06	1.48E-09	2.3
Plod2	procollagen lysine, 2-oxoglutarate 5-dioxygenase 2	1.75E-06	1.52E-09	3.58
Rasd1	ras related dexamethasone induced 1	1.75E-06	1.62E-09	2.26
Tbl2	transducin (beta)-like 2	1.82E-06	1.81E-09	2.19
Ceacam15	carcinoembryonic antigen-related cell adhesion molecule 15	1.90E-06	1.96E-09	2.08
Plpp1	phospholipid phosphatase 1	2.01E-06	2.20E-09	2.29
Vegfa	vascular endothelial growth factor A	2.01E-06	2.16E-09	1.83
Dlc1	DLC1 Rho GTPase activating protein	2.06E-06	2.32E-09	1.96
Il1r2	interleukin 1 receptor type 2	2.27E-06	2.63E-09	1.79
Il33	interleukin 33	2.65E-06	3.15E-09	1.94
Ddit4	DNA-damage-inducible transcript 4	3.02E-06	3.69E-09	2.61
Asic1	acid sensing ion channel subunit 1	3.03E-06	3.89E-09	2.14
Dpp3l///LOC678760///Dpp3	dipeptidylpeptidase 3-like///similar to Dipeptidyl-peptidase 3 (Dipeptidyl-peptidase III) (DPP III) (Dipeptidyl aminopeptidase III) (Dipeptidyl arylamidase III)///dipeptidylpeptidase 3	3.03E-06	3.97E-09	1.81
Stbd1	starch binding domain 1	3.03E-06	4.10E-09	2.52
RGD1309104	similar to RIKEN cDNA 1700025G04 gene	3.23E-06	4.47E-09	2.12
Slc25a36	solute carrier family 25 member 36	3.26E-06	4.61E-09	1.97
Acp5	acid phosphatase 5, tartrate resistant	4.10E-06	5.93E-09	1.87
Cd200	Cd200 molecule	4.39E-06	6.64E-09	1.94
Slc35e1	solute carrier family 35, member E1	4.39E-06	6.61E-09	1.56
Ppp1r3c	protein phosphatase 1, regulatory subunit 3C	4.86E-06	7.50E-09	4.71
Bnip3	BCL2/adenovirus E1B interacting protein 3	5.07E-06	7.99E-09	1.75
Nr4a3	nuclear receptor subfamily 4, group A, member 3	5.34E-06	8.59E-09	2.09
Bhlhe40	basic helix-loop-helix family, member e40	5.37E-06	1.05E-08	3.16
Egln1	egl-9 family hypoxia-inducible factor 1	5.37E-06	1.04E-08	1.74
Il33	interleukin 33	5.37E-06	8.84E-09	2.25
Mllt11	myeloid/lymphoid or mixed-lineage leukemia; translocated to, 11	5.37E-06	9.09E-09	1.56
Narf	nuclear prelamin A recognition factor	5.37E-06	1.03E-08	1.51
Pfkfb3	6-phosphofructo-2-kinase/fructose-2,6-biphosphatase 3	5.37E-06	1.02E-08	1.61
Rimklb	ribosomal modification protein rimK-like family member B	5.37E-06	1.02E-08	2.43
Tep1	telomerase associated protein 1	5.37E-06	9.40E-09	1.57
Wwp1	WW domain containing E3 ubiquitin protein ligase 1	5.37E-06	1.05E-08	1.45
Rcbtb1	RCC1 and BTB domain containing protein 1	5.46E-06	1.11E-08	1.62
Rccl1	RCC1 like	5.98E-06	1.25E-08	1.76
Greb1l	growth regulation by estrogen in breast cancer 1 like	6.02E-06	1.30E-08	1.77
Pgm1	phosphoglucomutase 1	6.02E-06	1.30E-08	1.66
Guca1a	guanylate cyclase activator 1A	6.22E-06	1.40E-08	2.85
Phtf1	putative homeodomain transcription factor 1	6.22E-06	1.38E-08	2.05
Vsir	V-set immunoregulatory receptor	6.62E-06	1.51E-08	1.64
Bcl11a	B-cell CLL/lymphoma 11A	6.74E-06	1.58E-08	1.58
Casp6	caspase 6	6.74E-06	1.61E-08	1.44
Cdcp1	CUB domain containing protein 1	6.74E-06	1.80E-08	1.7
Fam46a	family with sequence similarity 46, member A	6.74E-06	1.84E-08	2.12
Foxo3	forkhead box O3	6.74E-06	1.73E-08	2.16

Hk2	hexokinase 2	6.74E-06	1.67E-08	1.95
Kdr	kinase insert domain receptor	6.74E-06	1.74E-08	1.73
Ndrg1	N-myc downstream regulated 1	6.74E-06	1.78E-08	1.37
Pgm1	phosphoglucomutase 1	6.74E-06	1.84E-08	1.65
Ppm1j	protein phosphatase, Mg2+/Mn2+ dependent, 1J	6.74E-06	1.76E-08	1.59
Prepl	prolyl endopeptidase-like	6.74E-06	1.72E-08	1.7
Spidr	scaffolding protein involved in DNA repair	6.74E-06	1.61E-08	2.09
Cdcp1	CUB domain containing protein 1	7.18E-06	2.13E-08	1.54
Efemp2	EGF-containing fibulin-like extracellular matrix protein 2	7.18E-06	2.04E-08	1.81
Mmp9	matrix metallopeptidase 9	7.18E-06	2.07E-08	1.4
Proser2	proline and serine rich 2	7.18E-06	2.08E-08	1.88
Vldlr	very low density lipoprotein receptor	7.51E-06	2.27E-08	2.83
Gng2	G protein subunit gamma 2	7.57E-06	2.36E-08	1.5
Hspa1b///Hspa1a	heat shock 70kD protein 1B (mapped)///heat shock 70kD protein 1A	7.75E-06	2.49E-08	1.37
Mysm1	myb-like, SWIRM and MPN domains 1	7.75E-06	2.49E-08	1.46
Slc22a5	solute carrier family 22 member 5	7.75E-06	2.44E-08	2.02
Ell	elongation factor for RNA polymerase II	8.92E-06	3.01E-08	1.33
Greb1l	growth regulation by estrogen in breast cancer 1 like	8.92E-06	3.00E-08	1.41
Noct	nocturnin	9.29E-06	3.22E-08	1.43
Vwa1	von Willebrand factor A domain containing 1	9.34E-06	3.27E-08	2.61
Ero1a	endoplasmic reticulum oxidoreductase 1 alpha	9.61E-06	3.57E-08	1.28
Hilpda	hypoxia inducible lipid droplet-associated	9.61E-06	3.52E-08	1.37
Il1rap	interleukin 1 receptor accessory protein	9.61E-06	3.74E-08	1.66
Klc4	kinesin light chain 4	9.61E-06	3.72E-08	1.7
Parp16	poly (ADP-ribose) polymerase family, member 16	9.61E-06	3.53E-08	1.37
Scrn1	secernin 1	9.85E-06	3.89E-08	2.3
Mab21l3	mab-21 like 3	9.86E-06	3.95E-08	2.46
Vldlr	very low density lipoprotein receptor	9.86E-06	3.96E-08	2.69
P4ha2	prolyl 4-hydroxylase subunit alpha 2	1.03E-05	4.16E-08	1.5
Ddx50	DEAD-box helicase 50	1.09E-05	4.55E-08	1.29
LOC100911293///LOC691658///Aph1b	gamma-secretase subunit APH-1B-like///similar to anterior pharynx defective 1b homolog///aph-1 homolog B, gamma secretase subunit	1.09E-05	4.47E-08	1.36
Rnf138	ring finger protein 138	1.09E-05	4.58E-08	1.37
Vegfa	vascular endothelial growth factor A	1.11E-05	4.75E-08	1.93
Abhd18	abhydrolase domain containing 18	1.16E-05	5.07E-08	1.22
Efna1	ephrin A1	1.22E-05	5.51E-08	1.39
Higd1a	HIG1 hypoxia inducible domain family, member 1A	1.22E-05	5.58E-08	1.23
LOC102550354	uncharacterized LOC102550354	1.22E-05	5.69E-08	2.57
Smtnl2	smoothelin-like 2	1.22E-05	5.72E-08	1.72
Zc3h7b	zinc finger CCCH-type containing 7B	1.22E-05	5.68E-08	1.23
Cpm	carboxypeptidase M	1.25E-05	5.93E-08	2.22
Sec22b	SEC22 homolog B, vesicle trafficking protein	1.25E-05	6.02E-08	1.24
Tiparp	TCDD-inducible poly(ADP-ribose) polymerase	1.25E-05	6.00E-08	1.37
LOC501445///Derl1	derlin-1 pseudogene///derlin 1	1.27E-05	6.16E-08	1.3
Dusp1	dual specificity phosphatase 1	1.28E-05	6.29E-08	1.19
Adamts9	ADAM metallopeptidase with thrombospondin type 1 motif, 9	1.30E-05	6.59E-08	1.31
Eif2ak4	eukaryotic translation initiation factor 2 alpha kinase 4	1.30E-05	6.83E-08	1.14
Higd1a	HIG1 hypoxia inducible domain family, member 1A	1.30E-05	6.82E-08	1.56
Id3	inhibitor of DNA binding 3, HLH protein	1.30E-05	6.82E-08	1.75
Map3k8	mitogen-activated protein kinase kinase kinase 8	1.30E-05	6.61E-08	1.82
Slc27a1	solute carrier family 27 member 1	1.30E-05	6.70E-08	1.48
Bnip3l	BCL2/adenovirus E1B interacting protein 3-like	1.32E-05	7.19E-08	1.21
Fbln1	fibulin 1	1.32E-05	7.13E-08	1.51
Insig2	insulin induced gene 2	1.32E-05	6.94E-08	1.45
Mex3b	mex-3 RNA binding family member B	1.32E-05	7.06E-08	1.64
Nptx1	neuronal pentraxin 1	1.32E-05	7.40E-08	1.57
Ramp3	receptor (G protein-coupled) activity modifying protein 3	1.32E-05	7.34E-08	1.55
RGD1560795///Spr	similar to Sepiapterin reductase (SPR)///sepiapterin reductase (7,8-dihydrobiopterin:NADP+ oxidoreductase)	1.32E-05	7.41E-08	1.48
RGD1562037	similar to OTTHUMP00000046255	1.32E-05	7.18E-08	1.14
Spsb1	splA/ryanodine receptor domain and SOCS box containing 1	1.32E-05	7.48E-08	2.2
Insig1	insulin induced gene 1	1.34E-05	7.69E-08	1.74
Insig2	insulin induced gene 2	1.34E-05	7.82E-08	1.85
Sphk1	sphingosine kinase 1	1.44E-05	8.68E-08	1.57
Zfp503	zinc finger protein 503	1.45E-05	8.89E-08	1.59
Dnajb1	DnaJ heat shock protein family (Hsp40) member B1	1.46E-05	9.04E-08	1.1
Tspo	translocator protein	1.46E-05	8.96E-08	1.13
Plau	plasminogen activator, urokinase	1.50E-05	9.30E-08	1.09
Hp	haptoglobin	1.53E-05	9.53E-08	1.04
Rnf125	ring finger protein 125	1.53E-05	9.57E-08	2.04

Chpt1	choline phosphotransferase 1	1.57E-05	1.00E-07	1.32
Clcn3	chloride voltage-gated channel 3	1.66E-05	1.08E-07	1.47
Hmx1	H6 family homeobox 1	1.66E-05	1.08E-07	1.59
Clcn3	chloride voltage-gated channel 3	1.69E-05	1.10E-07	1.34
Eif4e3	eukaryotic translation initiation factor 4E family member 3	1.69E-05	1.11E-07	1.05
Nfil3	nuclear factor, interleukin 3 regulated	1.70E-05	1.12E-07	1.13
Bnip3l	BCL2/adenovirus E1B interacting protein 3-like	1.74E-05	1.15E-07	1.15
Acvrl1	activin A receptor like type 1	1.75E-05	1.18E-07	2.02
Gpr1	G protein-coupled receptor 1	1.75E-05	1.18E-07	1.2
Syne1	spectrin repeat containing nuclear envelope protein 1	1.75E-05	1.19E-07	1.47
Prkcq	protein kinase C, theta	1.76E-05	1.21E-07	1.32
Acap1	ArfGAP with coiled-coil, ankyrin repeat and PH domains 1	1.80E-05	1.26E-07	1.39
Eif4ebp1	eukaryotic translation initiation factor 4E binding protein 1	1.88E-05	1.33E-07	1.07
Hltf	helicase-like transcription factor	1.96E-05	1.39E-07	1.02
LOC100910685///Gcat	2-amino-3-ketobutyrate coenzyme A ligase, mitochondrial-like///glycine C-acetyltr	1.96E-05	1.40E-07	1.34
Pard6b	par-6 family cell polarity regulator beta	1.96E-05	1.41E-07	1.02
Wnt4	wingless-type MMTV integration site family, member 4	1.96E-05	1.41E-07	1.28
Cpm	carboxypeptidase M	1.99E-05	1.48E-07	1.87
LOC100909732///Fam122b	protein FAM122B-like///family with sequence similarity 122B	1.99E-05	1.49E-07	1.06
Maff	MAF bZIP transcription factor F	1.99E-05	1.48E-07	1.15
Mat2b	methionine adenosyltransferase 2B	2.01E-05	1.53E-07	1.07
Foxo4	forkhead box O4	2.03E-05	1.56E-07	1.66
Gpi	glucose-6-phosphate isomerase	2.05E-05	1.59E-07	1.22
LOC100912399///Map3k1	mitogen-activated protein kinase kinase kinase 1-like///mitogen-activated protein ki	2.06E-05	1.60E-07	9.98E-01
Appl1	adaptor protein, phosphotyrosine interacting with PH domain and leucine zipper 1	2.09E-05	1.64E-07	1.12
Atp11b	ATPase phospholipid transporting 11B (putative)	2.09E-05	1.64E-07	1.73
Slc2a1	solute carrier family 2 member 1	2.10E-05	1.69E-07	1.05
Tceal9	transcription elongation factor A like 9	2.15E-05	1.75E-07	1.02
Bcr	BCR, RhoGEF and GTPase activating protein	2.19E-05	1.80E-07	1.17
Arl16	ADP-ribosylation factor like GTPase 16	2.21E-05	1.83E-07	1.09
Gzf1	GDNF-inducible zinc finger protein 1	2.21E-05	1.84E-07	1.02
Cdkn1b	cyclin-dependent kinase inhibitor 1B	2.22E-05	1.87E-07	1.22
Hagh	hydroxyacyl glutathione hydrolase	2.22E-05	1.86E-07	9.83E-01
Hbp1	HMG-box transcription factor 1	2.23E-05	1.92E-07	1.09
Rsrp1	arginine and serine rich protein 1	2.23E-05	1.93E-07	1.34
Vldlr	very low density lipoprotein receptor	2.23E-05	1.90E-07	2.22
Zap70	zeta-chain (TCR) associated protein kinase 70	2.23E-05	1.93E-07	1.86
Arl16	ADP-ribosylation factor like GTPase 16	2.24E-05	1.94E-07	1.12
LOC100910137///Triap1	TP53-regulated inhibitor of apoptosis 1-like///TP53 regulated inhibitor of apoptosi	2.25E-05	1.97E-07	1.09
Hspa1a	heat shock 70kD protein 1A	2.28E-05	2.01E-07	1.44
Il2rb	interleukin 2 receptor subunit beta	2.28E-05	2.01E-07	1.17
Alkbh5	alkB homolog 5, RNA demethylase	2.31E-05	2.06E-07	9.84E-01
Comt	catechol-O-methyltransferase	2.41E-05	2.21E-07	1.23
Lrif1	ligand dependent nuclear receptor interacting factor 1	2.41E-05	2.22E-07	1.37
Mmp9	matrix metallopeptidase 9	2.41E-05	2.22E-07	1.02
Zmynd10	zinc finger, MYND-type containing 10	2.41E-05	2.22E-07	1.3
Rsrp1	arginine and serine rich protein 1	2.56E-05	2.38E-07	1.92
Tspan33	tetraspanin 33	2.56E-05	2.38E-07	9.77E-01
LOC683062///Vdac1	similar to voltage-dependent anion channel 1///voltage-dependent anion channel 1	2.71E-05	2.56E-07	1.05
Faslg	Fas ligand	2.78E-05	2.73E-07	1.52
Ero1a	endoplasmic reticulum oxidoreductase 1 alpha	2.82E-05	2.81E-07	1.89
Prkag2	protein kinase AMP-activated non-catalytic subunit gamma 2	2.82E-05	2.82E-07	1.18
Vopp1	vesicular, overexpressed in cancer, prosurvival protein 1	2.82E-05	2.79E-07	2.26
Ndrg1	N-myc downstream regulated 1	2.84E-05	2.85E-07	1.89
Prrc1	proline-rich coiled-coil 1	2.88E-05	2.89E-07	1.29
Zc3h7b	zinc finger CCCH-type containing 7B	2.88E-05	2.91E-07	1.07
Scpep1	serine carboxypeptidase 1	2.89E-05	2.93E-07	9.61E-01
Entpd2	ectonucleoside triphosphate diphosphohydrolase 2	2.93E-05	3.00E-07	1.15
Lrrc8a	leucine rich repeat containing 8 family, member A	2.93E-05	2.99E-07	1.14
Cr1l	complement component (3b/4b) receptor 1-like	2.94E-05	3.03E-07	9.25E-01
Hexb	hexosaminidase subunit beta	2.94E-05	3.04E-07	9.30E-01
Slc27a3	solute carrier family 27 member 3	2.95E-05	3.07E-07	9.24E-01
Arrdc3	arrestin domain containing 3	2.97E-05	3.14E-07	2.46
Kdm4b	lysine demethylase 4B	2.97E-05	3.10E-07	1.17
LOC102553917	putative zinc finger protein 724-like	2.97E-05	3.13E-07	1.09
Entpd4	ectonucleoside triphosphate diphosphohydrolase 4	2.99E-05	3.19E-07	9.61E-01
Prkag2	protein kinase AMP-activated non-catalytic subunit gamma 2	3.00E-05	3.25E-07	1.31
Ccng2	cyclin G2	3.01E-05	3.29E-07	1.98
Dst	dystonin	3.01E-05	3.29E-07	1.29
Ksr1	kinase suppressor of ras 1	3.01E-05	3.30E-07	9.32E-01

Pdk1	pyruvate dehydrogenase kinase 1	3.10E-05	3.44E-07	1.56
LOC100911874///Slc40a1	solute carrier family 40 member 1-like///solute carrier family 40 member 1	3.22E-05	3.65E-07	9.02E-01
Bcl6	B-cell CLL/lymphoma 6	3.32E-05	3.79E-07	1.59
Dnm1l	dynamin 1-like	3.35E-05	3.88E-07	1.03
Rybp	RING1 and YY1 binding protein	3.35E-05	3.88E-07	1.01
Jmjd6	jumonji domain containing 6	3.36E-05	3.91E-07	1
Bmt2	base methyltransferase of 25S rRNA 2 homolog	3.39E-05	4.00E-07	9.21E-01
Hikeshi	Hikeshi, heat shock protein nuclear import factor	3.45E-05	4.16E-07	8.73E-01
Mnt	MAX network transcriptional repressor	3.46E-05	4.21E-07	9.06E-01
Slc6a8	solute carrier family 6 member 8	3.46E-05	4.26E-07	9.34E-01
Tomm7	translocase of outer mitochondrial membrane 7	3.46E-05	4.22E-07	8.75E-01
Pfkl	phosphofructokinase, liver type	3.51E-05	4.40E-07	1.31
Asic1	acid sensing ion channel subunit 1	3.58E-05	4.54E-07	1.46
Dnajc3	DnaJ heat shock protein family (Hsp40) member C3	3.58E-05	4.53E-07	9.94E-01
Tpgs2	tubulin polyglutamylase complex subunit 2	3.58E-05	4.54E-07	9.90E-01
Dtna	dystrobrevin, alpha	3.60E-05	4.59E-07	1.66
Fcna	ficolin A	3.60E-05	4.59E-07	1.46
Cited2	Cbp/p300-interacting transactivator, with Glu/Asp-rich carboxy-terminal domain, [illegible]	3.62E-05	4.72E-07	8.43E-01
Dennd3	DENN domain containing 3	3.62E-05	4.72E-07	1.06
Mllt3	MLLT3, super elongation complex subunit	3.62E-05	4.74E-07	9.92E-01
Khnyn	KH and NYN domain containing	3.64E-05	4.85E-07	1.29
Dnajb9	DnaJ heat shock protein family (Hsp40) member B9	3.68E-05	4.93E-07	1.15
Tspan31	tetraspanin 31	3.71E-05	5.04E-07	8.66E-01
Actn1	actinin, alpha 1	3.77E-05	5.16E-07	1.09
Cblb	Cbl proto-oncogene B	3.78E-05	5.22E-07	1.12
Upp1	uridine phosphorylase 1	3.78E-05	5.22E-07	1.31
Ifi27	interferon, alpha-inducible protein 27	3.81E-05	5.27E-07	9.51E-01
Slc16a3	solute carrier family 16 member 3	3.81E-05	5.28E-07	1.08
Kctd11	potassium channel tetramerization domain containing 11	3.86E-05	5.38E-07	1.18
LOC100911642///LOC680835	cullin-7-like///similar to cullin 7	3.93E-05	5.49E-07	9.65E-01
Plin3	perilipin 3	4.03E-05	5.74E-07	8.96E-01
Kazald1	Kazal-type serine peptidase inhibitor domain 1	4.07E-05	5.85E-07	1.3
Asic1	acid sensing ion channel subunit 1	4.09E-05	5.93E-07	1.62
Lrg1	leucine-rich alpha-2-glycoprotein 1	4.09E-05	5.93E-07	1.56
Pgk1	phosphoglycerate kinase 1	4.09E-05	5.94E-07	9.05E-01
Surf4	surfeit 4	4.15E-05	6.04E-07	9.17E-01
Arhgap8	Rho GTPase activating protein 8	4.17E-05	6.08E-07	9.94E-01
Ctsm	cathepsin M	4.20E-05	6.17E-07	1.25
Egln3	egl-9 family hypoxia-inducible factor 3	4.20E-05	6.17E-07	1.14
Xkr8	XK related 8	4.25E-05	6.29E-07	1.21
Mturn	maturin, neural progenitor differentiation regulator homolog (Xenopus)	4.28E-05	6.36E-07	1.61
Prrc1	proline-rich coiled-coil 1	4.34E-05	6.50E-07	8.27E-01
Sqle	squalene epoxidase	4.38E-05	6.61E-07	1.35
Clcn3	chloride voltage-gated channel 3	4.39E-05	6.67E-07	1.38
Tmem59	transmembrane protein 59	4.39E-05	6.67E-07	8.27E-01
Fiz1	FLT3-interacting zinc finger 1	4.41E-05	6.70E-07	1.14
Clcn3	chloride voltage-gated channel 3	4.45E-05	6.99E-07	1.47
