# Supplementary material for: Predicted rat interactome database and gene set linkage analysis
Source: Database (Oxford). 2020 Nov 20;2020:baaa086. doi: 10.1093/database/baaa086 (PMC7678787; doi:10.1093/database/baaa086)
Supplement: baaa086_Supp [file baaa086_supp.zip › Table_S5.pdf]

Supplementary Table 5. Evaluation of the predicted interactions in different datasets.

Network	Sensitivity	Reliability
MIST	0.54%	57.03%
**PRID**	**13.02%**	**52.59%**
STRING	14.83%	2.74%
