# Supplementary material for: Predicted rat interactome database and gene set linkage analysis
Source: Database (Oxford). 2020 Nov 20;2020:baaa086. doi: 10.1093/database/baaa086 (PMC7678787; doi:10.1093/database/baaa086)
Supplement: baaa086_Supp [file baaa086_supp.zip › Table_S6.pdf]

Supplementary Table 6. Summarization of terms that reported by the original paper and three tools.

Main functional categories of the terms	Original paper	DAVID	Gene ontology analysis	PRID/GSLA
HIF signaling pathway	Yes	No	No	Yes
oxygen homeostasis	Yes	Yes	Yes	Yes
inflammatory response	Yes	Yes	Yes	Yes
elastin metabolism	Yes	No	No	Yes
mitochondria activity	No	No	Yes	Yes
nitric oxide metabolism	No	No	No	Yes
regulation of development	Yes	Yes	Yes	Yes
extracellular matrix organization	Yes	Yes	Yes	Yes
carbohydrate metabolic process	Yes	Yes	Yes	Yes
lipid metabolic process	No	Yes	Yes	Yes
energy reserve metabolic process	No	Yes	Yes	No
ribonucleoside metabolic process	No	No	Yes	No
small molecule metabolic process	No	No	Yes	No
phosphorus metabolic process	No	No	Yes	No
cellular biosynthetic process	No	No	Yes	No
regulation of signal transduction	Yes	Yes	Yes	Yes
GTP binding	No	Yes	No	No
L-ascorbic acid binding	No	Yes	No	No
ion binding	No	Yes	No	No
proteolysis	Yes	Yes	Yes	Yes
catalytic activity	Yes	Yes	Yes	Yes
cell proliferation	Yes	Yes	Yes	Yes
cell differentiation	Yes	Yes	Yes	Yes
cell-cell adhesion	No	Yes	Yes	No
cell migration	Yes	Yes	Yes	Yes
cell communication	No	No	Yes	No
cellular component	No	Yes	Yes	No

circadian rhythm	No	Yes	No	No
regulation of transcription	Yes	Yes	Yes	No
autophagy	No	Yes	Yes	Yes
apoptotic process	Yes	No	Yes	Yes
cell death	Yes	No	Yes	Yes
aging	No	No	Yes	No
response to stress	No	No	Yes	Yes
cellular response to stimulus	No	No	Yes	Yes
sensory perception of smell	No	No	Yes	No
plasma membrane	No	Yes	Yes	Yes
microtubule	No	Yes	No	No
female pregnancy	No	No	Yes	No
multi-multicellular organism process	No	No	Yes	No
