# Supplementary material for: Predicted rat interactome database and gene set linkage analysis
Source: Database (Oxford). 2020 Nov 20;2020:baaa086. doi: 10.1093/database/baaa086 (PMC7678787; doi:10.1093/database/baaa086)
Supplement: baaa086_Supp [file baaa086_supp.zip › Table_S7.pdf]

Supplementary Table 7. Annotations reported by DAVID for the top 250 transcriptionally changed genes.

Annotation Cluster 1 Enrichment Score: 3.168883685307409

Category	Term	Count	%	PValue	Genes	List Total	Pop Hits	Pop Total	Fold Enrichment	Bonferroni	Benjamini	FDR
UP_KEYWORDS	Dioxygenase	8	3.827751196	3.16E-07	P4HA2, P4HA1, JMJD6, PLOD2, EGLN3, KDM3A, EGLN1, ALKBH5	206	46	20793	17.5542423	6.44E-05	6.44E-05	3.97E-04
INTERPRO	IPR006620:Prolyl 4-hydroxylase, alpha subunit	5	2.392344498	1.75E-05	P4HA2, P4HA1, PLOD2, EGLN3, EGLN1	199	16	19384	30.43969849	0.00818954	0.00818954	0.02492752
GOTERM_BP_DIRECT	GO:0018401~peptidyl-proline hydroxylation to 4-hydroxy-L-proline	4	1.913875598	2.08E-05	P4HA2, P4HA1, EGLN3, EGLN1	181	6	17535	64.58563536	0.02742495	0.00922652	0.03407123
SMART	SM00702:P4Hc	5	2.392344498	2.41E-05	P4HA2, P4HA1, PLOD2, EGLN3, EGLN1	109	16	9680	27.75229358	0.00273855	0.00273855	0.02735347
GOTERM_MF_DIRECT	GO:0031418~L-ascorbic acid binding	5	2.392344498	4.79E-05	P4HA2, P4HA1, PLOD2, EGLN3, EGLN1	174	20	16650	23.92241379	0.01784695	0.01784695	0.06617909
INTERPRO	IPR005123:Oxoglutarate/iron-dependent dioxygenase	5	2.392344498	5.52E-05	P4HA2, P4HA1, PLOD2, EGLN3, EGLN1	199	21	19384	23.19215123	0.02564655	0.01290656	0.07873656
UP_KEYWORDS	Vitamin C	4	1.913875598	3.17E-04	P4HA1, PLOD2, EGLN3, EGLN1	206	14	20793	28.83911234	0.06268309	0.02134681	0.39822557
UP_SEQ_FEATURE	domain:Fe2OG dioxygenase	4	1.913875598	3.58E-04	P4HA1, PLOD2, EGLN3, EGLN1	108	11	7976	26.85521886	0.13504881	0.13504881	0.49931052
UP_SEQ_FEATURE	binding site:2-oxoglutarate	3	1.435406699	0.001736591	P4HA1, EGLN3, EGLN1	108	5	7976	44.31111111	0.50536287	0.29669556	2.39944018
UP_SEQ_FEATURE	metal ion-binding site:Iron	4	1.913875598	0.017296043	P4HA1, PLOD2, EGLN3, EGLN1	108	41	7976	7.205058717	0.99914652	0.90514393	21.6353156
GOTERM_MF_DIRECT	GO:0005506~iron ion binding	7	3.349282297	0.019078084	P4HA2, P4HA1, JMJD6, PLOD2, EGLN3, KDM3A, EGLN1	174	202	16650	3.31597815	0.9992846	0.70094016	23.3754896
GOTERM_MF_DIRECT	GO:0016706~oxidoreductase activity, acting on paired donors, with incorporation or reduction of molecular oxygen, 2-oxoglutarate as one donor, and incorporation of one atom each of oxygen into both donors	3	1.435406699	0.021635695	EGLN3, EGLN1, ALKBH5	174	22	16650	13.04858934	0.99973194	0.6422924	26.09134
UP_KEYWORDS	Iron	8	3.827751196	0.022174605	P4HA1, JMJD6, PLOD2, EGLN3, ACP5, KDM3A, EGLN1, ALKBH5	206	282	20793	2.863457963	0.98968884	0.23592543	24.570731
UP_KEYWORDS	Oxidoreductase	10	4.784688995	0.056792622	ERO1A, P4HA2, SQLE, P4HA1, JMJD6, PLOD2, EGLN3, KDM3A, EGLN1, ALKBH5	206	494	20793	2.043256947	0.9999934	0.37942444	52.0602716
GOTERM_BP_DIRECT	GO:0055114~oxidation-reduction process	10	4.784688995	0.2265724	ERO1A, P4HA2, SQLE, P4HA1, JMJD6, PLOD2, EGLN3, KDM3A, EGLN1, HIGD1A	181	651	17535	1.48814828	1	0.97207383	98.5145596

Annotation Cluster 2 Enrichment Score: 2.196893822653743

Category	Term	Count	%	PValue	Genes	List Total	Pop Hits	Pop Total	Fold Enrichment	Bonferroni	Benjamini	FDR
UP_KEYWORDS	Glycolysis	5	2.392344498	3.78E-04	GPI, PFKL, ALDOC, HK2, PGK1	206	35	20793	14.41955617	0.07424598	0.01531092	0.47440496
KEGG_PATHWAY	rno00500:Starch and sucrose metabolism	5	2.392344498	4.04E-04	GPI, GBE1, PYGL, PGM1, HK2	90	31	7749	13.88709677	0.05990491	0.03041499	0.48263125
GOTERM_BP_DIRECT	GO:0006096~glycolytic process	5	2.392344498	6.67E-04	GPI, PFKL, ALDOC, HK2, PGK1	181	39	17535	12.42031449	0.59028676	0.13818642	1.08749955
KEGG_PATHWAY	rno00010:Glycolysis / Gluconeogenesis	6	2.870813397	0.001201016	GPI, PFKL, ALDOC, PGM1, HK2, PGK1	90	70	7749	7.38	0.16795266	0.04492603	1.42967151
KEGG_PATHWAY	rno00030:Pentose phosphate pathway	4	1.913875598	0.004312485	GPI, PFKL, ALDOC, PGM1	90	29	7749	11.87586207	0.48378759	0.09013807	5.046828
KEGG_PATHWAY	rno00051:Fructose and mannose metabolism	4	1.913875598	0.008584949	PFKL, PFKFB3, ALDOC, HK2	90	37	7749	9.308108108	0.7326423	0.12358607	9.8156034
GOTERM_BP_DIRECT	GO:0005975~carbohydrate metabolic process	6	2.870813397	0.009398149	HAGH, GPI, PFKL, HEXB, PGM1, PGK1	181	125	17535	4.650165746	0.99999671	0.50409684	14.333511
KEGG_PATHWAY	rno01200:Carbon metabolism	6	2.870813397	0.012508273	GPI, PFKL, ALDOC, HK2, GPT, PGK1	90	121	7749	4.269421488	0.85424606	0.16060673	14.0002997

KEGG_PATHWAY	rno01130:Biosynthesis of antibiotics	8	3.827751196	0.012698314	GPI, PFKL, SQLE, ALDOC, PGM1, HK2, AK4, PGK1	90	219	7749	3.145205479	0.85847557	0.15035804	14.198408
KEGG_PATHWAY	rno01230:Biosynthesis of amino acids	5	2.392344498	0.013823865	PFKL, ALDOC, GPT, MAT2B, PGK1	90	81	7749	5.314814815	0.88113971	0.15111476	15.3631859
KEGG_PATHWAY	rno00520:Amino sugar and nucleotide sugar metabolism	4	1.913875598	0.017461051	GPI, HEXB, PGM1, HK2	90	48	7749	7.175	0.93246707	0.15502332	19.0287707
KEGG_PATHWAY	rno00052:Galactose metabolism	3	1.435406699	0.051789105	PFKL, PGM1, HK2	90	32	7749	8.071875	0.99970728	0.30914904	47.1235722
UP_SEQ_FEATURE	binding site:Substrate	5	2.392344498	0.326268504	PFKL, JMJD6, ALDOC, COMT, PGK1	108	215	7976	1.717484927	1	0.99995444	99.5987638
Annotation Cluster 3	Enrichment Score: 1.9520159924652232											
Category	Term	Count	%	PValue	Genes	List Total	Pop Hits	Pop Total	Fold Enrichment	Bonferroni	Benjamini	FDR
UP_KEYWORDS	Kinase	17	8.133971292	3.70E-04	PDK1, ACVRL1, PFKL, PFKFB3, PRKAG2, SPHK1, HK2, AK4, KDR, PRKCQ, CDKN1B, MAP3K8, ZAP70, PGK1, KSR1, EIF2AK4, PLAU	206	609	20793	2.817614424	0.07280446	0.01872026	0.46485929
UP_KEYWORDS	Transferase	25	11.96172249	0.002689401	PDK1, ACVRL1, PFKL, PFKFB3, SPHK1, PRKAG2, HK2, UPP1, AK4, COMT, CHPT1, BMT2, KDR, PARP16, PRKCQ, CDKN1B, PYGL, WWP1, MAP3K8, ZAP70, GPT, KSR1, PGK1, EIF2AK4, PLAU	206	1316	20793	1.917494172	0.42269053	0.07548167	3.32967364
GOTERM_MF_DIRECT	GO:0005524~ATP binding	25	11.96172249	0.010494714	PDK1, CLCN3, ACVRL1, PFKL, PFKFB3, SPHK1, PRKAG2, HK2, ATP11B, HSPA1A, AK4, HLTF, KDR, RIMKLB, PRKCQ, PYGL, MAP3K8, DDX50, ZAP70, TEP1, SLC22A5, KSR1, PGK1, ENTPD2, EIF2AK4	174	1403	16650	1.705090078	0.9810673	0.73347525	13.5694396
UP_KEYWORDS	Nucleotide-binding	23	11.00478469	0.011795708	PDK1, SLC27A1, CLCN3, ACVRL1, DNM1L, PFKL, PFKFB3, SPHK1, HK2, ATP11B, HSPA1A, AK4, KDR, PRKCQ, PYGL, MAP3K8, ZAP70, TEP1, SLC22A5, PGK1, ENTPD2, RASD1, EIF2AK4	206	1327	20793	1.749471397	0.9111343	0.15878001	13.8610115
UP_KEYWORDS	ATP-binding	19	9.090909091	0.013446339	PDK1, CLCN3, ACVRL1, PFKL, PFKFB3, SPHK1, HK2, ATP11B, HSPA1A, AK4, KDR, PRKCQ, MAP3K8, ZAP70, TEP1, SLC22A5, PGK1, ENTPD2, EIF2AK4	206	1029	20793	1.863752158	0.93681335	0.16815638	15.6528695
UP_SEQ_FEATURE	nucleotide phosphate-binding region:ATP	12	5.741626794	0.038307795	PDK1, PRKCQ, CLCN3, ACVRL1, PFKL, PFKFB3, MAP3K8, TEP1, AK4, SLC22A5, PGK1, KDR	108	450	7976	1.969382716	0.99999987	0.95774056	42.0626332
UP_SEQ_FEATURE	binding site:ATP	6	2.870813397	0.341105313	PDK1, PRKCQ, ACVRL1, MAP3K8, PGK1, KDR	108	287	7976	1.543941154	1	0.99995174	99.7060546
Annotation Cluster 4	Enrichment Score: 1.5317619216130294											
Category	Term	Count	%	PValue	Genes	List Total	Pop Hits	Pop Total	Fold Enrichment	Bonferroni	Benjamini	FDR
INTERPRO	IPR002017:Spectrin repeat	3	1.435406699	0.022813956	SYNE1, ACTN1, DST	199	23	19384	12.70526546	0.99998098	0.97330505	28.0747734
INTERPRO	IPR018159:Spectrin/alpha-actinin	3	1.435406699	0.030821557	SYNE1, ACTN1, DST	199	27	19384	10.82300391	0.99999961	0.97493654	36.0480695

SMART	SM00150:SPEC	3	1.435406699	0.036112625	SYNE1, ACTN1, DST	109	27	9680	9.867482161	0.98489929	0.87711507	34.183192
Annotation Cluster 5	Enrichment Score: 1.3908261786922977											
Category	Term	Count	%	PValue	Genes	List Total	Pop Hits	Pop Total	Fold Enrichment	Bonferroni	Benjamini	FDR
UP_KEYWORDS	Glycoprotein	38	18.18181818	4.28E-04	SCPEP1, IL1R2, CLCN3, ACVRL1, LRRC8A, EFNA1, MMP9, FCNA, HEXB, ACP5, FASLG, HP, PLPP1, GPR1, WNT4, NPTX1, ERO1A, PLOD2, P4HA1, TSPAN31, IL1RAP, SLC2A1, SLC22A5, VWA1, ENTPD2, RAMP3, IL2RB, DNM1L, PFKL, ABHD18, ASIC1, MMP12, KDR, SLC6A8, VEGFA, CD200, CR1L, VLDLR	206	2123	20793	1.806689563	0.08360104	0.01444523	0.53669485
UP_SEQ_FEATURE	glycosylation site:N-linked (GlcNAc...)	35	16.74641148	0.03337879	SCPEP1, IL1R2, CLCN3, ACVRL1, LRRC8A, EFNA1, MMP9, FCNA, HEXB, ACP5, FASLG, HP, GPR1, WNT4, NPTX1, ERO1A, PLOD2, P4HA1, TSPAN31, IL1RAP, SLC2A1, SLC22A5, VWA1, ENTPD2, RAMP3, IL2RB, ABHD18, ASIC1, MMP12, KDR, SLC6A8, VEGFA, CD200, CR1L, VLDLR	108	1887	7976	1.369801174	0.99999893	0.96784841	37.7724721
UP_KEYWORDS	Disulfide bond	30	14.35406699	0.064079226	IL1R2, ACVRL1, EFNA1, MMP9, FCNA, HEXB, ACP5, FASLG, HP, GPR1, WNT4, NPTX1, ERO1A, IL1RAP, VWA1, DNAJC3, ENTPD2, RAMP3, IL2RB, EFEMP2, ASIC1, MMP12, KDR, ADAMTS9, FBLN1, VEGFA, CD200, CR1L, PLAU, VLDLR	206	2199	20793	1.377038106	0.99999864	0.39368917	56.5146439
UP_KEYWORDS	Signal	42	20.09569378	0.101594263	SCPEP1, IL1R2, CPM, ACVRL1, EFNA1, MMP9, FCNA, HEXB, ACP5, CDCP1, HP, WNT4, NPTX1, ERO1A, P4HA2, PLOD2, LRG1, P4HA1, TMEM59, IL1RAP, DNAJC3, VWA1, ENTPD2, RAMP3, IL2RB, TBL2, ABHD18, EFEMP2, MMP12, KDR, CTSM, ADAMTS9, FBLN1, DNAJB9, KAZALD1, VEGFA, VOPP1, SLC27A3, CD200, CR1L, PLAU, VLDLR	206	3420	20793	1.239575881	1	0.46443617	74.0022647
UP_SEQ_FEATURE	signal peptide	27	12.91866029	0.151637191	SCPEP1, IL1R2, ACVRL1, EFNA1, MMP9, FCNA, HEXB, ACP5, HP, NPTX1, WNT4, ERO1A, P4HA1, PLOD2, IL1RAP, VWA1, DNAJC3, RAMP3, IL2RB, ABHD18, MMP12, KDR, VEGFA, CD200, CR1L, PLAU, VLDLR	108	1586	7976	1.257250946	1	0.99938881	89.9526495

UP_SEQ_FEATURE	disulfide bond	21	10.04784689	0.320532301	RAMP3, IL1R2, IL2RB, EFNA1, MMP9, HEXB, FASLG, ACP5, HP, ASIC1, GPR1, MMP12, NPTX1, ERO1A, VEGFA, IL1RAP, VWA1, CD200, PLAU, CR1L, VLDLR	108	1327	7976	1.168718078	1	0.99997059	99.548301
Annotation Cluster 6	Enrichment Score: 1.351022077861093											
Category	Term	Count	%	PValue	Genes	List Total	Pop Hits	Pop Total	Fold Enrichment	Bonferroni	Benjamini	FDR
UP_KEYWORDS	Angiogenesis	5	2.392344498	0.002734207	GPI, ACVRL1, EFNA1, VEGFA, KDR	206	59	20793	8.553974	0.42795761	0.06743626	3.38427263
GOTERM_BP_DIRECT	GO:0045766~positive regulation of angiogenesis	6	2.870813397	0.007165943	ACVRL1, LRG1, MMP9, VEGFA, SPHK1, KDR	181	117	17535	4.968125797	0.9999333	0.49682201	11.1153125
GOTERM_BP_DIRECT	GO:0001938~positive regulation of endothelial cell proliferation	4	1.913875598	0.035285183	ACVRL1, LRG1, VEGFA, KDR	181	70	17535	5.535911602	1	0.69902192	44.4875213
GOTERM_BP_DIRECT	GO:0001525~angiogenesis	6	2.870813397	0.041413516	GPI, ACVRL1, EFNA1, VEGFA, PLAU, KDR	181	184	17535	3.15907999	1	0.72340645	49.9913456
KEGG_PATHWAY	rno04015:Rap1 signaling pathway	4	1.913875598	0.453692122	PARD6B, EFNA1, VEGFA, KDR	90	216	7749	1.594444444	1	0.88945931	99.9285785
GOTERM_BP_DIRECT	GO:0016477~cell migration	3	1.435406699	0.602930261	EFNA1, VEGFA, KDR	181	197	17535	1.475306391	1	0.9999251	99.9999732
Annotation Cluster 7	Enrichment Score: 1.2053612444387278											
Category	Term	Count	%	PValue	Genes	List Total	Pop Hits	Pop Total	Fold Enrichment	Bonferroni	Benjamini	FDR
GOTERM_BP_DIRECT	GO:0051894~positive regulation of focal adhesion assembly	3	1.435406699	0.019361236	WNT4, VEGFA, KDR	181	21	17535	13.83977901	1	0.63409397	27.4088915
KEGG_PATHWAY	rno05205:Proteoglycans in cancer	7	3.349282297	0.027721176	CBLB, WNT4, MMP9, VEGFA, FASLG, PLAU, KDR	90	201	7749	2.998507463	0.98644821	0.20258569	28.5992341
GOTERM_BP_DIRECT	GO:0048754~branching morphogenesis of an epithelial tube	3	1.435406699	0.05257348	WNT4, VEGFA, KDR	181	36	17535	8.07320442	1	0.75056943	58.7219253
UP_KEYWORDS	Developmental protein	6	2.870813397	0.534617989	WNT4, JMJD6, VEGFA, MTURN, CR1L, KDR	206	492	20793	1.230937722	1	0.9126741	99.9933497
Annotation Cluster 8	Enrichment Score: 1.1701569028008831											
Category	Term	Count	%	PValue	Genes	List Total	Pop Hits	Pop Total	Fold Enrichment	Bonferroni	Benjamini	FDR
UP_KEYWORDS	Biological rhythms	4	1.913875598	0.041917288	ID3, BHLHE40, NFIL3, NOCT	206	78	20793	5.176250934	0.99983923	0.36856742	41.6353058
UP_KEYWORDS	Repressor	6	2.870813397	0.057401823	FASLG, HBP1, ID3, BHLHE40, NFIL3, GZF1	206	210	20793	2.883911234	0.99999421	0.37112762	52.4481722
GOTERM_BP_DIRECT	GO:0007623~circadian rhythm	4	1.913875598	0.128295208	ID3, BHLHE40, NFIL3, NOCT	181	121	17535	3.202593489	1	0.91350604	89.4559835
Annotation Cluster 9	Enrichment Score: 1.1464458393870895											
Category	Term	Count	%	PValue	Genes	List Total	Pop Hits	Pop Total	Fold Enrichment	Bonferroni	Benjamini	FDR
GOTERM_BP_DIRECT	GO:0030198~extracellular matrix organization	5	2.392344498	0.017770604	FBLN1, ERO1A, MMP9, KAZALD1, VWA1	181	97	17535	4.993734693	1	0.63170555	25.455479
UP_KEYWORDS	Extracellular matrix	5	2.392344498	0.042834658	FBLN1, WNT4, MMP9, VWA1, MMP12	206	133	20793	3.794620045	0.99986777	0.3601664	42.3341504
GOTERM_CC_DIRECT	GO:0005578~proteinaceous extracellular matrix	7	3.349282297	0.046236352	ADAMTS9, FBLN1, WNT4, MMP9, KAZALD1, VWA1, MMP12	191	253	18520	2.682780457	0.99995823	0.67383943	45.0894641
GOTERM_CC_DIRECT	GO:0031012~extracellular matrix	3	1.435406699	0.737455135	FBLN1, MMP9, MMP12	191	254	18520	1.145236427	1	0.99436708	99.9999956
Annotation Cluster 10	Enrichment Score: 1.1021335054286876											
Category	Term	Count	%	PValue	Genes	List Total	Pop Hits	Pop Total	Fold Enrichment	Bonferroni	Benjamini	FDR

Category	Term	Count	%	PValue	Genes	List Total	Pop Hits	Pop Total	Fold Enrichment	Bonferroni	Benjamini	FDR
GOTERM_BP_DIRECT	GO:0006508~proteolysis	11	5.263157895	0.024118534	CASP6, CBLB, ADAMTS9, TMEM59, MMP9, SCRN1, PREPL, HP, CTLA2A, PLAU, MMP12	181	472	17535	2.257760558	1	0.6281047	32.9682742
UP_KEYWORDS	Protease	8	3.827751196	0.14202623	SCPEP1, CASP6, CPM, ADAMTS9, MMP9, PREPL, PLAU, MMP12	206	437	20793	1.847814979	1	0.56059957	85.4299076
UP_KEYWORDS	Zymogen	4	1.913875598	0.144171701	CASP6, MMP9, PLAU, MMP12	206	133	20793	3.035696036	1	0.55707506	85.8814827

Annotation Cluster 11	Enrichment Score: 1.0511531071161389											
Category	Term	Count	%	PValue	Genes	List Total	Pop Hits	Pop Total	Fold Enrichment	Bonferroni	Benjamini	FDR
GOTERM_BP_DIRECT	GO:0045766~positive regulation of angiogenesis	6	2.870813397	0.007165943	ACVRL1, LRG1, MMP9, VEGFA, SPHK1, KDR	181	117	17535	4.968125797	0.9999333	0.49682201	11.1153125
GOTERM_BP_DIRECT	GO:0001934~positive regulation of protein phosphorylation	5	2.392344498	0.123151047	MMP9, VEGFA, SPHK1, FIZ1, KDR	181	185	17535	2.618336569	1	0.9099127	88.3889021
KEGG_PATHWAY	rno04370:VEGF signaling pathway	3	1.435406699	0.150966015	VEGFA, SPHK1, KDR	90	60	7749	4.305	1	0.57828554	85.9284949
GOTERM_BP_DIRECT	GO:0030335~positive regulation of cell migration	5	2.392344498	0.160489385	VEGFA, SPHK1, ACP5, PLAU, KDR	181	205	17535	2.362889099	1	0.94428615	94.3083713
GOTERM_BP_DIRECT	GO:0008284~positive regulation of cell proliferation	8	3.827751196	0.259535948	CDKN1B, VEGFA, SPHK1, FASLG, HILPDA, ID3, PLAU, KDR	181	503	17535	1.540810386	1	0.98126978	99.2723233

Annotation Cluster 12	Enrichment Score: 1.0461672697393714											
Category	Term	Count	%	PValue	Genes	List Total	Pop Hits	Pop Total	Fold Enrichment	Bonferroni	Benjamini	FDR
GOTERM_BP_DIRECT	GO:0008203~cholesterol metabolic process	4	1.913875598	0.025858274	INSIG2, SQLE, INSIG1, VLDLR	181	62	17535	6.250222777	1	0.62204582	34.8995547
UP_KEYWORDS	Cholesterol metabolism	3	1.435406699	0.06440183	INSIG2, INSIG1, VLDLR	206	42	20793	7.209778086	0.99999873	0.38430537	56.7027492
UP_KEYWORDS	Sterol metabolism	3	1.435406699	0.081240553	INSIG2, INSIG1, VLDLR	206	48	20793	6.308555825	0.99999997	0.40772919	65.5429906
UP_KEYWORDS	Steroid metabolism	3	1.435406699	0.121508809	INSIG2, INSIG1, VLDLR	206	61	20793	4.964109502	1	0.5104518	80.3880644
UP_KEYWORDS	Lipid metabolism	5	2.392344498	0.357507597	SLC27A1, INSIG2, INSIG1, CHPT1, VLDLR	206	306	20793	1.649295641	1	0.79470911	99.6162778

Annotation Cluster 13	Enrichment Score: 0.9831099722786809											
Category	Term	Count	%	PValue	Genes	List Total	Pop Hits	Pop Total	Fold Enrichment	Bonferroni	Benjamini	FDR
INTERPRO	IPR013026:Tetratricopeptide repeat-containing domain	5	2.392344498	0.032953946	P4HA2, P4HA1, ZC3H7B, KLC4, DNAJC3	199	118	19384	4.127416745	0.99999986	0.95742818	38.0281944
INTERPRO	IPR019734:Tetratricopeptide repeat	5	2.392344498	0.036570229	P4HA2, P4HA1, ZC3H7B, KLC4, DNAJC3	199	122	19384	3.992091606	0.99999998	0.94631229	41.2564468
INTERPRO	IPR011990:Tetratricopeptide-like helical	5	2.392344498	0.149176857	P4HA2, P4HA1, ZC3H7B, KLC4, DNAJC3	199	200	19384	2.435175879	1	0.99900952	90.0425693
UP_KEYWORDS	TPR repeat	3	1.435406699	0.179538342	P4HA1, KLC4, DNAJC3	206	78	20793	3.8821882	1	0.61755513	91.6954253
SMART	SM00028:TPR	3	1.435406699	0.376319189	ZC3H7B, KLC4, DNAJC3	109	117	9680	2.277111268	1	0.99747154	99.5342192

Annotation Cluster 14	Enrichment Score: 0.796253214927415											
Category	Term	Count	%	PValue	Genes	List Total	Pop Hits	Pop Total	Fold Enrichment	Bonferroni	Benjamini	FDR
INTERPRO	IPR001623:DnaJ domain	3	1.435406699	0.095653732	DNAJB9, DNAJB1, DNAJC3	199	51	19384	5.729825599	1	0.99731337	76.2049574
SMART	SM00271:DnaJ	3	1.435406699	0.099969472	DNAJB9, DNAJB1, DNAJC3	109	48	9680	5.550458716	0.9999939	0.95030286	69.8154848
COG_ONTOLOGY	Posttranslational modification, protein turnover, chaperones	3	1.435406699	0.427239148	DNAJB9, DNAJB1, DNAJC3	22	119	1738	1.991596639	0.99875354	0.99875354	97.2274504

Annotation Cluster 15	Enrichment Score: 0.7895876289108528											
Category	Term	Count	%	PValue	Genes	List Total	Pop Hits	Pop Total	Fold Enrichment	Bonferroni	Benjamini	FDR

UP_KEYWORDS	Transcription	15	7.177033493	0.052972451	MAFF, LRIF1, FASLG, NR4A3, FOXO3, IL33, FOXO4, GZF1, JMJD6, KDM3A, HBP1, BHLHE40, VOPP1, ID3, NFIL3	206	878	20793	1.724434394	0.99998493	0.38291264	49.5606693
UP_KEYWORDS	Repressor	6	2.870813397	0.057401823	FASLG, HBP1, ID3, BHLHE40, NFIL3, GZF1	206	210	20793	2.883911234	0.99999421	0.37112762	52.4481722
UP_KEYWORDS	Transcription regulation	12	5.741626794	0.196816319	MAFF, LRIF1, JMJD6, FASLG, KDM3A, HBP1, ID3, NR4A3, BHLHE40, VOPP1, NFIL3, GZF1	206	829	20793	1.461088925	1	0.63801785	93.6454448
GOTERM_BP_DIRECT	GO:0006351~transcription, DNA-templated	11	5.263157895	0.2911622	LRIF1, JMJD6, FASLG, KDM3A, HBP1, ID3, BHLHE40, VOPP1, IL33, FOXO4, GZF1	181	788	17535	1.352364192	1	0.98697083	99.6440877
GOTERM_BP_DIRECT	GO:0006355~regulation of transcription, DNA-templated	11	5.263157895	0.646978733	PRKCQ, ACVRL1, LRIF1, JMJD6, MNT, KDM3A, HBP1, VOPP1, FOXO3, FOXO4, MLLT3	181	1054	17535	1.011065449	1	0.99997371	99.9999961

Annotation Cluster 16 Enrichment Score: 0.7736176964794167

Category	Term	Count	%	PValue	Genes	List Total	Pop Hits	Pop Total	Fold Enrichment	Bonferroni	Benjamini	FDR
GOTERM_CC_DIRECT	GO:0005578~proteinaceous extracellular matrix	7	3.349282297	0.046236352	ADAMTS9, FBLN1, WNT4, MMP9, KAZALD1, VWA1, MMP12	191	253	18520	2.682780457	0.99995823	0.67383943	45.0894641
UP_KEYWORDS	Protease	8	3.827751196	0.14202623	SCPEP1, CASP6, CPM, ADAMTS9, MMP9, PREPL, PLAU, MMP12	206	437	20793	1.847814979	1	0.56059957	85.4299076
INTERPRO	IPR024079:Metallopeptidase, catalytic domain	3	1.435406699	0.234357803	ADAMTS9, MMP9, MMP12	199	90	19384	3.246901173	1	0.99977175	97.7922038
UP_KEYWORDS	Metalloprotease	3	1.435406699	0.259006526	ADAMTS9, MMP9, MMP12	206	100	20793	3.028106796	1	0.69852068	97.6934229
GOTERM_MF_DIRECT	GO:0004222~metalloendopeptidase activity	3	1.435406699	0.339914173	ADAMTS9, MMP9, MMP12	174	116	16650	2.474732461	1	0.98988437	99.6790468

Annotation Cluster 17 Enrichment Score: 0.6180740779887289

Category	Term	Count	%	PValue	Genes	List Total	Pop Hits	Pop Total	Fold Enrichment	Bonferroni	Benjamini	FDR
INTERPRO	IPR000198:Rho GTPase-activating protein domain	3	1.435406699	0.178473156	DLC1, BCR, ARHGAP8	199	75	19384	3.896281407	1	0.99865837	93.9625989
SMART	SM00324:RhoGAP	3	1.435406699	0.195762435	DLC1, BCR, ARHGAP8	109	73	9680	3.64961669	1	0.98406681	91.605968
INTERPRO	IPR008936:Rho GTPase activation protein	3	1.435406699	0.264610022	DLC1, BCR, ARHGAP8	199	98	19384	2.981848016	1	0.99967848	98.7584818
GOTERM_MF_DIRECT	GO:0005096~GTPase activator activity	4	1.913875598	0.364581928	DLC1, BCR, ACAP1, ARHGAP8	174	207	16650	1.849075462	1	0.99003082	99.8104136

Annotation Cluster 18 Enrichment Score: 0.49441805344876066

Category	Term	Count	%	PValue	Genes	List Total	Pop Hits	Pop Total	Fold Enrichment	Bonferroni	Benjamini	FDR
INTERPRO	IPR009030:Insulin-like growth factor binding protein, N-terminal	4	1.913875598	0.143243958	FBLN1, EFEMP2, KAZALD1, VLDLR	199	128	19384	3.043969849	1	0.99931202	89.0038457
UP_KEYWORDS	EGF-like domain	4	1.913875598	0.232790067	FBLN1, EFEMP2, PLAU, VLDLR	206	169	20793	2.389038892	1	0.67574594	96.4285684
INTERPRO	IPR018097:EGF-like calcium-binding, conserved site	3	1.435406699	0.249469222	FBLN1, EFEMP2, VLDLR	199	94	19384	3.108735165	1	0.99978563	98.3391241
INTERPRO	IPR000152:EGF-type aspartate/asparagine hydroxylation site	3	1.435406699	0.253252747	FBLN1, EFEMP2, VLDLR	199	95	19384	3.076011637	1	0.99969369	98.4547608
INTERPRO	IPR013032:EGF-like, conserved site	4	1.913875598	0.304371264	FBLN1, EFEMP2, PLAU, VLDLR	199	189	19384	2.061524554	1	0.99980592	99.4386346
INTERPRO	IPR001881:EGF-like calcium-binding	3	1.435406699	0.351020098	FBLN1, EFEMP2, VLDLR	199	121	19384	2.415050459	1	0.99970997	99.7916588
INTERPRO	IPR000742:Epidermal growth factor-like domain	4	1.913875598	0.382246513	FBLN1, EFEMP2, PLAU, VLDLR	199	217	19384	1.795521386	1	0.99983762	99.8969693
SMART	SM00179:EGF_CA	3	1.435406699	0.392220733	FBLN1, EFEMP2, VLDLR	109	121	9680	2.201834862	1	0.99657467	99.6527675
SMART	SM00181:EGF	3	1.435406699	0.572196045	FBLN1, EFEMP2, VLDLR	109	171	9680	1.558023499	1	0.99941615	99.9935985

GOTERM_MF_DIRECT	GO:0005509~calcium ion binding	8	3.827751196	0.588915531	GUCA1A, CBLB, FBLN1, EFEMP2, ACTN1, DST, MMP12, VLDLR	174	696	16650	1.099881094	1	0.99918445	99.9995391
Annotation Cluster 19	Enrichment Score: 0.4724266150688843											
Category	Term	Count	%	PValue	Genes	List Total	Pop Hits	Pop Total	Fold Enrichment	Bonferroni	Benjamini	FDR
SMART	SM00184:RING	5	2.392344498	0.211808037	RNF125, CBLB, MEX3B, RNF138, HLTF	109	212	9680	2.094512723	1	0.979271	93.3252763
UP_KEYWORDS	Zinc-finger	12	5.741626794	0.217884813	RNF125, PRKCQ, CBLB, MEX3B, ZMYND10, RYBP, KDM4B, RNF138, KDM3A, NR4A3, DTNA, GZF1	206	849	20793	1.426669869	1	0.65584863	95.4509364
UP_KEYWORDS	Zinc	16	7.655502392	0.247389427	ZMYND10, MMP9, MEX3B, RYBP, NR4A3, MMP12, GZF1, RNF125, HAGH, PRKCQ, CBLB, ZFP185, KDM4B, RNF138, KDM3A, DTNA	206	1245	20793	1.297180957	1	0.69371236	97.1950759
UP_KEYWORDS	Ligase	4	1.913875598	0.280558241	SLC27A1, CBLB, WWP1, RNF138	206	187	20793	2.159077929	1	0.71844183	98.408607
INTERPRO	IPR001841:Zinc finger, RING-type	5	2.392344498	0.281419186	RNF125, CBLB, MEX3B, RNF138, HLTF	199	261	19384	1.866035157	1	0.99972324	99.107603
GOTERM_MF_DIRECT	GO:0008270~zinc ion binding	14	6.698564593	0.301518326	RNF125, CBLB, CPM, ADAMTS9, MMP9, MEX3B, RYBP, KDM4B, ZFP185, RNF138, NR4A3, HLTF, MMP12, DTNA	174	1054	16650	1.271020088	1	0.98712488	99.2987938
GOTERM_MF_DIRECT	GO:0061630~ubiquitin protein ligase activity	4	1.913875598	0.339126918	CBLB, WWP1, RNF138, HLTF	174	198	16650	1.933124347	1	0.99107854	99.6737151
GOTERM_MF_DIRECT	GO:0016874~ligase activity	3	1.435406699	0.347472615	CBLB, WWP1, RNF138	174	118	16650	2.432787843	1	0.98842324	99.72628
INTERPRO	IPR013083:Zinc finger, RING/FYVE/PHD-type	6	2.870813397	0.412625749	RNF125, CBLB, MEX3B, KDM4B, RNF138, HLTF	199	414	19384	1.411696162	1	0.99969168	99.9498531
INTERPRO	IPR017907:Zinc finger, RING-type, conserved site	3	1.435406699	0.437323852	RNF125, CBLB, HLTF	199	145	19384	2.015317969	1	0.99978908	99.9728455
GOTERM_BP_DIRECT	GO:0016567~protein ubiquitination	5	2.392344498	0.483009787	RNF125, CBLB, SPSB1, WWP1, RNF138	181	349	17535	1.387943453	1	0.99908856	99.9979786
UP_KEYWORDS	Ubl conjugation pathway	3	1.435406699	0.767434603	CBLB, WWP1, RNF138	206	281	20793	1.077618077	1	0.98206428	99.9999989
Annotation Cluster 20	Enrichment Score: 0.4686337683903179											
Category	Term	Count	%	PValue	Genes	List Total	Pop Hits	Pop Total	Fold Enrichment	Bonferroni	Benjamini	FDR
UP_KEYWORDS	Sodium transport	3	1.435406699	0.251702027	SLC6A8, ASIC1, SLC22A5	206	98	20793	3.089904894	1	0.69364552	97.3906154
UP_KEYWORDS	Sodium	3	1.435406699	0.26996763	SLC6A8, ASIC1, SLC22A5	206	103	20793	2.939909511	1	0.70900829	98.0875864
UP_KEYWORDS	Ion transport	6	2.870813397	0.577963334	CLCN3, LRRC8A, SLC6A8, ASIC1, SLC22A5, KCTD11	206	516	20793	1.173684805	1	0.93049893	99.998055
Annotation Cluster 21	Enrichment Score: 0.454903740797684											
Category	Term	Count	%	PValue	Genes	List Total	Pop Hits	Pop Total	Fold Enrichment	Bonferroni	Benjamini	FDR
INTERPRO	IPR013151:Immunoglobulin	3	1.435406699	0.098836821	IL1R2, CD200, KDR	199	52	19384	5.619636645	1	0.99568766	77.3733498
UP_SEQ_FEATURE	domain:Ig-like C2-type 3	3	1.435406699	0.158593522	IL1R2, IL1RAP, KDR	108	53	7976	4.180293501	1	0.99908222	91.044572
UP_SEQ_FEATURE	domain:Ig-like C2-type 2	3	1.435406699	0.261503815	IL1R2, IL1RAP, KDR	108	74	7976	2.993993994	1	0.99984458	98.5532471
UP_SEQ_FEATURE	domain:Ig-like C2-type 1	3	1.435406699	0.261503815	IL1R2, IL1RAP, KDR	108	74	7976	2.993993994	1	0.99984458	98.5532471
INTERPRO	IPR013783:Immunoglobulin-like fold	10	4.784688995	0.340247449	IL1R2, IL2RB, GBE1, KAZALD1, IL1RAP, VSIR, STBD1, VWA1, CD200, KDR	199	737	19384	1.321669405	1	0.99971467	99.7364456

INTERPRO	IPR003599:Immunoglobulin subtype	6	2.870813397	0.388189596	IL1R2, KAZALD1, IL1RAP, VSIR, CD200, KDR	199	401	19384	1.457461873	1	0.99981048	99.9102536
UP_KEYWORDS	Immunoglobulin domain	4	1.913875598	0.46071788	IL1R2, IL1RAP, CD200, KDR	206	255	20793	1.583323815	1	0.88177215	99.957568
SMART	SM00409:IG	6	2.870813397	0.465321644	IL1R2, KAZALD1, IL1RAP, VSIR, CD200, KDR	109	401	9680	1.328788121	1	0.99847928	99.919147
INTERPRO	IPR003598:Immunoglobulin subtype 2	3	1.435406699	0.672030763	IL1R2, KAZALD1, KDR	199	225	19384	1.298760469	1	0.99999801	99.9999878
INTERPRO	IPR007110:Immunoglobulin-like domain	6	2.870813397	0.674624314	IL1R2, KAZALD1, IL1RAP, VSIR, CD200, KDR	199	556	19384	1.051155056	1	0.9999966	99.9999891
SMART	SM00408:IGc2	3	1.435406699	0.720145663	IL1R2, KAZALD1, KDR	109	225	9680	1.184097859	1	0.99988535	99.9999487

Annotation Cluster 22 Enrichment Score: 0.4145510870125015

Category	Term	Count	%	PValue	Genes	List Total	Pop Hits	Pop Total	Fold Enrichment	Bonferroni	Benjamini	FDR
INTERPRO	IPR011598:Myc-type, basic helix-loop-helix (bHLH) domain	3	1.435406699	0.317467261	MNT, ID3, BHLHE40	199	112	19384	2.609117014	1	0.99971898	99.5720598
SMART	SM00353:HLH	3	1.435406699	0.344043389	MNT, ID3, BHLHE40	109	109	9680	2.4442387	1	0.99754263	99.1732253
GOTERM_MF_DIRECT	GO:0046983~protein dimerization activity	3	1.435406699	0.522437782	MNT, ID3, BHLHE40	174	170	16650	1.688640974	1	0.99886114	99.9963405

Annotation Cluster 23 Enrichment Score: 0.3764170409833939

Category	Term	Count	%	PValue	Genes	List Total	Pop Hits	Pop Total	Fold Enrichment	Bonferroni	Benjamini	FDR
INTERPRO	IPR001245:Serine-threonine/tyrosine-protein kinase catalytic domain	4	1.913875598	0.162607411	ACVRL1, ZAP70, KSR1, KDR	199	136	19384	2.864912799	1	0.99905601	92.0663315
GOTERM_BP_DIRECT	GO:0006468~protein phosphorylation	8	3.827751196	0.256404461	PDK1, PRKCQ, ACVRL1, PRKAG2, MAP3K8, ZAP70, KSR1, EIF2AK4	181	501	17535	1.546961326	1	0.98095536	99.2202287
GOTERM_BP_DIRECT	GO:0046777~protein autophosphorylation	4	1.913875598	0.297473384	BCR, ZAP70, EIF2AK4, KDR	181	186	17535	2.083407592	1	0.98736015	99.6925987
KEGG_PATHWAY	rno04660:T cell receptor signaling pathway	3	1.435406699	0.340432943	PRKCQ, MAP3K8, ZAP70	90	105	7749	2.46	1	0.82109958	99.3172529
UP_SEQ_FEATURE	binding site:ATP	6	2.870813397	0.341105313	PDK1, PRKCQ, ACVRL1, MAP3K8, PGK1, KDR	108	287	7976	1.543941154	1	0.99995174	99.7060546
INTERPRO	IPR000719:Protein kinase, catalytic domain	7	3.349282297	0.401159962	PRKCQ, ACVRL1, MAP3K8, ZAP70, KSR1, EIF2AK4, KDR	199	499	19384	1.366431355	1	0.99982051	99.9339105
INTERPRO	IPR008271:Serine/threonine-protein kinase, active site	5	2.392344498	0.411628878	PRKCQ, ACVRL1, MAP3K8, KSR1, EIF2AK4	199	319	19384	1.526756037	1	0.99975816	99.9486241
INTERPRO	IPR011009:Protein kinase-like domain	7	3.349282297	0.466607829	PRKCQ, ACVRL1, MAP3K8, ZAP70, KSR1, EIF2AK4, KDR	199	540	19384	1.262683789	1	0.9998345	99.9873413
UP_KEYWORDS	Serine/threonine-protein kinase	4	1.913875598	0.498248343	PRKCQ, ACVRL1, MAP3K8, EIF2AK4	206	270	20793	1.495361381	1	0.90037782	99.9828696
INTERPRO	IPR017441:Protein kinase, ATP binding site	5	2.392344498	0.542792219	PRKCQ, ACVRL1, ZAP70, EIF2AK4, KDR	199	379	19384	1.285053235	1	0.99993874	99.9985984
GOTERM_MF_DIRECT	GO:0004672~protein kinase activity	4	1.913875598	0.555231528	PDK1, MAP3K8, ZAP70, KSR1	174	278	16650	1.376829571	1	0.99885194	99.9986311
UP_SEQ_FEATURE	active site:Proton acceptor	6	2.870813397	0.570406022	PRKCQ, ACVRL1, PFKL, ALDOC, MAP3K8, KDR	108	375	7976	1.18162963	1	0.99999998	99.9992542
SMART	SM00220:S_TKc	5	2.392344498	0.605055764	PRKCQ, ACVRL1, MAP3K8, KSR1, EIF2AK4	109	374	9680	1.187263896	1	0.99914164	99.9974204
UP_SEQ_FEATURE	domain:Protein kinase	4	1.913875598	0.612501547	PRKCQ, ACVRL1, MAP3K8, KDR	108	234	7976	1.262424818	1	1	99.9998235
GOTERM_MF_DIRECT	GO:0004674~protein serine/threonine kinase activity	4	1.913875598	0.641053929	PDK1, PRKCQ, ACVRL1, EIF2AK4	174	316	16650	1.211261458	1	0.99947588	99.9999293

Annotation Cluster 24 Enrichment Score: 0.3375024849885026

Category	Term	Count	%	PValue	Genes	List Total	Pop Hits	Pop Total	Fold Enrichment	Bonferroni	Benjamini	FDR

UP_SEQ_FEATURE	topological domain:Extracellular	22	10.52631579	0.116688592	RAMP3, IL1R2, SLC27A1, IL2RB, ACVRL1, LRRC8A, FASLG, ASIC1, GPR1, KDR, SLC16A3, SLC6A8, TSPAN31, SLC2A1, IL1RAP, SLC22A5, STBD1, ENTPD2, CD200, CR1L, HIGD1A, VLDLR	108	1199	7976	1.35507985	1	0.99812926	82.3382388
UP_SEQ_FEATURE	transmembrane region	34	16.26794258	0.218243631	SLC27A1, IL1R2, TSPO, CLCN3, ACVRL1, LRRC8A, FASLG, COMT, GPR1, INSIG2, TSPAN31, INSIG1, IL1RAP, SLC2A1, SEC22B, SLC22A5, ENTPD2, HIGD1A, RAMP3, IL2RB, ASIC1, CHPT1, KDR, SLC16A3, PARP16, SQLE, SLC6A8, SLC35E1, SURF4, LOC691807, STBD1, CD200, CR1L, VLDLR	108	2160	7976	1.162482853	1	0.99988437	96.7947792
UP_SEQ_FEATURE	topological domain:Cytoplasmic	25	11.96172249	0.239009014	RAMP3, SLC27A1, IL1R2, IL2RB, CLCN3, ACVRL1, LRRC8A, FASLG, ASIC1, GPR1, KDR, SLC16A3, SLC6A8, TSPAN31, SLC2A1, IL1RAP, INSIG1, SEC22B, SLC22A5, STBD1, CD200, ENTPD2, CR1L, HIGD1A, VLDLR	108	1541	7976	1.198115702	1	0.99990079	97.7996912
GOTERM_CC_DIRECT	GO:0005887~integral component of plasma membrane	12	5.741626794	0.373631849	SLC16A3, ACVRL1, TSPAN33, SLC6A8, TSPAN31, SLC2A1, ASIC1, SLC22A5, PLPP1, GPR1, CD200, KDR	191	942	18520	1.235201921	1	0.94664224	99.7325596
UP_KEYWORDS	Membrane	64	30.62200957	0.914463911	DLC1, SLC27A1, TSPO, ACVRL1, LRRC8A, EFNA1, BNIP3, FASLG, CDCP1, INSIG2, PLOD2, TMEM59, SLC2A1, IL1RAP, INSIG1, PHTF1, GNG2, SLC22A5, HIGD1A, RAMP3, ACTN1, BASP1, ARRDC3, CHPT1, PARP16, PRKCQ, SQLE, SLC25A36, VSIR, SLC35E1, SURF4, VOPP1, STBD1, RASD1, CR1L, IL1R2, CLCN3, FCNA, COMT, PLPP1, GPR1, FAM65B, TOMM7, TSPAN33, ERO1A, TSPAN31, SEC22B, NDRG1, ENTPD4, ENTPD2, IL2RB, DNM1L, SPHK1, XKR8, ATP11B, HILPDA, ASIC1, KDR, SLC16A3, SLC6A8, BNIP3L, LOC691807, CD200, VLDLR	206	7278	20793	0.887601149	1	0.99779506	100

UP_KEYWORDS	Transmembrane helix	49	23.44497608	0.982304201	SLC27A1, IL1R2, TSPO, CLCN3, ACVRL1, LRRC8A, BNIP3, FASLG, CDCP1, COMT, PLPP1, GPR1, TOMM7, TSPAN33, INSIG2, TMEM59, TSPAN31, SLC2A1, IL1RAP, INSIG1, PHTF1, SEC22B, SLC22A5, ENTPD4, ENTPD2, HIGD1A, RAMP3, IL2RB, XKR8, ATP11B, HILPDA, ASIC1, CHPT1, KDR, SLC16A3, PARP16, SQLE, SLC6A8, SLC25A36, BNIP3L, VSIR, SLC35E1, SURF4, VOPP1, LOC691807, STBD1, CD200, CR1L, VLDLR	206	6182	20793	0.800049784	1	0.99995062	100
UP_KEYWORDS	Transmembrane	49	23.44497608	0.983914828	SLC27A1, IL1R2, TSPO, CLCN3, ACVRL1, LRRC8A, BNIP3, FASLG, CDCP1, COMT, PLPP1, GPR1, TOMM7, TSPAN33, INSIG2, TMEM59, TSPAN31, SLC2A1, IL1RAP, INSIG1, PHTF1, SEC22B, SLC22A5, ENTPD4, ENTPD2, HIGD1A, RAMP3, IL2RB, XKR8, ATP11B, HILPDA, ASIC1, CHPT1, KDR, SLC16A3, PARP16, SQLE, SLC6A8, SLC25A36, BNIP3L, VSIR, SLC35E1, SURF4, VOPP1, LOC691807, STBD1, CD200, CR1L, VLDLR	206	6208	20793	0.79669906	1	0.99995593	100
GOTERM_CC_DIRECT	GO:0016021~integral component of membrane	45	21.53110048	0.992617426	SLC27A1, IL1R2, TSPO, CLCN3, BNIP3, ACP5, FASLG, CDCP1, COMT, PLPP1, GPR1, TMEM59, SLC2A1, IL1RAP, INSIG1, PHTF1, SEC22B, SLC22A5, ENTPD4, ENTPD2, HIGD1A, RAMP3, IL2RB, XKR8, ATP11B, HILPDA, ASIC1, CHPT1, KDR, PARP16, IFI27, SYNE1, SQLE, SLC25A36, IFI27L2B, BNIP3L, VSIR, SLC35E1, SURF4, LOC691807, SLC27A3, STBD1, CD200, CR1L, VLDLR	191	5711	18520	0.764025702	1	0.99999996	100
Annotation Cluster 25	Enrichment Score: 0.31897798257692345											
Category	Term	Count	%	PValue	Genes	List Total	Pop Hits	Pop Total	Fold Enrichment	Bonferroni	Benjamini	FDR
INTERPRO	IPR011333:BTB/POZ fold	4	1.913875598	0.323411271	RCBTB1, BCL6, KCTD11, GZF1	199	194	19384	2.008392478	1	0.99966477	99.6223065
INTERPRO	IPR000210:BTB/POZ-like	3	1.435406699	0.565927722	RCBTB1, BCL6, GZF1	199	185	19384	1.579573543	1	0.99995804	99.9993323
SMART	SM00225:BTB	3	1.435406699	0.603323071	RCBTB1, BCL6, GZF1	109	181	9680	1.471944853	1	0.99946281	99.9972888
Annotation Cluster 26	Enrichment Score: 0.2957409272924174											
Category	Term	Count	%	PValue	Genes	List Total	Pop Hits	Pop Total	Fold Enrichment	Bonferroni	Benjamini	FDR

INTERPRO	IPR011992:EF-hand-like domain	5	2.392344498	0.314029506	GUCA1A, CBLB, ACTN1, DST, DTNA	199	277	19384	1.758249732	1	0.99978691	99.5402307
SMART	SM00054:EFh	3	1.435406699	0.490601774	GUCA1A, ACTN1, DST	109	147	9680	1.812394683	1	0.99835166	99.9533909
INTERPRO	IPR018247:EF-Hand 1, calcium-binding site	3	1.435406699	0.542784762	GUCA1A, ACTN1, DST	199	178	19384	1.641691604	1	0.99995286	99.9985981
GOTERM_MF_DIRECT	GO:0005509~calcium ion binding	8	3.827751196	0.588915531	GUCA1A, CBLB, FBLN1, EFEMP2, ACTN1, DST, MMP12, VLDLR	174	696	16650	1.099881094	1	0.99918445	99.9995391
INTERPRO	IPR002048:EF-hand domain	3	1.435406699	0.674396238	GUCA1A, ACTN1, DST	199	226	19384	1.293013741	1	0.99999748	99.999989

Annotation Cluster 27 Enrichment Score: 0.29468093984559085

Category	Term	Count	%	PValue	Genes	List Total	Pop Hits	Pop Total	Fold Enrichment	Bonferroni	Benjamini	FDR
GOTERM_CC_DIRECT	GO:0005874~microtubule	4	1.913875598	0.429548315	TPGS2, DNM1L, KLC4, NDRG1	191	236	18520	1.643446623	1	0.95699266	99.9181597
UP_KEYWORDS	Microtubule	3	1.435406699	0.487898345	TPGS2, KLC4, NDRG1	206	166	20793	1.824160721	1	0.8972431	99.9778553
UP_KEYWORDS	Cytoskeleton	6	2.870813397	0.623183313	TPGS2, ZMYND10, MLLT11, KLC4, ACTN1, NDRG1	206	543	20793	1.115324787	1	0.94649549	99.9995323

Annotation Cluster 28 Enrichment Score: 0.27721793909155346

Category	Term	Count	%	PValue	Genes	List Total	Pop Hits	Pop Total	Fold Enrichment	Bonferroni	Benjamini	FDR
GOTERM_BP_DIRECT	GO:0006366~transcription from RNA polymerase II promoter	6	2.870813397	0.388117253	MAFF, BCL11A, NR4A3, FOXO3, NFIL3, HLTF	181	398	17535	1.460479192	1	0.99560689	99.9680296
GOTERM_MF_DIRECT	GO:0043565~sequence-specific DNA binding	7	3.349282297	0.541721961	MAFF, BCL11A, BCL6, NR4A3, FOXO3, FOXO4, GZF1	174	568	16650	1.179273919	1	0.99891145	99.9979299
GOTERM_MF_DIRECT	GO:0000978~RNA polymerase II core promoter proximal region sequence-specific DNA binding	4	1.913875598	0.700820492	BCL11A, NR4A3, HLTF, GZF1	174	346	16650	1.106238788	1	0.99965087	99.9999943

Annotation Cluster 29 Enrichment Score: 0.22230589872411322

Category	Term	Count	%	PValue	Genes	List Total	Pop Hits	Pop Total	Fold Enrichment	Bonferroni	Benjamini	FDR
GOTERM_CC_DIRECT	GO:0005768~endosome	4	1.913875598	0.475222159	CLCN3, VOPP1, ARRDC3, KDR	191	250	18520	1.551413613	1	0.96199194	99.9715546
GOTERM_CC_DIRECT	GO:0005769~early endosome	3	1.435406699	0.644987557	CLCN3, ARRDC3, KDR	191	213	18520	1.365680997	1	0.98676861	99.9997983
UP_KEYWORDS	Endosome	3	1.435406699	0.702480518	CLCN3, ARRDC3, KDR	206	247	20793	1.225954168	1	0.96928972	99.999976

Annotation Cluster 30 Enrichment Score: 0.22170715301208152

Category	Term	Count	%	PValue	Genes	List Total	Pop Hits	Pop Total	Fold Enrichment	Bonferroni	Benjamini	FDR
GOTERM_CC_DIRECT	GO:0005913~cell-cell adherens junction	4	1.913875598	0.504432893	PLIN3, NDRG1, DNAJB1, CD200	191	259	18520	1.497503487	1	0.96395721	99.9862266
GOTERM_BP_DIRECT	GO:0098609~cell-cell adhesion	3	1.435406699	0.640291525	PLIN3, NDRG1, DNAJB1	181	211	17535	1.377418764	1	0.99997062	99.9999947
GOTERM_MF_DIRECT	GO:0098641~cadherin binding involved in cell-cell adhesion	3	1.435406699	0.669418005	PLIN3, NDRG1, DNAJB1	174	220	16650	1.304858934	1	0.99948285	99.9999773

Annotation Cluster 31 Enrichment Score: 0.13899474661521488

Category	Term	Count	%	PValue	Genes	List Total	Pop Hits	Pop Total	Fold Enrichment	Bonferroni	Benjamini	FDR
INTERPRO	IPR019775:WD40 repeat, conserved site	3	1.435406699	0.507499205	TBL2, TEP1, DENND3	199	166	19384	1.760368106	1	0.99990545	99.9959472
INTERPRO	IPR001680:WD40 repeat	3	1.435406699	0.746867134	TBL2, TEP1, DENND3	199	260	19384	1.123927329	1	0.99999959	99.9999997
SMART	SM00320:WD40	3	1.435406699	0.789541377	TBL2, TEP1, DENND3	109	259	9680	1.028656442	1	0.99994831	99.999998
INTERPRO	IPR017986:WD40-repeat-containing domain	3	1.435406699	0.800493048	TBL2, TEP1, DENND3	199	291	19384	1.004196239	1	0.99999993	100
INTERPRO	IPR015943:WD40/YVTN repeat-like-containing domain	3	1.435406699	0.842587657	TBL2, TEP1, DENND3	199	321	19384	0.910346123	1	0.99999999	100

Annotation Cluster 32 Enrichment Score: 0.1179127363824024

Category	Term	Count	%	PValue	Genes	List Total	Pop Hits	Pop Total	Fold Enrichment	Bonferroni	Benjamini	FDR
UP_KEYWORDS	GTP-binding	3	1.435406699	0.716973931	DNM1L, AK4, RASD1	206	254	20793	1.19216803	1	0.9720204	99.9999872

GOTERM_MF_DIRECT	GO:0005525~GTP binding	4	1.913875598	0.759125289	DNM1L, ARL16, AK4, RASD1	174	380	16650	1.007259528	1	0.99988512	99.9999997
INTERPRO	IPR027417:P-loop containing nucleoside triphosphate hydrolase	8	3.827751196	0.813663747	DNM1L, PFKFB3, DDX50, TEP1, ARL16, AK4, HLTF, RASD1	199	895	19384	0.870677409	1	0.99999995	100

Annotation Cluster 33 Enrichment Score: 0.0949871929647967

Category	Term	Count	%	PValue	Genes	List Total	Pop Hits	Pop Total	Fold Enrichment	Bonferroni	Benjamini	FDR
INTERPRO	IPR001849:Pleckstrin homology domain	3	1.435406699	0.73714815	BCR, ACAP1, APPL1	199	255	19384	1.14596512	1	0.99999956	99.9999995
SMART	SM00233:PH	3	1.435406699	0.762997137	BCR, ACAP1, APPL1	109	245	9680	1.08743681	1	0.99993585	99.9999923
INTERPRO	IPR011993:Pleckstrin homology-like domain	3	1.435406699	0.922488015	BCR, ACAP1, APPL1	199	407	19384	0.717987974	1	1	100

Annotation Cluster 34 Enrichment Score: 0.06285416103948721

Category	Term	Count	%	PValue	Genes	List Total	Pop Hits	Pop Total	Fold Enrichment	Bonferroni	Benjamini	FDR
INTERPRO	IPR013087:Zinc finger C2H2-type/integrase DNA-binding domain	5	2.392344498	0.74927517	BCL11A, BCL6, ZFP503, FIZ1, GZF1	199	496	19384	0.981925758	1	0.99999949	99.9999997
INTERPRO	IPR007087:Zinc finger, C2H2	5	2.392344498	0.852975947	BCL11A, BCL6, ZFP503, FIZ1, GZF1	199	586	19384	0.831118048	1	0.99999999	100
INTERPRO	IPR015880:Zinc finger, C2H2-like	4	1.913875598	0.923215762	BCL11A, BCL6, FIZ1, GZF1	199	551	19384	0.707129112	1	1	100
SMART	SM00355:ZnF_C2H2	4	1.913875598	0.949952784	BCL11A, BCL6, FIZ1, GZF1	109	551	9680	0.644699379	1	0.99999998	100
