# Supplementary material for: Predicted rat interactome database and gene set linkage analysis
Source: Database (Oxford). 2020 Nov 20;2020:baaa086. doi: 10.1093/database/baaa086 (PMC7678787; doi:10.1093/database/baaa086)
Supplement: baaa086_Supp [file baaa086_supp.zip › Table_S8.pdf]

Supplementary Table 8. Annotations reported by gene ontology analysis for the top 250 transcriptionally changed genes.

Analysis Type:	PANTHER Overrepresentation Test (Released 20190711)						
Annotation Version and Release Date:	GO Ontology database Released 2019-07-03						
Analyzed List:	upload_1 (Rattus norvegicus)						
Reference List:	Rattus norvegicus (all genes in database)						
Test Type:	FISHER						
Correction:	FDR						
GO biological process complete	Rattus norvegicus - REFLIST (21465)	upload_1 (204)	upload_1 (expected)	upload_1 (over/under)	upload_1 (fold Enrichment)	upload_1 (raw P-value)	upload_1 (FDR)
peptidyl-proline hydroxylation to 4-hydroxy-L-proline (GO:0018401)	8	4	0.08	+	52.61	3.56E-06	8.27E-04
4-hydroxyproline metabolic process (GO:0019471)	12	5	0.11	+	43.84	3.97E-07	1.48E-04
peptidyl-proline hydroxylation (GO:0019511)	13	4	0.12	+	32.38	1.65E-05	2.34E-03
positive regulation of mitochondrial depolarization (GO:0051901)	11	3	0.1	+	28.7	2.77E-04	2.07E-02
mitochondrial fragmentation involved in apoptotic process (GO:0043653)	12	3	0.11	+	26.31	3.44E-04	2.44E-02
positive regulation of membrane depolarization (GO:1904181)	13	3	0.12	+	24.28	4.21E-04	2.88E-02
protein hydroxylation (GO:0018126)	27	6	0.26	+	23.38	5.80E-07	1.94E-04
negative regulation of transcription from RNA polymerase II promoter in response to stress (GO:0097201)	14	3	0.13	+	22.55	5.07E-04	3.30E-02
positive regulation of mitochondrial fission (GO:0090141)	15	3	0.14	+	21.04	6.04E-04	3.81E-02
regulation of autophagy of mitochondrion (GO:1903146)	33	6	0.31	+	19.13	1.63E-06	4.42E-04
positive regulation of autophagy of mitochondrion (GO:1903599)	17	3	0.16	+	18.57	8.33E-04	5.00E-02
positive regulation of release of cytochrome c from mitochondria (GO:0090200)	30	4	0.29	+	14.03	2.83E-04	2.10E-02
glycogen metabolic process (GO:0005977)	49	6	0.47	+	12.88	1.28E-05	2.01E-03
cellular glucan metabolic process (GO:0006073)	49	6	0.47	+	12.88	1.28E-05	1.99E-03
glucan metabolic process (GO:0044042)	49	6	0.47	+	12.88	1.28E-05	1.97E-03
cellular response to glucose starvation (GO:0042149)	43	5	0.41	+	12.23	8.68E-05	8.43E-03
negative regulation of leukocyte migration (GO:0002686)	43	5	0.41	+	12.23	8.68E-05	8.38E-03
cell-substrate junction assembly (GO:0007044)	35	4	0.33	+	12.03	4.85E-04	3.21E-02
platelet-derived growth factor receptor signaling pathway (GO:0048008)	36	4	0.34	+	11.69	5.35E-04	3.43E-02
negative regulation of cell-matrix adhesion (GO:0001953)	37	4	0.35	+	11.38	5.88E-04	3.73E-02
energy reserve metabolic process (GO:0006112)	59	6	0.56	+	10.7	3.37E-05	3.85E-03
regulation of release of cytochrome c from mitochondria (GO:0090199)	50	5	0.48	+	10.52	1.67E-04	1.42E-02
ribonucleoside diphosphate metabolic process (GO:0009185)	81	8	0.77	+	10.39	1.98E-06	5.11E-04
negative regulation of reactive oxygen species metabolic process (GO:2000378)	71	7	0.67	+	10.37	8.87E-06	1.63E-03
positive regulation of mitochondrion organization (GO:0010822)	72	7	0.68	+	10.23	9.66E-06	1.66E-03
negative regulation of lipid biosynthetic process (GO:0051055)	52	5	0.49	+	10.12	1.98E-04	1.58E-02
negative regulation of mitochondrion organization (GO:0010823)	52	5	0.49	+	10.12	1.98E-04	1.57E-02
cellular response to hypoxia (GO:0071456)	159	15	1.51	+	9.93	1.08E-10	1.58E-07
cellular response to decreased oxygen levels (GO:0036294)	172	16	1.63	+	9.79	3.08E-11	6.16E-08

purine ribonucleoside diphosphate metabolic process (GO:0009179)	78	7	0.74	+	9.44	1.57E-05	2.29E-03
purine nucleoside diphosphate metabolic process (GO:0009135)	78	7	0.74	+	9.44	1.57E-05	2.26E-03
regulation of focal adhesion assembly (GO:0051893)	58	5	0.55	+	9.07	3.18E-04	2.30E-02
regulation of cell-substrate junction assembly (GO:0090109)	58	5	0.55	+	9.07	3.18E-04	2.29E-02
regulation of mitochondrion organization (GO:0010821)	130	11	1.24	+	8.9	9.94E-08	4.19E-05
ADP metabolic process (GO:0046031)	71	6	0.67	+	8.89	8.80E-05	8.44E-03
cellular response to oxygen levels (GO:0071453)	191	16	1.82	+	8.81	1.31E-10	1.50E-07
negative regulation of cell-substrate adhesion (GO:0010812)	60	5	0.57	+	8.77	3.68E-04	2.57E-02
carbohydrate catabolic process (GO:0016052)	110	9	1.05	+	8.61	1.93E-06	5.06E-04
nucleoside diphosphate metabolic process (GO:0009132)	98	8	0.93	+	8.59	7.37E-06	1.42E-03
glycolytic process (GO:0006096)	62	5	0.59	+	8.49	4.24E-04	2.89E-02
cellular polysaccharide metabolic process (GO:0044264)	75	6	0.71	+	8.42	1.17E-04	1.06E-02
polysaccharide metabolic process (GO:0005976)	76	6	0.72	+	8.31	1.25E-04	1.12E-02
response to hypoxia (GO:0001666)	408	32	3.88	+	8.25	2.20E-19	3.53E-15
ATP generation from ADP (GO:0006757)	64	5	0.61	+	8.22	4.85E-04	3.20E-02
regulation of adherens junction organization (GO:1903391)	64	5	0.61	+	8.22	4.85E-04	3.19E-02
apoptotic mitochondrial changes (GO:0008637)	65	5	0.62	+	8.09	5.19E-04	3.37E-02
response to decreased oxygen levels (GO:0036293)	423	32	4.02	+	7.96	5.96E-19	4.77E-15
response to unfolded protein (GO:0006986)	93	7	0.88	+	7.92	4.52E-05	4.79E-03
nucleoside diphosphate phosphorylation (GO:0006165)	81	6	0.77	+	7.79	1.73E-04	1.44E-02
regulation of mitochondrial membrane potential (GO:0051881)	82	6	0.78	+	7.7	1.85E-04	1.50E-02
nucleotide phosphorylation (GO:0046939)	83	6	0.79	+	7.61	1.96E-04	1.57E-02
cellular response to unfolded protein (GO:0034620)	71	5	0.67	+	7.41	7.57E-04	4.59E-02
pyruvate metabolic process (GO:0006090)	86	6	0.82	+	7.34	2.35E-04	1.81E-02
response to oxygen levels (GO:0070482)	463	32	4.4	+	7.27	7.07E-18	3.78E-14
regulation of cell-matrix adhesion (GO:0001952)	116	8	1.1	+	7.26	2.33E-05	2.89E-03
regulation of ATP metabolic process (GO:1903578)	90	6	0.86	+	7.01	2.97E-04	2.18E-02
extrinsic apoptotic signaling pathway (GO:0097191)	91	6	0.86	+	6.94	3.14E-04	2.29E-02
cellular response to starvation (GO:0009267)	162	10	1.54	+	6.5	5.59E-06	1.16E-03
glucose metabolic process (GO:0006006)	98	6	0.93	+	6.44	4.56E-04	3.05E-02
response to topologically incorrect protein (GO:0035966)	116	7	1.1	+	6.35	1.67E-04	1.43E-02
response to starvation (GO:0042594)	223	13	2.12	+	6.13	4.00E-07	1.46E-04
cellular modified amino acid metabolic process (GO:0006575)	177	10	1.68	+	5.94	1.17E-05	1.93E-03
positive regulation of autophagy (GO:0010508)	145	8	1.38	+	5.81	1.04E-04	9.66E-03
hexose metabolic process (GO:0019318)	128	7	1.22	+	5.75	2.96E-04	2.19E-02
regulation of cellular ketone metabolic process (GO:0010565)	130	7	1.24	+	5.67	3.24E-04	2.33E-02
negative regulation of immune effector process (GO:0002698)	135	7	1.28	+	5.46	4.02E-04	2.78E-02
cellular carbohydrate metabolic process (GO:0044262)	158	8	1.5	+	5.33	1.82E-04	1.49E-02
regulation of reactive oxygen species metabolic process (GO:2000377)	205	10	1.95	+	5.13	3.88E-05	4.31E-03
regulation of angiogenesis (GO:0045765)	297	14	2.82	+	4.96	1.60E-06	4.43E-04
cellular response to nutrient levels (GO:0031669)	263	12	2.5	+	4.8	1.24E-05	2.00E-03
regulation of vasculature development (GO:1901342)	330	15	3.14	+	4.78	1.04E-06	3.04E-04
positive regulation of angiogenesis (GO:0045766)	178	8	1.69	+	4.73	3.93E-04	2.73E-02
cellular response to insulin stimulus (GO:0032869)	181	8	1.72	+	4.65	4.37E-04	2.94E-02

response to insulin (GO:0032868)	272	12	2.59	+	4.64	1.70E-05	2.36E-03
apoptotic signaling pathway (GO:0097190)	273	12	2.59	+	4.63	1.77E-05	2.38E-03
cellular response to extracellular stimulus (GO:0031668)	299	13	2.84	+	4.57	8.79E-06	1.64E-03
negative regulation of cellular component movement (GO:0051271)	325	14	3.09	+	4.53	4.37E-06	9.72E-04
response to temperature stimulus (GO:0009266)	233	10	2.21	+	4.52	1.08E-04	9.92E-03
extracellular matrix organization (GO:0030198)	234	10	2.22	+	4.5	1.11E-04	1.01E-02
alpha-amino acid metabolic process (GO:1901605)	189	8	1.8	+	4.45	5.75E-04	3.67E-02
negative regulation of cell motility (GO:2000146)	290	12	2.76	+	4.35	3.12E-05	3.65E-03
regulation of cell-substrate adhesion (GO:0010810)	218	9	2.07	+	4.34	3.13E-04	2.29E-02
regulation of autophagy (GO:0010506)	268	11	2.55	+	4.32	7.17E-05	7.32E-03
female pregnancy (GO:0007565)	269	11	2.56	+	4.3	7.40E-05	7.46E-03
positive regulation of vasculature development (GO:1904018)	197	8	1.87	+	4.27	7.46E-04	4.56E-02
negative regulation of cell migration (GO:0030336)	274	11	2.6	+	4.22	8.67E-05	8.47E-03
response to estradiol (GO:0032355)	251	10	2.39	+	4.19	1.93E-04	1.56E-02
multi-multicellular organism process (GO:0044706)	303	12	2.88	+	4.17	4.70E-05	4.95E-03
response to monosaccharide (GO:0034284)	228	9	2.17	+	4.15	4.29E-04	2.91E-02
negative regulation of cell adhesion (GO:0007162)	279	11	2.65	+	4.15	1.01E-04	9.48E-03
negative regulation of locomotion (GO:0040013)	341	13	3.24	+	4.01	3.31E-05	3.82E-03
response to abiotic stimulus (GO:0009628)	1364	52	12.96	+	4.01	7.59E-18	3.04E-14
cellular response to external stimulus (GO:0071496)	399	15	3.79	+	3.96	9.58E-06	1.67E-03
extracellular structure organization (GO:0043062)	269	10	2.56	+	3.91	3.28E-04	2.35E-02
positive regulation of catabolic process (GO:0009896)	449	16	4.27	+	3.75	9.17E-06	1.63E-03
transmembrane receptor protein tyrosine kinase signaling pathway (GO:0007169)	339	12	3.22	+	3.72	1.31E-04	1.17E-02
carbohydrate metabolic process (GO:0005975)	428	15	4.07	+	3.69	2.12E-05	2.72E-03
regulation of catabolic process (GO:0009894)	845	29	8.03	+	3.61	3.72E-09	2.60E-06
generation of precursor metabolites and energy (GO:0006091)	351	12	3.34	+	3.6	1.79E-04	1.47E-02
regulation of small molecule metabolic process (GO:0062012)	336	11	3.19	+	3.44	4.73E-04	3.14E-02
regulation of neuron death (GO:1901214)	378	12	3.59	+	3.34	3.44E-04	2.45E-02
regulation of cell migration (GO:0030334)	897	28	8.52	+	3.28	5.02E-08	2.52E-05
regulation of immune effector process (GO:0002697)	397	12	3.77	+	3.18	5.26E-04	3.38E-02
regulation of cellular component movement (GO:0051270)	1033	31	9.82	+	3.16	2.13E-08	1.22E-05
programmed cell death (GO:0012501)	735	22	6.99	+	3.15	2.96E-06	7.08E-04
cell death (GO:0008219)	775	23	7.37	+	3.12	2.00E-06	5.08E-04
positive regulation of cell death (GO:0010942)	710	21	6.75	+	3.11	6.02E-06	1.22E-03
regulation of cell motility (GO:2000145)	948	28	9.01	+	3.11	1.52E-07	6.11E-05
response to peptide hormone (GO:0043434)	509	15	4.84	+	3.1	1.40E-04	1.24E-02
aging (GO:0007568)	447	13	4.25	+	3.06	4.41E-04	2.96E-02
regulation of cell adhesion (GO:0030155)	690	20	6.56	+	3.05	1.35E-05	2.04E-03
apoptotic process (GO:0006915)	701	20	6.66	+	3	1.68E-05	2.36E-03
regulation of cellular catabolic process (GO:0031329)	703	20	6.68	+	2.99	1.75E-05	2.38E-03
response to peptide (GO:1901652)	598	17	5.68	+	2.99	7.71E-05	7.73E-03
regulation of locomotion (GO:0040012)	1023	29	9.72	+	2.98	2.06E-07	8.07E-05
enzyme linked receptor protein signaling pathway (GO:0007167)	530	15	5.04	+	2.98	2.15E-04	1.68E-02
response to extracellular stimulus (GO:0009991)	747	21	7.1	+	2.96	1.27E-05	2.03E-03

negative regulation of cell population proliferation (GO:0008285)	680	19	6.46	+	2.94	3.63E-05	4.10E-03
response to nutrient levels (GO:0031667)	717	20	6.81	+	2.94	2.30E-05	2.88E-03
negative regulation of immune system process (GO:0002683)	468	13	4.45	+	2.92	6.69E-04	4.14E-02
positive regulation of organelle organization (GO:0010638)	593	16	5.64	+	2.84	2.24E-04	1.73E-02
cellular response to lipid (GO:0071396)	633	17	6.02	+	2.83	1.50E-04	1.29E-02
cellular response to stress (GO:0033554)	1540	41	14.64	+	2.8	2.37E-09	1.81E-06
positive regulation of apoptotic process (GO:0043065)	642	17	6.1	+	2.79	1.76E-04	1.46E-02
negative regulation of cellular biosynthetic process (GO:0031327)	1476	39	14.03	+	2.78	7.73E-09	4.96E-06
positive regulation of programmed cell death (GO:0043068)	647	17	6.15	+	2.76	1.93E-04	1.55E-02
regulation of cytokine production (GO:0001817)	691	18	6.57	+	2.74	1.38E-04	1.23E-02
carboxylic acid metabolic process (GO:0019752)	811	21	7.71	+	2.72	4.11E-05	4.51E-03
negative regulation of biosynthetic process (GO:0009890)	1513	39	14.38	+	2.71	1.50E-08	8.88E-06
negative regulation of transcription by RNA polymerase II (GO:0000122)	855	22	8.13	+	2.71	2.93E-05	3.51E-03
positive regulation of cellular component movement (GO:0051272)	587	15	5.58	+	2.69	6.09E-04	3.83E-02
regulation of anatomical structure morphogenesis (GO:0022603)	1057	27	10.05	+	2.69	3.87E-06	8.73E-04
cellular response to hormone stimulus (GO:0032870)	667	17	6.34	+	2.68	2.72E-04	2.04E-02
cellular response to cytokine stimulus (GO:0071345)	759	19	7.21	+	2.63	1.49E-04	1.29E-02
response to cytokine (GO:0034097)	894	22	8.5	+	2.59	5.60E-05	5.83E-03
oxoacid metabolic process (GO:0043436)	854	21	8.12	+	2.59	8.42E-05	8.28E-03
cellular response to organic cyclic compound (GO:0071407)	654	16	6.22	+	2.57	6.35E-04	3.96E-02
regulation of response to external stimulus (GO:0032101)	944	23	8.97	+	2.56	4.37E-05	4.67E-03
negative regulation of response to stimulus (GO:0048585)	1563	38	14.85	+	2.56	1.05E-07	4.32E-05
response to lipid (GO:0033993)	1196	29	11.37	+	2.55	4.41E-06	9.69E-04
response to hormone (GO:0009725)	1200	29	11.4	+	2.54	4.70E-06	1.02E-03
negative regulation of signal transduction (GO:0009968)	1162	28	11.04	+	2.54	7.36E-06	1.44E-03
organic acid metabolic process (GO:0006082)	879	21	8.35	+	2.51	1.25E-04	1.12E-02
regulation of organelle organization (GO:0033043)	1228	29	11.67	+	2.48	7.24E-06	1.43E-03
negative regulation of cellular macromolecule biosynthetic process (GO:2000113)	1364	32	12.96	+	2.47	3.80E-06	8.70E-04
negative regulation of cell communication (GO:0010648)	1284	30	12.2	+	2.46	8.77E-06	1.65E-03
negative regulation of RNA biosynthetic process (GO:1902679)	1201	28	11.41	+	2.45	1.97E-05	2.58E-03
negative regulation of signaling (GO:0023057)	1287	30	12.23	+	2.45	8.96E-06	1.63E-03
negative regulation of macromolecule biosynthetic process (GO:0010558)	1422	33	13.51	+	2.44	2.90E-06	7.03E-04
negative regulation of developmental process (GO:0051093)	1038	24	9.86	+	2.43	9.96E-05	9.39E-03
negative regulation of gene expression (GO:0010629)	1601	37	15.22	+	2.43	6.41E-07	2.10E-04
negative regulation of cellular metabolic process (GO:0031324)	2512	58	23.87	+	2.43	1.15E-10	1.53E-07
negative regulation of multicellular organismal process (GO:0051241)	1301	30	12.36	+	2.43	1.01E-05	1.72E-03
negative regulation of transcription, DNA-templated (GO:0045892)	1172	27	11.14	+	2.42	3.23E-05	3.75E-03
response to organonitrogen compound (GO:0010243)	1265	29	12.02	+	2.41	1.58E-05	2.27E-03
negative regulation of nucleobase-containing compound metabolic process (GO:0045934)	1396	32	13.27	+	2.41	5.06E-06	1.07E-03

small molecule metabolic process (GO:0044281)	1490	34	14.16	+	2.4	2.54E-06	6.36E-04
cellular response to organic substance (GO:0071310)	2194	50	20.85	+	2.4	5.09E-09	3.40E-06
negative regulation of apoptotic process (GO:0043066)	925	21	8.79	+	2.39	3.52E-04	2.49E-02
negative regulation of metabolic process (GO:0009892)	2775	63	26.37	+	2.39	3.47E-11	6.17E-08
response to stress (GO:0006950)	3196	72	30.37	+	2.37	7.30E-13	2.34E-09
negative regulation of nucleic acid-templated transcription (GO:1903507)	1199	27	11.4	+	2.37	4.14E-05	4.52E-03
negative regulation of RNA metabolic process (GO:0051253)	1288	29	12.24	+	2.37	2.00E-05	2.59E-03
regulation of cell death (GO:0010941)	1698	38	16.14	+	2.35	8.38E-07	2.53E-04
regulation of transferase activity (GO:0051338)	897	20	8.52	+	2.35	5.76E-04	3.66E-02
negative regulation of programmed cell death (GO:0043069)	943	21	8.96	+	2.34	4.05E-04	2.79E-02
response to nitrogen compound (GO:1901698)	1349	30	12.82	+	2.34	1.68E-05	2.35E-03
cellular response to chemical stimulus (GO:0070887)	2748	60	26.12	+	2.3	6.34E-10	5.34E-07
negative regulation of cell death (GO:0060548)	1055	23	10.03	+	2.29	2.48E-04	1.86E-02
circulatory system development (GO:0072359)	925	20	8.79	+	2.28	7.40E-04	4.54E-02
response to organic cyclic compound (GO:0014070)	1308	28	12.43	+	2.25	9.04E-05	8.62E-03
regulation of programmed cell death (GO:0043067)	1544	33	14.67	+	2.25	1.55E-05	2.27E-03
response to oxygen-containing compound (GO:1901700)	1968	42	18.7	+	2.25	6.91E-07	2.17E-04
positive regulation of protein metabolic process (GO:0051247)	1693	36	16.09	+	2.24	5.90E-06	1.21E-03
negative regulation of nitrogen compound metabolic process (GO:0051172)	2309	49	21.94	+	2.23	8.64E-08	3.74E-05
response to organic substance (GO:0010033)	3175	67	30.17	+	2.22	1.37E-10	1.46E-07
regulation of cell population proliferation (GO:0042127)	1659	35	15.77	+	2.22	9.47E-06	1.67E-03
regulation of apoptotic process (GO:0042981)	1520	32	14.45	+	2.22	2.67E-05	3.25E-03
positive regulation of catalytic activity (GO:0043085)	1259	26	11.97	+	2.17	2.44E-04	1.85E-02
cellular response to oxygen-containing compound (GO:1901701)	1216	25	11.56	+	2.16	3.53E-04	2.48E-02
cell surface receptor signaling pathway (GO:0007166)	1706	35	16.21	+	2.16	2.14E-05	2.73E-03
positive regulation of cellular metabolic process (GO:0031325)	3281	67	31.18	+	2.15	5.33E-10	4.75E-07
positive regulation of metabolic process (GO:0009893)	3559	72	33.82	+	2.13	1.53E-10	1.53E-07
positive regulation of cellular protein metabolic process (GO:0032270)	1591	32	15.12	+	2.12	7.22E-05	7.32E-03
proteolysis (GO:0006508)	1145	23	10.88	+	2.11	8.20E-04	4.94E-02
response to endogenous stimulus (GO:0009719)	1749	35	16.62	+	2.11	2.96E-05	3.51E-03
organic substance catabolic process (GO:1901575)	1459	29	13.87	+	2.09	2.14E-04	1.68E-02
negative regulation of macromolecule metabolic process (GO:0010605)	2516	50	23.91	+	2.09	5.19E-07	1.81E-04
regulation of localization (GO:0032879)	2875	57	27.32	+	2.09	4.76E-08	2.46E-05
response to external stimulus (GO:0009605)	2432	48	23.11	+	2.08	1.15E-06	3.29E-04
regulation of protein modification process (GO:0031399)	1827	36	17.36	+	2.07	4.02E-05	4.44E-03
response to other organism (GO:0051707)	1219	24	11.59	+	2.07	7.49E-04	4.56E-02
response to external biotic stimulus (GO:0043207)	1223	24	11.62	+	2.06	7.77E-04	4.70E-02
regulation of response to stress (GO:0080134)	1337	26	12.71	+	2.05	6.59E-04	4.10E-02
regulation of immune system process (GO:0002682)	1389	27	13.2	+	2.05	4.86E-04	3.18E-02
regulation of multicellular organismal development (GO:2000026)	2113	41	20.08	+	2.04	1.55E-05	2.29E-03
homeostatic process (GO:0042592)	1653	32	15.71	+	2.04	1.73E-04	1.44E-02

positive regulation of nitrogen compound metabolic process (GO:0051173)	3122	60	29.67	+	2.02	7.09E-08	3.16E-05
positive regulation of multicellular organismal process (GO:0051240)	1935	37	18.39	+	2.01	4.29E-05	4.62E-03
regulation of catalytic activity (GO:0050790)	2112	40	20.07	+	1.99	2.75E-05	3.31E-03
regulation of phosphate metabolic process (GO:0019220)	1755	33	16.68	+	1.98	1.70E-04	1.45E-02
regulation of phosphorus metabolic process (GO:0051174)	1756	33	16.69	+	1.98	1.72E-04	1.45E-02
regulation of protein metabolic process (GO:0051246)	2750	51	26.14	+	1.95	2.64E-06	6.52E-04
regulation of developmental process (GO:0050793)	2644	49	25.13	+	1.95	5.04E-06	1.08E-03
phosphate-containing compound metabolic process (GO:0006796)	1836	34	17.45	+	1.95	2.16E-04	1.68E-02
negative regulation of cellular process (GO:0048523)	4596	85	43.68	+	1.95	9.69E-11	1.55E-07
positive regulation of molecular function (GO:0044093)	1634	30	15.53	+	1.93	5.19E-04	3.35E-02
phosphorus metabolic process (GO:0006793)	1856	34	17.64	+	1.93	2.40E-04	1.84E-02
positive regulation of macromolecule metabolic process (GO:0010604)	3281	60	31.18	+	1.92	4.11E-07	1.46E-04
regulation of multicellular organismal process (GO:0051239)	3175	58	30.17	+	1.92	8.25E-07	2.54E-04
regulation of cellular metabolic process (GO:0031323)	5533	100	52.58	+	1.9	1.60E-12	4.26E-09
regulation of transcription by RNA polymerase II (GO:0006357)	2004	36	19.05	+	1.89	2.41E-04	1.83E-02
regulation of cellular protein metabolic process (GO:0032268)	2572	46	24.44	+	1.88	3.06E-05	3.61E-03
regulation of nitrogen compound metabolic process (GO:0051171)	5200	92	49.42	+	1.86	1.16E-10	1.43E-07
negative regulation of biological process (GO:0048519)	5097	90	48.44	+	1.86	2.66E-10	2.50E-07
regulation of cellular biosynthetic process (GO:0031326)	3584	63	34.06	+	1.85	6.77E-07	2.17E-04
regulation of nucleobase-containing compound metabolic process (GO:0019219)	3419	60	32.49	+	1.85	1.81E-06	4.84E-04
regulation of biosynthetic process (GO:0009889)	3656	64	34.75	+	1.84	5.45E-07	1.86E-04
regulation of metabolic process (GO:0019222)	5923	103	56.29	+	1.83	8.49E-12	1.94E-08
regulation of RNA biosynthetic process (GO:2001141)	2942	51	27.96	+	1.82	2.21E-05	2.78E-03
protein metabolic process (GO:0019538)	3802	65	36.13	+	1.8	1.23E-06	3.45E-04
regulation of nucleic acid-templated transcription (GO:1903506)	2937	50	27.91	+	1.79	3.62E-05	4.12E-03
regulation of transcription, DNA-templated (GO:0006355)	2893	49	27.49	+	1.78	5.10E-05	5.34E-03
organonitrogen compound metabolic process (GO:1901564)	4670	79	44.38	+	1.78	5.21E-08	2.45E-05
regulation of primary metabolic process (GO:0080090)	5350	90	50.85	+	1.77	2.94E-09	2.14E-06
regulation of signal transduction (GO:0009966)	2921	49	27.76	+	1.77	8.23E-05	8.14E-03
regulation of cellular component organization (GO:0051128)	2459	41	23.37	+	1.75	3.69E-04	2.57E-02
multi-organism process (GO:0051704)	2341	39	22.25	+	1.75	6.33E-04	3.96E-02
regulation of RNA metabolic process (GO:0051252)	3183	53	30.25	+	1.75	4.15E-05	4.50E-03
regulation of macromolecule biosynthetic process (GO:0010556)	3435	57	32.65	+	1.75	2.00E-05	2.61E-03
regulation of cellular macromolecule biosynthetic process (GO:2000112)	3317	55	31.52	+	1.74	2.53E-05	3.12E-03
positive regulation of cellular process (GO:0048522)	5324	88	50.6	+	1.74	1.28E-08	7.90E-06
regulation of molecular function (GO:0065009)	3038	50	28.87	+	1.73	1.09E-04	9.94E-03
positive regulation of biological process (GO:0048518)	5975	98	56.79	+	1.73	1.38E-09	1.10E-06
regulation of gene expression (GO:0010468)	3781	62	35.93	+	1.73	1.17E-05	1.91E-03
regulation of cell communication (GO:0010646)	3330	54	31.65	+	1.71	6.06E-05	6.26E-03
regulation of signaling (GO:0023051)	3347	54	31.81	+	1.7	9.16E-05	8.69E-03

regulation of macromolecule metabolic process (GO:0060255)	5461	88	51.9	+	1.7	4.54E-08	2.51E-05
regulation of biological quality (GO:0065008)	3947	63	37.51	+	1.68	1.73E-05	2.37E-03
animal organ development (GO:0048513)	3394	54	32.26	+	1.67	1.04E-04	9.63E-03
developmental process (GO:0032502)	5559	87	52.83	+	1.65	3.13E-07	1.19E-04
system development (GO:0048731)	4478	69	42.56	+	1.62	1.93E-05	2.56E-03
multicellular organism development (GO:0007275)	4872	75	46.3	+	1.62	7.13E-06	1.43E-03
macromolecule metabolic process (GO:0043170)	5399	82	51.31	+	1.6	3.12E-06	7.36E-04
primary metabolic process (GO:0044238)	6604	100	62.76	+	1.59	6.92E-08	3.17E-05
regulation of response to stimulus (GO:0048583)	3897	59	37.04	+	1.59	1.72E-04	1.44E-02
nitrogen compound metabolic process (GO:0006807)	6077	91	57.75	+	1.58	1.02E-06	3.03E-04
organic substance metabolic process (GO:0071704)	7012	105	66.64	+	1.58	4.64E-08	2.48E-05
anatomical structure development (GO:0048856)	5239	78	49.79	+	1.57	1.27E-05	2.02E-03
response to chemical (GO:0042221)	5308	79	50.45	+	1.57	1.33E-05	2.03E-03
cellular macromolecule metabolic process (GO:0044260)	4271	63	40.59	+	1.55	2.02E-04	1.60E-02
metabolic process (GO:0008152)	7516	110	71.43	+	1.54	5.18E-08	2.52E-05
cellular metabolic process (GO:0044237)	6675	94	63.44	+	1.48	9.11E-06	1.64E-03
cellular response to stimulus (GO:0051716)	6811	91	64.73	+	1.41	1.46E-04	1.28E-02
response to stimulus (GO:0050896)	8503	112	80.81	+	1.39	1.40E-05	2.09E-03
regulation of cellular process (GO:0050794)	10795	129	102.59	+	1.26	2.40E-04	1.83E-02
biological regulation (GO:0065007)	12088	143	114.88	+	1.24	6.57E-05	6.75E-03
regulation of biological process (GO:0050789)	11421	133	108.54	+	1.23	6.80E-04	4.19E-02
cellular process (GO:0009987)	14066	159	133.68	+	1.19	1.47E-04	1.28E-02
G protein-coupled receptor signaling pathway (GO:0007186)	2057	6	19.55	-	0.31	4.35E-04	2.94E-02
sensory perception of chemical stimulus (GO:0007606)	1298	1	12.34	-	0.08	8.04E-05	8.01E-03
detection of stimulus (GO:0051606)	1386	1	13.17	-	0.08	3.67E-05	4.12E-03
detection of chemical stimulus involved in sensory perception of smell (GO:0050911)	1160	0	11.02	-	< 0.01	2.59E-05	3.17E-03
detection of chemical stimulus involved in sensory perception (GO:0050907)	1217	0	11.57	-	< 0.01	1.10E-05	1.86E-03
detection of stimulus involved in sensory perception (GO:0050906)	1291	0	12.27	-	< 0.01	7.68E-06	1.47E-03
detection of chemical stimulus (GO:0009593)	1248	0	11.86	-	< 0.01	1.16E-05	1.93E-03
sensory perception of smell (GO:0007608)	1216	0	11.56	-	< 0.01	1.82E-05	2.43E-03
