# Supplementary material for: Predicted rat interactome database and gene set linkage analysis
Source: Database (Oxford). 2020 Nov 20;2020:baaa086. doi: 10.1093/database/baaa086 (PMC7678787; doi:10.1093/database/baaa086)
Supplement: baaa086_Supp [file baaa086_supp.zip › Table_S9.pdf]

Supplementary Table 9. Annotations reported by PRID/GSLA for the top 250 transcriptionally changed genes.

Job Number: GSLA08076795617586
Cutoff used:
Q1: density >= 0.015
Q2: p <= 0.001
Meaning:
Q1: inter-geneset interaction density is greater than expected.
Q2: the observed interaction density can only be observed in the biologically correct interactome topology.
Categories selected: GO biological process
Species: Rattus norvegicus
Interaction dataset selected: PRID

#Subjob Number: GSLA08076795617586_0
#Description: #up-TOP250

#Quaried GeneSet: Mmp12, Ak4, Ankrd37, Fam65b, Kdm3a, Pygl, Zfp185, Aldoc, Ccl27, Gbe1, Gpt, LOC100911625///Eno2, Nol4, P4ha1, Basp1, Ctla2a, Ifi27l2b, Nudt22, Spsb1, Arrdc3, LOC691807, Efemp2, Pdk1, Plod2, Rasd1, Tbl2, Ceacam15, Plpp1, Vegfa, Dlc1, Il1r2, Il33, Ddit4, Asic1, Dpp3l///LOC678760///Dpp3, Stbd1, RGD1309104, Slc25a36, Acp5, Cd200, Slc35e1, Ppp1r3c, Bnip3, Nr4a3, Bhlhe40, Egln1, Il33, Mllt11, Narf, Pfkfb3, Rimklb, Tep1, Wwp1, Rcbtb1, Rcc1l, Greb1l, Pgm1, Guca1a, Phtf1, Vsir, Bcl11a, Casp6, Cdcp1, Fam46a, Foxo3, Hk2, Kdr, Ndrg1, Pgm1, Ppm1j, Prepl, Spidr, Cdcp1, Efemp2, Mmp9, Proser2, Vldlr, Gng2, Hspa1b///Hspa1a, Mysm1, Slc22a5, Ell, Greb1l, Noct, Vwa1, Ero1a, Hilpda, Il1rap, Klc4, Parp16, Scrn1, Mab21l3, Vldlr, P4ha2, Ddx50, LOC100911293///LOC691658///Aph1b, Rnf138, Vegfa, Abhd18, Efna1, Higd1a, LOC102550354, Smtnl2, Zc3h7b, Cpm, Sec22b, Tiparp, LOC501445///Derl1, Dusp1, Adamts9, Eif2ak4, Higd1a, Id3, Map3k8, Slc27a1, Bnip3l, Fbln1, Insig2, Mex3b, Nptx1, Ramp3, RGD1560795///Spr, RGD1562037, Spsb1, Insig1, Insig2, Sphk1, Zfp503, Dnajb1, Tspo, Plau, Hp, Rnf125, Chpt1, Clcn3, Hmx1, Clcn3, Eif4e3, Nfil3, Bnip3l, Acvrl1, Gpr1, Syne1, Prkcq, Acap1, Eif4ebp1, Hltf, LOC100910685///Gcat, Pard6b, Wnt4, Cpm, LOC100909732///Fam122b, Maff, Mat2b, Foxo4, Gpi, LOC100912399///Map3k1, Appl1, Atp11b, Slc2a1, Tceal9, Bcr, Arl16, Gzf1, Cdkn1b, Hagh, Hbp1, Rsrp1, Vldlr, Zap70, Arl16, LOC100910137///Triap1, Hspa1a, Il2rb, Alkbh5, Comt, Lrif1, Mmp9, Zmynd10, Rsrp1, Tspan33, LOC683062///Vdac1, Faslg, Ero1a, Prkag2, Vopp1, Ndrg1, Prrc1, Zc3h7b, Scpep1, Entpd2, Lrrc8a, Cr1l, Hexb, Slc27a3, Arrdc3, Kdm4b, LOC102553917, Entpd4, Prkag2, Ccng2, Dst, Ksr1, Pdk1, LOC100911874///Slc40a1, Bcl6, Dnm1l, Rybp, Jmjd6, Bmt2, Hikeshi, Mnt, Slc6a8, Tomm7, Pfkl, Asic1, Dnajc3, Tpgs2, Dtna, Fcna, Cited2, Dennd3, Mllt3, Khnyn, Dnajb9, Tspan31, Actn1, Cblb, Upp1, Ifi27, Slc16a3, Kctd11, LOC100911642///LOC680835, Plin3, Kazald1, Asic1, Lrg1, Pgk1, Surf4, Arhgap8, Ctsm, Egln3, Xkr8, Mturn, Prrc1, Sqle, Clcn3, Tmem59, Fiz1, Clcn3

Term type	Term	Description	Term size	P value	Density	Interaction number	Overlap gene number	Overlap gene(s)	Interactions
GO biological process	GO:0097194	execution phase of apoptosis	12	3.00E-04	0.01504	37	0		Appl1-RGD:2081|Appl1-RGD:708455|Arrdc3-RGD:2275|Comt-RGD:1561742|Acap1-RGD:2081|Foxo3-RGD:2081|Sec22b-RGD:620945|Sec22b-RGD:2275|Egln1-RGD:2081|Foxo4-RGD:2081|Vwa1-RGD:2081|Faslg-RGD:620945|Faslg-RGD:2275|Dnajc3-RGD:1561742|Hbp1-RGD:2081|Hspa1a-RGD:2081|Slc22a5-RGD:2081|Cblb-RGD:2275|Eif4ebp1-RGD:2081|Efna1-RGD:620945|Efna1-RGD:2275|Casp6-RGD:620945|Casp6-RGD:620944|Casp6-RGD:2275|Insig1-RGD:2081|Slc27a1-RGD:1562113|Nr4a3-RGD:2081|Nptx1-RGD:1562113|Vopp1-RGD:2081|Prrc1-RGD:2081|Pfkl-RGD:619922|Bcr-RGD:2081|Cdkn1b-RGD:2275|Vldlr-RGD:708455|Nol4-RGD:2081|Dusp1-RGD:2081|Dlc1-RGD:2081

<table>
<tr><th></th><th></th><th></th><th></th><th></th><th></th><th></th><th></th><th></th><th></th></tr>
<tr><td>GO biological process</td><td>GO:1902108</td><td>regulation of mitochondrial membrane permeability involved in apoptotic process</td><td>19</td><td>0</td><td>0.01771</td><td>69</td><td>1</td><td>Bnip3</td><td>Sqle-RGD:2192|Gpt-RGD:1306551|Foxo3-RGD:3889|Sec22b-RGD:1306551|Sec22b-RGD:2261|Ero1a-RGD:2192|Ndrg1-RGD:628774|Dnajc3-RGD:620160|Cblb-RGD:1306551|Eif4ebp1-RGD:3889|Kdm3a-RGD:3889|Tspo-RGD:620352|Tspo-RGD:620160|Tspo-Bnip3|Tspo-RGD:628774|Tspo-RGD:2192|Dnm1l-RGD:1308080|Dnm1l-RGD:620352|Dnm1l-RGD:620160|Dnm1l-RGD:628774|Dnm1l-RGD:708558|Dnm1l-RGD:727970|Bnip3-RGD:1308080|Bnip3-RGD:1307023|Bnip3-RGD:3889|Bnip3-RGD:620482|Bnip3-RGD:2261|Insig1-RGD:3889|Prkcq-RGD:3889|Prkcq-RGD:621862|Prkcq-RGD:2261|Tomm7-RGD:1307023|Tomm7-RGD:620160|Tomm7-Bnip3|Tomm7-RGD:708558|Higd1a-RGD:620352|Higd1a-RGD:2192|Higd1a-RGD:621862|Higd1a-RGD:708558|Higd1a-RGD:727970|Bnip3l-RGD:1308080|Bnip3l-RGD:620352|Bnip3l-Bnip3|Slc27a1-RGD:708558|Il33-RGD:620160|Clcn3-RGD:2192|Zmynd10-RGD:2192|Zmynd10-RGD:3889|Pdk1-RGD:708558|Pfkl-RGD:3889|Egln3-RGD:3889|Prepl-RGD:621862|Prepl-RGD:708558|Bcl6-RGD:3889|Vegfa-RGD:1306551|Ell-RGD:3889|Kdr-Bnip3|Kdr-RGD:2192|Kdr-RGD:3889|Actn1-RGD:2261|Vldlr-RGD:1306551|Vldlr-RGD:708558|Dnajb9-RGD:2192|Hk2-RGD:620352|Hk2-Bnip3|Hk2-RGD:2192|Hk2-RGD:620482|Hk2-RGD:708558|Hk2-RGD:727970</td></tr>
<tr><td>GO biological process</td><td>GO:0071279</td><td>cellular response to cobalt ion</td><td>6</td><td>0</td><td>0.02195</td><td>27</td><td>1</td><td>Bnip3</td><td>Bhlhe40-RGD:61928|Foxo3-RGD:61928|Alkbh5-RGD:619831|Egln1-RGD:61928|Hikeshi-RGD:619831|Faslg-RGD:619831|Hbp1-RGD:61928|Mmp12-RGD:2179|Kdm3a-RGD:61928|Tspo-Bnip3|Bnip3-RGD:631369|Bnip3-RGD:61928|Tomm7-RGD:1560038|Tomm7-Bnip3|Bnip3l-Bnip3|Dnajb1-RGD:61928|Wnt4-RGD:619831|Pdk1-RGD:61928|Pfkl-RGD:61928|Egln3-RGD:61928|Atp11b-RGD:2179|Vegfa-RGD:61928|Kdr-Bnip3|Pgk1-RGD:61928|Dnajb9-RGD:61928|Hk2-RGD:1560038|Hk2-Bnip3</td></tr>
<tr><td>GO biological process</td><td>GO:0018401</td><td>peptidyl-proline hydroxylation to 4-hydroxy-L-proline</td><td>9</td><td>0</td><td>0.01571</td><td>29</td><td>5</td><td>P4ha1|Egln3|Ero1a|P4ha2|Egln1</td><td>P4ha1-Egln3|P4ha1-RGD:3244|P4ha1-P4ha2|P4ha2-P4ha1|P4ha2-Egln3|P4ha2-RGD:3244|P4ha2-RGD:631376|P4ha2-Egln1|Alkbh5-RGD:3244|Ero1a-RGD:1563548|Ero1a-RGD:3244|Egln1-Egln3|Egln1-RGD:3244|Egln1-RGD:631376|Egln1-P4ha2|Dnajc3-RGD:620043|Surf4-RGD:3244|Jmjd6-P4ha1|Jmjd6-Egln3|Dnm1l-Egln3|Dnm1l-RGD:631376|Egln3-P4ha1|Egln3-RGD:631376|Egln3-P4ha2|Egln3-Egln1|Dnajb9-Ero1a|Plod2-P4ha1|Plod2-RGD:3244|Plod2-P4ha2</td></tr>
<tr><td>GO biological process</td><td>GO:1905710</td><td>positive regulation of membrane permeability</td><td>20</td><td>0</td><td>0.0156</td><td>64</td><td>2</td><td>Bnip3l|Bnip3</td><td>Sqle-RGD:2192|Gpt-RGD:1306551|Foxo3-RGD:3889|Sec22b-RGD:1306551|Ero1a-RGD:2192|Vwa1-RGD:3718|Ndrg1-RGD:628774|Dnajc3-RGD:620160|Dnajc3-RGD:3718|Cblb-RGD:1306551|Eif4ebp1-RGD:3889|Kdm3a-RGD:3889|Tspo-RGD:620160|Tspo-Bnip3|Tspo-RGD:621252|Tspo-RGD:628774|Tspo-RGD:2192|Dnm1l-RGD:620160|Dnm1l-RGD:621252|Dnm1l-RGD:628774|Dnm1l-RGD:727970|Bnip3-Bnip3l|Bnip3-RGD:1307023|Bnip3-RGD:3889|Hltf-RGD:621725|Insig1-RGD:3889|Prkcq-RGD:3889|Prkcq-RGD:621862|Tomm7-Bnip3l|Tomm7-RGD:1307023|Tomm7-RGD:620160|Tomm7-Bnip3|Higd1a-RGD:628670|Higd1a-RGD:2192|Higd1a-RGD:621862|Higd1a-RGD:727970|Bnip3l-Bnip3|Bnip3l-RGD:621252|Il33-RGD:620160|Clcn3-RGD:2192|Hp-RGD:621252|Zmynd10-RGD:2192|Zmynd10-RGD:3889|Pdk1-RGD:727940|Pfkl-RGD:3889|Egln3-RGD:3889|Prepl-RGD:621862|Bcr-RGD:621725|Bcl6-RGD:3889|Vegfa-RGD:1306551|Vegfa-RGD:621252|Ell-RGD:3889|Kdr-Bnip3|Kdr-RGD:621252|Kdr-RGD:2192|Kdr-RGD:3889|Vldlr-RGD:1306551|Dnajb9-RGD:2192|Hk2-Bnip3l|Hk2-Bnip3|Hk2-RGD:628670|Hk2-RGD:2192|Hk2-RGD:727970|Eif2ak4-RGD:621252</td></tr>
</table>

<table>
<tr><th></th><th></th><th></th><th></th><th></th><th></th><th></th><th></th><th></th><th></th></tr>
<tr><td>GO biological process</td><td>GO:0048311</td><td>mitochondrion distribution</td><td>17</td><td>1.00E-04</td><td>0.0152</td><td>53</td><td>1</td><td>Dnm1l</td><td>Appl1-RGD:1307222|Appl1-RGD:1561168|Alkbh5-RGD:69400|Alkbh5-RGD:69436|Sec22b-RGD:69400|Sec22b-RGD:62060|Sec22b-RGD:1561168|Egln1-RGD:69436|Hikeshi-RGD:69436|Foxo4-RGD:62060|Pfkfb3-RGD:69436|Pfkfb3-RGD:1307222|Hspa1a-RGD:69329|Mllt11-RGD:69400|Eif4ebp1-RGD:69436|Acp5-Dnm1l|Tspo-RGD:621460|Dnm1l-RGD:628843|Dnm1l-RGD:69400|Dnm1l-RGD:708423|Dnm1l-RGD:69436|Dnm1l-RGD:1307222|Dnm1l-RGD:1561168|Bnip3-RGD:628843|Bnip3-RGD:621460|Bnip3-RGD:69329|Bnip3-RGD:1561168|Slc35e1-RGD:62060|Tomm7-RGD:628843|Tomm7-RGD:621460|Tomm7-RGD:1561168|Higd1a-RGD:621460|Bnip3l-RGD:69329|Nr4a3-Dnm1l|Nr4a3-RGD:3091|Nptx1-RGD:708423|Vopp1-RGD:62060|Scrn1-Dnm1l|Pdk1-RGD:62060|Noct-Dnm1l|Pfkl-RGD:62060|Egln3-Dnm1l|Egln3-RGD:1306110|Bcr-RGD:1561168|Kdr-RGD:1561168|Actn1-Dnm1l|Vldlr-Dnm1l|Hk2-RGD:628843|Hk2-Dnm1l|Hk2-RGD:69400|Hk2-RGD:621460|Hk2-RGD:62060|Hk2-RGD:69436</td></tr>
<tr><td>GO biological process</td><td>GO:0140052</td><td>cellular response to oxidised low-density lipoprotein particle stimulus</td><td>10</td><td>1.00E-04</td><td>0.01707</td><td>35</td><td>0</td><td></td><td>Appl1-RGD:2081|Il1rap-RGD:735043|Comt-RGD:735043|Acap1-RGD:2081|Foxo3-RGD:2081|Sec22b-RGD:735043|Egln1-RGD:2081|Hagh-RGD:2301|Foxo4-RGD:2081|Vwa1-RGD:2081|Faslg-RGD:2301|Hbp1-RGD:2081|Id3-RGD:2301|Hspa1a-RGD:2081|Hspa1a-RGD:3870|Mllt11-RGD:3870|Slc22a5-RGD:2081|Slc2a1-RGD:2301|Eif4ebp1-RGD:2081|Fcna-RGD:3870|Jmjd6-RGD:2301|Insig1-RGD:2081|Higd1a-RGD:2301|Slc27a1-RGD:2301|Nr4a3-RGD:2081|Clcn3-RGD:3870|Vopp1-RGD:2081|Prrc1-RGD:2081|Pfkl-RGD:735043|Bcr-RGD:2081|Ak4-RGD:2301|Vldlr-RGD:2301|Nol4-RGD:2081|Dusp1-RGD:2081|Dlc1-RGD:2081</td></tr>
<tr><td>GO biological process</td><td>GO:0001836</td><td>release of cytochrome c from mitochondria</td><td>22</td><td>4.00E-04</td><td>0.01574</td><td>71</td><td>1</td><td>Dnm1l</td><td>Sqle-RGD:2192|Bhlhe40-RGD:2943|Foxo3-RGD:3889|Foxo3-RGD:620103|Sec22b-RGD:628847|Sec22b-RGD:2200|Ero1a-RGD:2192|Pfkfb3-RGD:1306668|Ndrg1-RGD:2288|Dnajc3-RGD:620160|Mmp12-RGD:2179|Hspa1a-RGD:70984|Slc2a1-RGD:628847|Eif4ebp1-RGD:1306668|Eif4ebp1-RGD:3889|Kdm3a-RGD:3889|Acp5-Dnm1l|Tspo-RGD:620160|Tspo-RGD:2192|Tspo-RGD:620103|Dnm1l-RGD:70984|Dnm1l-RGD:1306668|Dnm1l-RGD:621635|Dnm1l-RGD:620160|Dnm1l-RGD:1310230|Dnm1l-RGD:2200|Bnip3-RGD:1306668|Bnip3-RGD:1310230|Bnip3-RGD:3889|Vsir-RGD:620103|Insig1-RGD:3889|Prkcq-RGD:70984|Prkcq-RGD:3889|Prkcq-RGD:2943|Tomm7-RGD:621635|Tomm7-RGD:620160|Higd1a-RGD:2192|Bnip3l-RGD:2200|Insig2-RGD:1306668|Adamts9-RGD:2199|Nr4a3-Dnm1l|Nr4a3-RGD:628847|Nr4a3-RGD:2943|Bcl11a-RGD:2199|Il33-RGD:620160|Clcn3-RGD:2192|Clcn3-RGD:2200|Scrn1-Dnm1l|Zmynd10-RGD:628847|Zmynd10-RGD:2192|Zmynd10-RGD:3889|Zmynd10-RGD:2199|Pdk1-RGD:3732|Noct-Dnm1l|Pfkl-RGD:3889|Egln3-Dnm1l|Egln3-RGD:3889|Egln3-RGD:2943|Atp11b-RGD:2179|Cited2-RGD:2943|Bcl6-RGD:3889|Ell-RGD:3889|Kdr-RGD:2192|Kdr-RGD:3889|Actn1-Dnm1l|Vldlr-Dnm1l|Dnajb9-RGD:70984|Dnajb9-RGD:2192|Hk2-Dnm1l|Hk2-RGD:2192|Eif2ak4-RGD:70984</td></tr>
</table>

GO biological process	GO:1903599	positive regulation of autophagy of mitochondrion	18	0	0.01707	63	3	Bnip3|Hk2|Tomm7	Bhlhe40-RGD:61928|Foxo3-RGD:61928|Egln1-RGD:61928|Egln1-RGD:68337|Pfkfb3-Hk2|Hbp1-RGD:61928|Mmp12-RGD:68337|Hspa1a-RGD:1305648|Gpi-Hk2|Mat2b-Hk2|Kdm3a-RGD:61928|Mmp9-RGD:68337|Tspo-Bnip3|Tspo-Hk2|Tspo-RGD:2181|Tspo-Tomm7|Dnm1l-RGD:628843|Dnm1l-RGD:71006|Dnm1l-RGD:1309944|Dnm1l-Hk2|Dnm1l-RGD:1305769|Bnip3-RGD:628843|Bnip3-RGD:61928|Bnip3-RGD:1309944|Bnip3-Hk2|Bnip3-RGD:1305769|Bnip3-Tomm7|Slc35e1-Hk2|Slc35e1-RGD:68337|Tomm7-RGD:628843|Tomm7-RGD:1589149|Tomm7-RGD:1594738|Tomm7-Bnip3|Tomm7-RGD:1309944|Tomm7-Hk2|Tomm7-RGD:1305769|Tomm7-RGD:2181|Bnip3l-Bnip3|Bnip3l-Hk2|Bnip3l-RGD:1305769|Bnip3l-Tomm7|Dnajb1-RGD:71006|Dnajb1-RGD:61928|Vopp1-RGD:68337|Scrn1-Hk2|Pdk1-RGD:61928|Prrc1-RGD:68337|Pfkl-RGD:61928|Pfkl-Hk2|Pfkl-RGD:68337|Egln3-RGD:61928|Vegfa-RGD:61928|Vegfa-RGD:68337|Kdr-Bnip3|Pgk1-RGD:61928|Dnajb9-RGD:61928|Hk2-RGD:628843|Hk2-Bnip3|Hk2-RGD:1305769|Hk2-RGD:68337|Hk2-Tomm7|Hexb-RGD:1589149|Ksr1-RGD:71006
GO biological process	GO:0016222	procollagen-proline 4-dioxygenase complex	4	0	0.02073	17	2	P4ha1|P4ha2	P4ha1-RGD:3244|P4ha1-P4ha2|P4ha2-P4ha1|P4ha2-RGD:735150|P4ha2-RGD:3244|Alkbh5-RGD:3244|Ero1a-RGD:3244|Egln1-RGD:735150|Egln1-RGD:3244|Egln1-P4ha2|Surf4-RGD:3244|Jmjd6-P4ha1|Egln3-P4ha1|Egln3-P4ha2|Plod2-P4ha1|Plod2-RGD:3244|Plod2-P4ha2
GO biological process	GO:0010940	positive regulation of necrotic cell death	13	0	0.01613	43	2	Tspo|Bnip3	Pygl-RGD:628899|Narf-Tspo|Foxo3-RGD:3889|Sec22b-RGD:628899|Sphk1-RGD:619956|Faslg-RGD:620018|Ndrg1-RGD:620018|Ndrg1-RGD:621872|Slc2a1-RGD:61912|Eif4ebp1-RGD:3889|Kdm3a-RGD:3889|Jmjd6-Tspo|Tspo-Bnip3|Tspo-RGD:621252|Dnm1l-RGD:621252|Dnm1l-RGD:621872|Bnip3-Tspo|Bnip3-RGD:3889|Map3k8-RGD:1310158|Insig1-RGD:3889|Prkcq-RGD:3889|Tomm7-Tspo|Tomm7-Bnip3|Bnip3l-Bnip3|Bnip3l-RGD:621252|Il33-RGD:628899|Clcn3-RGD:621872|Hp-RGD:621252|Parp16-RGD:628899|Zmynd10-RGD:3889|Pfkl-RGD:3889|Egln3-RGD:3889|Prepl-Tspo|Zap70-RGD:1310158|Bcl6-RGD:3889|Vegfa-RGD:621252|Ell-RGD:3889|Kdr-Bnip3|Kdr-RGD:621252|Kdr-RGD:3889|Hk2-Tspo|Hk2-Bnip3|Eif2ak4-RGD:621252
GO biological process	GO:0031545	peptidyl-proline 4-dioxygenase activity	7	0	0.01951	28	4	P4ha1|Egln3|P4ha2|Egln1	P4ha1-Egln3|P4ha1-RGD:3244|P4ha1-P4ha2|P4ha2-P4ha1|P4ha2-RGD:735150|P4ha2-Egln3|P4ha2-RGD:3244|P4ha2-RGD:631376|P4ha2-Egln1|Alkbh5-RGD:3244|Ero1a-RGD:3244|Egln1-RGD:735150|Egln1-Egln3|Egln1-RGD:3244|Egln1-RGD:631376|Egln1-P4ha2|Surf4-RGD:3244|Jmjd6-P4ha1|Jmjd6-Egln3|Dnm1l-Egln3|Dnm1l-RGD:631376|Egln3-P4ha1|Egln3-RGD:631376|Egln3-P4ha2|Egln3-Egln1|Plod2-P4ha1|Plod2-RGD:3244|Plod2-P4ha2

GO biological process	GO:1903146	regulation of autophagy of mitochondrion	38	0	0.01643	128	6	Bnip3l|Tspo|Dnm1l|Bnip3|Hk2|Tomm7	Appl1-RGD:620351|Narf-Tspo|Arrdc3-RGD:2321145|Comt-RGD:1307751|Comt-RGD:2321145|Comt-RGD:1306191|Bhlhe40-RGD:69423|Bhlhe40-RGD:61928|Foxo3-RGD:61928|Foxo3-RGD:3889|Egln1-RGD:61928|Egln1-RGD:68337|Pfkfb3-Hk2|Faslg-RGD:620351|Hbp1-RGD:61928|Mmp12-RGD:2321145|Mmp12-RGD:68337|Hspa1a-RGD:1305648|Mllt11-RGD:621575|Gpr1-RGD:619839|Slc2a1-RGD:2321145|Gpi-RGD:1307949|Gpi-Hk2|Mat2b-Hk2|Eif4ebp1-RGD:3889|Kdm3a-RGD:61928|Kdm3a-RGD:3889|Ccng2-RGD:620351|Fcna-RGD:2321145|Mmp9-RGD:68337|Jmjd6-Tspo|Acp5-Dnm1l|Tspo-RGD:621575|Tspo-Bnip3|Tspo-Hk2|Tspo-RGD:2181|Tspo-Tomm7|Dnm1l-RGD:628843|Dnm1l-RGD:71006|Dnm1l-RGD:1309944|Dnm1l-RGD:621575|Dnm1l-Hk2|Dnm1l-RGD:1305769|Bnip3-RGD:628843|Bnip3-Bnip3l|Bnip3-RGD:1309944|Bnip3-Tspo|Bnip3-RGD:61928|Bnip3-Hk2|Bnip3-RGD:1305769|Bnip3-RGD:3889|Bnip3-Tomm7|Slc35e1-Hk2|Slc35e1-RGD:68337|Insig1-RGD:3889|Prkcq-RGD:620351|Prkcq-RGD:1306882|Prkcq-RGD:3889|Tomm7-RGD:628843|Tomm7-Bnip3l|Tomm7-RGD:1309944|Tomm7-RGD:1560038|Tomm7-Tspo|Tomm7-RGD:1589149|Tomm7-RGD:1560364|Tomm7-RGD:1594738|Tomm7-Bnip3|Tomm7-Hk2|Tomm7-RGD:1305769|Tomm7-RGD:2181|Bnip3l-RGD:620351|Bnip3l-RGD:621575|Bnip3l-Bnip3|Bnip3l-Hk2|Bnip3l-RGD:1305769|Bnip3l-Tomm7|Dnajb1-RGD:71006|Dnajb1-RGD:61928|Nr4a3-RGD:69423|Nr4a3-RGD:620351|Nr4a3-Dnm1l|Bcl11a-RGD:1307751|Il33-RGD:620351|Il33-RGD:2321145|Clcn3-RGD:621575|Vopp1-RGD:68337|Scrn1-Dnm1l|Scrn1-Hk2|Parp16-RGD:620351|Parp16-RGD:621575|Zmynd10-RGD:3889|Pdk1-RGD:61928|Noct-Dnm1l|Prrc1-RGD:68337|Pfkl-RGD:61928|Pfkl-Hk2|Pfkl-RGD:3889|Pfkl-RGD:68337|Egln3-Dnm1l|Egln3-RGD:61928|Egln3-RGD:3889|Prepl-Tspo|Prepl-RGD:621575|Bcl6-RGD:3889|Vegfa-RGD:61928|Vegfa-RGD:68337|Ell-RGD:3889|Kdr-RGD:1307949|Kdr-Bnip3|Kdr-RGD:3889|Actn1-Dnm1l|Pgk1-RGD:61928|Vldlr-Dnm1l|Dnajb9-RGD:61928|Hk2-
GO biological process	GO:0045429	positive regulation of nitric oxide biosynthetic process	48	9.00E-04	0.01626	160	0		Appl1-RGD:2082|Appl1-RGD:2081|Plpp1-RGD:68371|Pygl-RGD:68371|Narf-RGD:628841|Arrdc3-RGD:628841|Arrdc3-RGD:9292348|Arrdc3-RGD:2866|Il1r2-RGD:2901|Il1r2-RGD:68371|Comt-RGD:628841|Acap1-RGD:2081|Bhlhe40-RGD:3032|Foxo3-RGD:3032|Foxo3-RGD:2081|Cd200-RGD:2513|Alkbh5-RGD:3234|Sec22b-RGD:628841|Sec22b-RGD:628847|Sec22b-RGD:61924|Sec22b-RGD:631409|Egln1-RGD:1559992|Egln1-RGD:631409|Egln1-RGD:2081|Hagh-RGD:2301|Foxo4-RGD:2581|Foxo4-RGD:3032|Foxo4-RGD:2081|Tspan31-RGD:68371|Vwa1-RGD:2581|Vwa1-RGD:2081|Faslg-RGD:2301|Faslg-RGD:2857|Faslg-RGD:3876|Ndrg1-RGD:2411|Ndrg1-RGD:620349|Ndrg1-RGD:3876|Hbp1-RGD:2581|Hbp1-RGD:2081|Mmp12-RGD:631408|Id3-RGD:2301|Hspa1a-RGD:3907|Hspa1a-RGD:2081|Hspa1a-RGD:3870|Mllt11-RGD:2857|Mllt11-RGD:2866|Mllt11-RGD:3870|Slc22a5-RGD:2082|Slc22a5-RGD:2081|Cblb-RGD:2830|Cblb-RGD:2139|Slc2a1-RGD:2301|Slc2a1-RGD:628847|Slc2a1-RGD:628758|Slc2a1-RGD:620349|Slc2a1-RGD:2082|Gpi-RGD:2901|Gpi-RGD:3876|Lrrc8a-RGD:68371|Eif4ebp1-RGD:2081|Eif4ebp1-RGD:68371|Kdm3a-RGD:3032|Efna1-RGD:2139|Efna1-RGD:68371|Fcna-RGD:3870|Mmp9-RGD:3032|Mmp9-RGD:3907|Mmp9-RGD:631408|Jmjd6-RGD:2301|Acp5-RGD:9292348|Dnm1l-RGD:2513|Dnm1l-RGD:2139|Dnm1l-RGD:9292348|Bnip3-RGD:628841|Bnip3-RGD:2139|Slc35e1-RGD:61924|Slc35e1-RGD:2917|Slc35e1-RGD:2082|Vsir-RGD:3032|Acvrl1-RGD:631409|Acvrl1-RGD:68371|Map3k8-RGD:631409|Insig1-RGD:2081|Prkcq-RGD:621445|Prkcq-RGD:3876|Higd1a-RGD:2301|Bnip3l-RGD:628841|Slc27a1-RGD:2301|Slc27a1-RGD:620349|Slc27a1-RGD:3876|Klc4-RGD:2163|Klc4-RGD:2082|Nr4a3-RGD:628847|Nr4a3-RGD:2082|Nr4a3-RGD:2081|Nr4a3-RGD:68371|Bcl11a-RGD:2139|Clcn3-RGD:2513|Clcn3-RGD:3234|Clcn3-RGD:3907|Clcn3-RGD:2857|Clcn3-RGD:631409|Clcn3-RGD:2163|Clcn3-RGD:3870|Nptx1-RGD:3234|Wnt4-RGD:2891|Wnt4-RGD:2901|Wnt4-RGD:2139|Wnt4-RGD:1559992|Wnt4-RGD:2069|Vopp1-RGD:2581|Vopp1-RGD:61924|Vopp1-

<table>
<tr><th></th><th></th><th></th><th></th><th></th><th></th><th></th><th></th><th></th><th></th></tr>
<tr><td>GO biological process</td><td>GO:0035794</td><td>positive regulation of mitochondrial membrane permeability</td><td>17</td><td>0</td><td>0.01578</td><td>55</td><td>2</td><td>Bnip3l|Bnip3</td><td>Sqle-RGD:2192|Gpt-RGD:1306551|Foxo3-RGD:3889|Sec22b-RGD:1306551|Ero1a-RGD:2192|Vwa1-RGD:3718|Ndrg1-RGD:628774|Dnajc3-RGD:620160|Dnajc3-RGD:3718|Cblb-RGD:1306551|Eif4ebp1-RGD:3889|Kdm3a-RGD:3889|Tspo-RGD:620160|Tspo-Bnip3|Tspo-RGD:628774|Tspo-RGD:2192|Dnm1l-RGD:620160|Dnm1l-RGD:628774|Dnm1l-RGD:727970|Bnip3-Bnip3l|Bnip3-RGD:1307023|Bnip3-RGD:3889|Insig1-RGD:3889|Prkcq-RGD:3889|Prkcq-RGD:621862|Tomm7-Bnip3l|Tomm7-RGD:1307023|Tomm7-RGD:620160|Tomm7-Bnip3|Higd1a-RGD:628670|Higd1a-RGD:2192|Higd1a-RGD:621862|Higd1a-RGD:727970|Bnip3l-Bnip3|Il33-RGD:620160|Clcn3-RGD:2192|Zmynd10-RGD:2192|Zmynd10-RGD:3889|Pdk1-RGD:727940|Pfkl-RGD:3889|Egln3-RGD:3889|Prepl-RGD:621862|Bcl6-RGD:3889|Vegfa-RGD:1306551|Ell-RGD:3889|Kdr-Bnip3|Kdr-RGD:2192|Kdr-RGD:3889|Vldlr-RGD:1306551|Dnajb9-RGD:2192|Hk2-Bnip3l|Hk2-Bnip3|Hk2-RGD:628670|Hk2-RGD:2192|Hk2-RGD:727970</td></tr>
<tr><td>GO biological process</td><td>GO:0140212</td><td>regulation of long-chain fatty acid import into cell</td><td>8</td><td>0.001</td><td>0.02439</td><td>40</td><td>0</td><td></td><td>Appl1-RGD:69316|Appl1-RGD:2082|Appl1-RGD:2081|Slc27a3-RGD:69403|Gpt-RGD:69403|Acap1-RGD:2081|Foxo3-RGD:2081|Egln1-RGD:2081|Foxo4-RGD:2081|Vwa1-RGD:2081|Hbp1-RGD:2081|Id3-RGD:69048|Hspa1a-RGD:69403|Hspa1a-RGD:2081|Slc22a5-RGD:2082|Slc22a5-RGD:2081|Slc2a1-RGD:2082|Eif4ebp1-RGD:2081|Rasd1-RGD:69048|Slc35e1-RGD:2082|Insig1-RGD:2081|Slc27a1-RGD:69403|Slc27a1-RGD:69402|Klc4-RGD:2082|Nr4a3-RGD:2082|Nr4a3-RGD:2081|Wnt4-RGD:69048|Vopp1-RGD:2081|Pdk1-RGD:69402|Prrc1-RGD:2082|Prrc1-RGD:69403|Prrc1-RGD:2081|Bcr-RGD:2081|Vegfa-RGD:1588455|Vegfa-RGD:69403|Kdr-RGD:2082|Nol4-RGD:2081|Dusp1-RGD:2081|Dlc1-RGD:2081|Hk2-RGD:2082</td></tr>
<tr><td>GO biological process</td><td>GO:1905709</td><td>negative regulation of membrane permeability</td><td>9</td><td>5.00E-04</td><td>0.01788</td><td>33</td><td>2</td><td>Hk2|Bnip3</td><td>Hikeshi-RGD:620261|Pfkfb3-Hk2|Gpi-Hk2|Mat2b-Hk2|Tspo-Hk2|Tspo-RGD:620352|Tspo-Bnip3|Dnm1l-Hk2|Dnm1l-RGD:620352|Dnm1l-RGD:708558|Bnip3-Hk2|Bnip3-RGD:620261|Bnip3-RGD:620482|Slc35e1-Hk2|Tomm7-Hk2|Tomm7-RGD:708558|Tomm7-Bnip3|Higd1a-RGD:620352|Higd1a-RGD:708558|Bnip3l-Hk2|Bnip3l-RGD:620352|Bnip3l-Bnip3|Slc27a1-RGD:708558|Scrn1-Hk2|Pdk1-RGD:708558|Pfkl-Hk2|Prepl-RGD:708558|Kdr-Bnip3|Vldlr-RGD:708558|Hk2-RGD:620352|Hk2-RGD:708558|Hk2-RGD:620482|Hk2-Bnip3</td></tr>
<tr><td>GO biological process</td><td>GO:0001893</td><td>maternal placenta development</td><td>12</td><td>1.00E-04</td><td>0.01585</td><td>39</td><td>0</td><td></td><td>Appl1-RGD:2081|Acap1-RGD:2081|Bhlhe40-RGD:69305|Bhlhe40-RGD:3611|Bhlhe40-RGD:3610|Foxo3-RGD:2081|Egln1-RGD:2081|Hagh-RGD:2301|Foxo4-RGD:2081|Vwa1-RGD:2081|Faslg-RGD:2301|Hbp1-RGD:2081|Id3-RGD:2301|Id3-RGD:69305|Id3-RGD:3610|Hspa1a-RGD:2081|Slc22a5-RGD:2081|Slc2a1-RGD:2301|Eif4ebp1-RGD:2081|Kdm3a-RGD:3611|Efna1-RGD:71082|Jmjd6-RGD:2301|Dnm1l-RGD:3611|Insig1-RGD:2081|Higd1a-RGD:2301|Slc27a1-RGD:2301|Nr4a3-RGD:2081|Bcl11a-RGD:69305|Vopp1-RGD:2081|Prrc1-RGD:2081|Bcr-RGD:2081|Ak4-RGD:2301|Vegfa-RGD:71082|Cdkn1b-RGD:69305|Vldlr-RGD:2301|Nol4-RGD:2081|Dusp1-RGD:2081|Dlc1-RGD:2081|Eif2ak4-RGD:71082</td></tr>
</table>

GO biological process	GO:1903715	regulation of aerobic respiration	8	0	0.0189	31	1	Bnip3	Bhlhe40-RGD:61928|Foxo3-RGD:61928|Ero1a-RGD:621595|Egln1-RGD:61928|Pfkfb3-RGD:1310547|Hbp1-RGD:61928|Hspa1a-RGD:2861|Kdm3a-RGD:61928|Tspo-Bnip3|Bnip3-RGD:61928|Insig1-RGD:621595|Tomm7-Bnip3|Bnip3l-Bnip3|Dnajb1-RGD:61928|Insig2-RGD:621595|Klc4-RGD:2861|Zmynd10-RGD:621595|Pdk1-RGD:1310547|Pdk1-RGD:61928|Prrc1-RGD:2861|Pfkl-RGD:61928|Pfkl-RGD:621595|Egln3-RGD:61928|Vegfa-RGD:61928|Kdr-Bnip3|Actn1-RGD:621792|Pgk1-RGD:61928|Pgk1-RGD:621595|Dnajb9-RGD:61928|Dnajb9-RGD:621595|Hk2-Bnip3
GO biological process	GO:0072655	establishment of protein localization to mitochondrion	48	0	0.01524	150	2	Tspo|Hk2	Sqle-RGD:2192|Appl1-RGD:2081|Plpp1-RGD:2257|Narf-Tspo|Arrdc3-RGD:2259|Arrdc3-RGD:3980|Il1r2-RGD:2257|Slc27a3-RGD:628878|Acap1-RGD:2081|Foxo3-RGD:2081|Cd200-RGD:2257|Alkbh5-RGD:3980|Sec22b-RGD:628847|Sec22b-RGD:620817|Sec22b-RGD:2258|Sec22b-RGD:631409|Sec22b-RGD:631441|Ero1a-RGD:2192|Egln1-RGD:2259|Egln1-RGD:631409|Egln1-RGD:2081|Sphk1-RGD:1562046|Sphk1-RGD:2258|Sphk1-RGD:2257|Hikeshi-RGD:2259|Foxo4-RGD:2081|Vwa1-RGD:2081|Pfkfb3-RGD:2259|Pfkfb3-RGD:1306668|Pfkfb3-Hk2|Faslg-RGD:1598163|Faslg-RGD:1303260|Faslg-RGD:1307279|Faslg-RGD:1303022|Faslg-RGD:631441|Faslg-RGD:1303049|Faslg-RGD:1562440|Faslg-RGD:1303219|Ndrg1-RGD:3980|Ndrg1-RGD:621801|Ndrg1-RGD:621656|Ndrg1-RGD:621741|Dnajc3-RGD:620160|Dnajc3-RGD:628878|Scpep1-RGD:2259|Hbp1-RGD:2081|Id3-RGD:62391|Hspa1a-RGD:1305648|Hspa1a-RGD:2081|Gpr1-RGD:2259|Slc22a5-RGD:2257|Slc22a5-RGD:2081|Slc2a1-RGD:628847|Slc2a1-RGD:2319280|Slc2a1-RGD:1307279|Slc2a1-RGD:1303022|Gpi-Hk2|Mat2b-Hk2|Eif4ebp1-RGD:1306668|Eif4ebp1-RGD:2081|Ramp3-RGD:628619|Rasd1-RGD:1562440|Efna1-RGD:2257|Fcna-RGD:2257|Jmjd6-Tspo|Tspo-RGD:620160|Tspo-RGD:2192|Tspo-Hk2|Dnm1l-RGD:628843|Dnm1l-RGD:620160|Dnm1l-RGD:2259|Dnm1l-RGD:2258|Dnm1l-RGD:2257|Dnm1l-RGD:1310230|Dnm1l-RGD:62391|Dnm1l-RGD:1306668|Dnm1l-RGD:1308080|Dnm1l-RGD:628878|Dnm1l-Hk2|Dnm1l-RGD:1305769|Dnm1l-RGD:7706142|Bnip3-RGD:628843|Bnip3-RGD:1309005|Bnip3-RGD:1310230|Bnip3-RGD:708467|Bnip3-Tspo|Bnip3-RGD:1306668|Bnip3-RGD:1308080|Bnip3-Hk2|Bnip3-RGD:1305769|Slc35e1-Hk2|Acvrl1-RGD:631409|Map3k8-RGD:631409|Insig1-RGD:2081|Prkcq-RGD:628619|Tomm7-RGD:628843|Tomm7-RGD:620160|Tomm7-RGD:708467|Tomm7-Tspo|Tomm7-Hk2|Tomm7-RGD:1305769|Tomm7-RGD:1303219|Higd1a-RGD:2192|Bnip3l-RGD:1308080|Bnip3l-Hk2|Bnip3l-RGD:1305769|Stbd1-RGD:2257|Slc16a3-
GO biological process	GO:1905395	response to flavonoid	8	0.001	0.01829	30	1	Dnm1l	Plpp1-RGD:2756|Pfkfb3-RGD:1306668|Slc22a5-RGD:2756|Eif4ebp1-RGD:1306668|Acp5-Dnm1l|Tspo-RGD:621460|Dnm1l-RGD:628843|Dnm1l-RGD:1306668|Dnm1l-RGD:2756|Entpd2-RGD:2756|Bnip3-RGD:628843|Bnip3-RGD:1306668|Bnip3-RGD:621460|Tomm7-RGD:628843|Tomm7-RGD:621460|Higd1a-RGD:621460|Slc16a3-RGD:2756|Insig2-RGD:1306668|Slc27a1-RGD:2756|Nr4a3-Dnm1l|Clcn3-RGD:2756|Scrn1-Dnm1l|Noct-Dnm1l|Egln3-Dnm1l|Actn1-Dnm1l|Vldlr-Dnm1l|Hk2-RGD:628843|Hk2-Dnm1l|Hk2-RGD:621460|Hk2-RGD:2756
GO biological process	GO:0051541	elastin metabolic process	6	6.00E-04	0.01544	19	1	Mmp12	Bhlhe40-RGD:61928|Foxo3-RGD:61928|Egln1-RGD:61928|Ndrg1-RGD:3000|Hbp1-RGD:61928|Mmp12-RGD:2179|Kdm3a-RGD:61928|Mmp9-Mmp12|Acp5-RGD:3000|Bnip3-RGD:61928|Dnajb1-RGD:61928|Il33-RGD:2448|Pdk1-RGD:61928|Pfkl-RGD:61928|Egln3-RGD:61928|Atp11b-RGD:2179|Vegfa-RGD:61928|Pgk1-RGD:61928|Dnajb9-RGD:61928

GO biological process	GO:0002248	connective tissue replacement involved in inflammatory response wound healing	5	0	0.02341	24	0		Narf-RGD:2890|Il1r2-RGD:2890|Bhlhe40-RGD:61928|Foxo3-RGD:61928|Egln1-RGD:61928|Sphk1-RGD:2586|Faslg-RGD:2890|Ndrg1-RGD:621675|Dnajc3-RGD:2890|Hbp1-RGD:61928|Kdm3a-RGD:61928|Mmp9-RGD:621675|Acp5-RGD:2890|Bnip3-RGD:61928|Dnajb1-RGD:61928|Slc27a1-RGD:2586|Wnt4-RGD:2890|Parp16-RGD:2890|Pdk1-RGD:61928|Pfkl-RGD:61928|Egln3-RGD:61928|Vegfa-RGD:61928|Pgk1-RGD:61928|Dnajb9-RGD:61928
GO biological process	GO:0097411	hypoxia-inducible factor-1alpha signaling pathway	4	1.00E-04	0.01829	15	1	Pdk1	Bhlhe40-RGD:61928|Foxo3-RGD:61928|Egln1-RGD:61928|Hbp1-RGD:61928|Kdm3a-RGD:61928|Bnip3-RGD:61928|Dnajb1-RGD:61928|Pdk1-RGD:1593276|Pdk1-RGD:61928|Pdk1-RGD:620574|Pfkl-RGD:61928|Egln3-RGD:61928|Vegfa-RGD:61928|Pgk1-RGD:61928|Dnajb9-RGD:61928
GO biological process	GO:1990910	response to hypobaric hypoxia	8	0	0.0189	31	1	Dnm1l	Pfkfb3-RGD:1306668|Ndrg1-RGD:620402|Hspa1a-RGD:620913|Gpr1-RGD:620402|Eif4ebp1-RGD:1306668|Acp5-Dnm1l|Tspo-RGD:621460|Dnm1l-RGD:628843|Dnm1l-RGD:1306668|Dnm1l-RGD:620402|Bnip3-RGD:628843|Bnip3-RGD:1306668|Bnip3-RGD:621460|Tomm7-RGD:628843|Tomm7-RGD:621460|Higd1a-RGD:621460|Dnajb1-RGD:620913|Insig2-RGD:1306668|Nr4a3-Dnm1l|Scrn1-Dnm1l|Zmynd10-RGD:621539|Noct-Dnm1l|Egln3-Dnm1l|Bcl6-RGD:620913|Vegfa-RGD:620402|Plau-RGD:620402|Actn1-Dnm1l|Vldlr-Dnm1l|Hk2-RGD:628843|Hk2-Dnm1l|Hk2-RGD:621460
GO biological process	GO:0097237	cellular response to toxic substance	13	2.00E-04	0.01538	41	0		Narf-RGD:61845|Bhlhe40-RGD:61928|Foxo3-RGD:61928|Sec22b-RGD:61845|Sec22b-RGD:62060|Sec22b-RGD:632280|Egln1-RGD:61928|Foxo4-RGD:62060|Pfkfb3-RGD:1306668|Faslg-RGD:3876|Ndrg1-RGD:3876|Hbp1-RGD:61928|Gpi-RGD:3876|Eif4ebp1-RGD:1306668|Guca1a-RGD:628829|Kdm3a-RGD:61928|Dnm1l-RGD:1305769|Dnm1l-RGD:1306668|Bnip3-RGD:1305769|Bnip3-RGD:1306668|Bnip3-RGD:61928|Slc35e1-RGD:62060|Prkcq-RGD:632280|Prkcq-RGD:3876|Tomm7-RGD:1305769|Bnip3l-RGD:1305769|Dnajb1-RGD:61928|Insig2-RGD:1306668|Slc27a1-RGD:3876|Clcn3-RGD:628829|Vopp1-RGD:62060|Pdk1-RGD:62060|Pdk1-RGD:61928|Pfkl-RGD:62060|Pfkl-RGD:61928|Egln3-RGD:61928|Vegfa-RGD:61928|Pgk1-RGD:61928|Dnajb9-RGD:61928|Hk2-RGD:1305769|Hk2-RGD:62060
GO biological process	GO:0006007	glucose catabolic process	11	1.00E-04	0.01507	34	1	Hk2	Foxo3-RGD:1309643|Foxo3-RGD:620103|Sec22b-RGD:2553|Pfkfb3-Hk2|Pfkfb3-RGD:68419|Slc22a5-RGD:6491529|Gpi-Hk2|Gpi-RGD:2554|Gpi-RGD:2553|Mat2b-Hk2|Tspo-Hk2|Tspo-RGD:620103|Dnm1l-Hk2|Bnip3-Hk2|Slc35e1-Hk2|Vsir-RGD:620103|Tomm7-Hk2|Bnip3l-Hk2|Slc27a1-RGD:2554|Il33-RGD:2553|Clcn3-RGD:2554|Scrn1-Hk2|Parp16-RGD:2553|Pfkl-Hk2|Pfkl-RGD:68419|Pfkl-RGD:6491529|Egln3-RGD:1309643|Vegfa-RGD:1309329|Kdr-RGD:6491529|Pgk1-RGD:2554|Aldoc-RGD:6491529|Hk2-RGD:2554|Hk2-RGD:68419|Hk2-RGD:3309
GO biological process	GO:0031543	peptidyl-proline dioxygenase activity	9	0	0.01734	32	4	P4ha1|Egln3|P4ha2|Egln1	P4ha1-RGD:1308848|P4ha1-RGD:1304568|P4ha1-Egln3|P4ha1-RGD:3244|P4ha1-P4ha2|P4ha2-P4ha1|P4ha2-RGD:1304568|P4ha2-RGD:735150|P4ha2-Egln3|P4ha2-RGD:3244|P4ha2-RGD:631376|P4ha2-Egln1|Alkbh5-RGD:3244|Ero1a-RGD:3244|Egln1-RGD:735150|Egln1-Egln3|Egln1-RGD:3244|Egln1-RGD:631376|Egln1-P4ha2|Surf4-RGD:3244|Jmjd6-RGD:1308848|Jmjd6-P4ha1|Jmjd6-Egln3|Dnm1l-Egln3|Dnm1l-RGD:631376|Egln3-P4ha1|Egln3-RGD:631376|Egln3-P4ha2|Egln3-Egln1|Plod2-P4ha1|Plod2-RGD:3244|Plod2-P4ha2

GO biological process	GO:1902110	positive regulation of mitochondrial membrane permeability involved in apoptotic process	11	3.00E-04	0.01552	35	1	Bnip3	Sqle-RGD:2192|Gpt-RGD:1306551|Sec22b-RGD:1306551|Ero1a-RGD:2192|Ndrg1-RGD:628774|Dnajc3-RGD:620160|Cblb-RGD:1306551|Tspo-RGD:628774|Tspo-RGD:2192|Tspo-RGD:620160|Tspo-Bnip3|Dnm1l-RGD:628774|Dnm1l-RGD:727970|Dnm1l-RGD:620160|Bnip3-RGD:1307023|Prkcq-RGD:621862|Tomm7-RGD:1307023|Tomm7-RGD:620160|Tomm7-Bnip3|Higd1a-RGD:2192|Higd1a-RGD:621862|Higd1a-RGD:727970|Bnip3l-Bnip3|Il33-RGD:620160|Clcn3-RGD:2192|Zmynd10-RGD:2192|Prepl-RGD:621862|Vegfa-RGD:1306551|Kdr-RGD:2192|Kdr-Bnip3|Vldlr-RGD:1306551|Dnajb9-RGD:2192|Hk2-RGD:2192|Hk2-RGD:727970|Hk2-Bnip3
GO biological process	GO:0061030	epithelial cell differentiation involved in mammary gland alveolus development	4	3.00E-04	0.01951	16	0		Bhlhe40-RGD:2859|Bhlhe40-RGD:61928|Foxo3-RGD:61928|Egln1-RGD:61928|Hbp1-RGD:61928|Id3-RGD:2859|Kdm3a-RGD:61928|Bnip3-RGD:61928|Dnajb1-RGD:61928|Nr4a3-RGD:2859|Pdk1-RGD:61928|Pfkl-RGD:61928|Egln3-RGD:61928|Vegfa-RGD:61928|Pgk1-RGD:61928|Dnajb9-RGD:61928
GO biological process	GO:0035655	interleukin-18-mediated signaling pathway	5	1.00E-04	0.02731	28	0		Appl1-RGD:2081|Il1rap-RGD:1308589|Acap1-RGD:2081|Foxo3-RGD:2081|Alkbh5-RGD:3283|Egln1-RGD:2081|Foxo4-RGD:2081|Vwa1-RGD:2081|Hbp1-RGD:2081|Hspa1a-RGD:2081|Mllt11-RGD:2889|Slc22a5-RGD:2081|Eif4ebp1-RGD:2081|Acvrl1-RGD:3283|Insig1-RGD:2081|Slc27a1-RGD:2889|Nr4a3-RGD:2081|Clcn3-RGD:2889|Wnt4-RGD:2889|Vopp1-RGD:2081|Prrc1-RGD:2081|Prepl-RGD:2889|Bcr-RGD:2081|Bcl6-RGD:2889|Kdr-RGD:3283|Nol4-RGD:2081|Dusp1-RGD:2081|Dlc1-RGD:2081
GO biological process	GO:0035795	negative regulation of mitochondrial membrane permeability	7	2.00E-04	0.02299	33	2	Hk2|Bnip3	Hikeshi-RGD:620261|Pfkfb3-Hk2|Gpi-Hk2|Mat2b-Hk2|Tspo-Hk2|Tspo-RGD:620352|Tspo-Bnip3|Dnm1l-Hk2|Dnm1l-RGD:620352|Dnm1l-RGD:708558|Bnip3-Hk2|Bnip3-RGD:620261|Bnip3-RGD:620482|Slc35e1-Hk2|Tomm7-Hk2|Tomm7-RGD:708558|Tomm7-Bnip3|Higd1a-RGD:620352|Higd1a-RGD:708558|Bnip3l-Hk2|Bnip3l-RGD:620352|Bnip3l-Bnip3|Slc27a1-RGD:708558|Scrn1-Hk2|Pdk1-RGD:708558|Pfkl-Hk2|Prepl-RGD:708558|Kdr-Bnip3|Vldlr-RGD:708558|Hk2-RGD:620352|Hk2-RGD:708558|Hk2-RGD:620482|Hk2-Bnip3

GO biological process	GO:1903428	positive regulation of reactive oxygen species biosynthetic process	64	1.00E-04	0.01501	197	2	Clcn3|Foxo3	P4ha1-RGD:620573|Appl1-RGD:2082|Appl1-RGD:2081|Appl1-RGD:2736|Plpp1-RGD:68371|Pygl-RGD:68371|Narf-RGD:628841|Arrdc3-RGD:628841|Arrdc3-RGD:9292348|Arrdc3-RGD:2866|Il1r2-RGD:2901|Il1r2-RGD:68371|Comt-RGD:628841|Acap1-RGD:2081|Bhlhe40-RGD:3032|Foxo3-RGD:2081|Foxo3-RGD:3032|Cd200-RGD:2513|Alkbh5-RGD:3234|Sec22b-RGD:628841|Sec22b-RGD:628847|Sec22b-RGD:631409|Sec22b-RGD:62060|Sec22b-RGD:619979|Sec22b-RGD:61924|Sec22b-RGD:621808|Egln1-RGD:631409|Egln1-RGD:2081|Egln1-RGD:1559992|Hagh-RGD:2301|Hagh-RGD:621808|Foxo4-RGD:2581|Foxo4-Foxo3|Foxo4-RGD:2081|Foxo4-RGD:62060|Foxo4-RGD:3032|Tspan31-RGD:68371|Vwa1-RGD:2581|Vwa1-RGD:2081|Faslg-RGD:2857|Faslg-RGD:2301|Faslg-RGD:621808|Faslg-RGD:3876|Ndrg1-RGD:2898|Ndrg1-RGD:621872|Ndrg1-RGD:2411|Ndrg1-RGD:620349|Ndrg1-RGD:3876|Dnajc3-RGD:620573|Hbp1-RGD:2581|Hbp1-RGD:2081|Mmp12-RGD:631408|Id3-RGD:2301|Hspa1a-RGD:3907|Hspa1a-RGD:2081|Hspa1a-RGD:3870|Mllt11-RGD:2857|Mllt11-RGD:2866|Mllt11-RGD:3870|Gpr1-Clcn3|Slc22a5-RGD:620573|Slc22a5-RGD:2082|Slc22a5-RGD:2081|Cblb-RGD:2139|Cblb-RGD:2830|Slc2a1-RGD:628847|Slc2a1-RGD:2082|Slc2a1-RGD:2301|Slc2a1-RGD:628758|Slc2a1-RGD:620349|Gpi-RGD:2901|Gpi-RGD:3876|Lrrc8a-RGD:68371|Eif4ebp1-RGD:2081|Eif4ebp1-RGD:68371|Kdm3a-RGD:3032|Efna1-RGD:2139|Efna1-RGD:68371|Fcna-RGD:3870|Mmp9-RGD:3907|Mmp9-RGD:631408|Mmp9-RGD:3032|Jmjd6-RGD:2301|Acp5-RGD:9292348|Acp5-RGD:620573|Dnm1l-RGD:628843|Dnm1l-RGD:2513|Dnm1l-RGD:2139|Dnm1l-RGD:621872|Dnm1l-RGD:9292348|Dnm1l-RGD:620918|Bnip3-RGD:628843|Bnip3-RGD:628841|Bnip3-RGD:2139|Slc35e1-RGD:2082|Slc35e1-RGD:62060|Slc35e1-RGD:61924|Slc35e1-RGD:2917|Vsir-RGD:3032|Acvrl1-RGD:631409|Acvrl1-RGD:68371|Map3k8-RGD:631409|Insig1-RGD:2081|Prkcq-RGD:621445|Prkcq-RGD:3876|Tomm7-RGD:628843|Higd1a-RGD:2301|Bnip3l-RGD:628841|Basp1-RGD:621808|Slc27a1-RGD:2301|Slc27a1-
GO biological process	GO:0032909	regulation of transforming growth factor beta2 production	8	0	0.01829	30	0		Bhlhe40-RGD:3032|Bhlhe40-RGD:2666|Bhlhe40-RGD:61928|Foxo3-RGD:3032|Foxo3-RGD:3033|Foxo3-RGD:61928|Egln1-RGD:61928|Foxo4-RGD:3032|Foxo4-RGD:3033|Hbp1-RGD:61928|Kdm3a-RGD:3032|Kdm3a-RGD:61928|Mmp9-RGD:3032|Bnip3-RGD:61928|Vsir-RGD:3032|Prkcq-RGD:621862|Higd1a-RGD:621862|Dnajb1-RGD:61928|Fbln1-RGD:3032|Fbln1-RGD:3033|Pdk1-RGD:61928|Pfkl-RGD:61928|Egln3-RGD:2666|Egln3-RGD:61928|Prepl-RGD:621862|Cited2-RGD:3032|Vegfa-RGD:61928|Plau-RGD:70491|Pgk1-RGD:61928|Dnajb9-RGD:61928
GO biological process	GO:0006848	pyruvate transport	4	2.00E-04	0.01829	15	0		Bhlhe40-RGD:3130|Foxo3-RGD:3130|Hbp1-RGD:3130|Slc22a5-RGD:3691|Slc2a1-RGD:3691|Gpi-RGD:1563422|Eif4ebp1-RGD:3130|Slc16a3-RGD:3130|Slc27a1-RGD:620902|Nr4a3-RGD:3130|Mnt-RGD:3130|Pdk1-RGD:3130|Pdk1-RGD:620902|Pdk1-RGD:1563422|Plau-RGD:3130

<table>
<tr><td>GO biological process</td><td>GO:0048312</td><td>intracellular distribution of mitochondria</td><td>12</td><td>0</td><td>0.02113</td><td>52</td><td>1</td><td>Dnm1l</td><td>Appl1-RGD:1561168|Appl1-RGD:1307222|Alkbh5-RGD:69400|Alkbh5-RGD:69436|Sec22b-RGD:69400|Sec22b-RGD:1561168|Sec22b-RGD:62060|Egln1-RGD:69436|Hikeshi-RGD:69436|Foxo4-RGD:62060|Pfkfb3-RGD:69436|Pfkfb3-RGD:1307222|Hspa1a-RGD:69329|Mllt11-RGD:69400|Eif4ebp1-RGD:69436|Acp5-Dnm1l|Tspo-RGD:621460|Dnm1l-RGD:628843|Dnm1l-RGD:69400|Dnm1l-RGD:708423|Dnm1l-RGD:1561168|Dnm1l-RGD:69436|Dnm1l-RGD:1307222|Bnip3-RGD:628843|Bnip3-RGD:621460|Bnip3-RGD:69329|Bnip3-RGD:1561168|Slc35e1-RGD:62060|Tomm7-RGD:628843|Tomm7-RGD:621460|Tomm7-RGD:1561168|Higd1a-RGD:621460|Bnip3l-RGD:69329|Nr4a3-Dnm1l|Nr4a3-RGD:3091|Nptx1-RGD:708423|Vopp1-RGD:62060|Scrn1-Dnm1l|Pdk1-RGD:62060|Noct-Dnm1l|Pfkl-RGD:62060|Egln3-Dnm1l|Bcr-RGD:1561168|Kdr-RGD:1561168|Actn1-Dnm1l|Vldlr-Dnm1l|Hk2-RGD:628843|Hk2-Dnm1l|Hk2-RGD:69400|Hk2-RGD:621460|Hk2-RGD:62060|Hk2-RGD:69436</td></tr>
<tr><td>GO biological process</td><td>GO:0097709</td><td>connective tissue replacement</td><td>6</td><td>1.00E-04</td><td>0.02113</td><td>26</td><td>0</td><td></td><td>Narf-RGD:3940|Narf-RGD:2890|Il1r2-RGD:2890|Bhlhe40-RGD:61928|Foxo3-RGD:61928|Egln1-RGD:61928|Sphk1-RGD:2586|Faslg-RGD:2890|Ndrg1-RGD:621675|Dnajc3-RGD:2890|Hbp1-RGD:61928|Mllt11-RGD:3940|Kdm3a-RGD:61928|Mmp9-RGD:621675|Acp5-RGD:2890|Bnip3-RGD:61928|Dnajb1-RGD:61928|Slc27a1-RGD:2586|Wnt4-RGD:2890|Parp16-RGD:2890|Pdk1-RGD:61928|Pfkl-RGD:61928|Egln3-RGD:61928|Vegfa-RGD:61928|Pgk1-RGD:61928|Dnajb9-RGD:61928</td></tr>
<tr><td>GO biological process</td><td>GO:0070815</td><td>peptidyl-lysine 5-dioxygenase activity</td><td>4</td><td>0</td><td>0.01951</td><td>16</td><td>2</td><td>Jmjd6|Plod2</td><td>P4ha1-RGD:621382|P4ha1-Jmjd6|P4ha1-Plod2|P4ha1-RGD:631339|P4ha2-RGD:621382|P4ha2-Plod2|P4ha2-RGD:631339|Alkbh5-Jmjd6|Jmjd6-RGD:621382|Jmjd6-RGD:631339|Tspo-Jmjd6|Egln3-RGD:621382|Egln3-Jmjd6|Egln3-RGD:631339|Plod2-RGD:631339|Hk2-RGD:621382</td></tr>
<tr><td>GO biological process</td><td>GO:1902109</td><td>negative regulation of mitochondrial membrane permeability involved in apoptotic process</td><td>4</td><td>1.00E-04</td><td>0.02439</td><td>20</td><td>1</td><td>Bnip3</td><td>Tspo-RGD:620352|Tspo-Bnip3|Dnm1l-RGD:620352|Dnm1l-RGD:708558|Bnip3-RGD:620482|Tomm7-RGD:708558|Tomm7-Bnip3|Higd1a-RGD:620352|Higd1a-RGD:708558|Bnip3l-RGD:620352|Bnip3l-Bnip3|Slc27a1-RGD:708558|Pdk1-RGD:708558|Prepl-RGD:708558|Kdr-Bnip3|Vldlr-RGD:708558|Hk2-RGD:620352|Hk2-RGD:708558|Hk2-RGD:620482|Hk2-Bnip3</td></tr>
</table>

GO biological process	GO:0071276	cellular response to cadmium ion	32	1.00E-04	0.01722	113	1	Mmp9	Sqle-RGD:70500|Appl1-RGD:70500|Appl1-RGD:2543|Appl1-RGD:2081|Narf-RGD:61307|Gpt-RGD:70500|Acap1-RGD:2081|Bhlhe40-RGD:2943|Foxo3-RGD:70500|Foxo3-RGD:2543|Foxo3-RGD:2081|Sec22b-RGD:628847|Sec22b-RGD:621506|Sec22b-RGD:621227|Sec22b-RGD:68415|Egln1-RGD:2081|Hikeshi-RGD:1306919|Hagh-RGD:1303089|Foxo4-RGD:2081|Vwa1-RGD:2806|Vwa1-RGD:2081|Faslg-RGD:3770|Faslg-RGD:68415|Ndrg1-Mmp9|Ndrg1-RGD:2459|Dnajc3-RGD:1306919|Hbp1-RGD:708428|Hbp1-RGD:2081|Mmp12-RGD:2179|Mmp12-Mmp9|Hspa1a-RGD:3731|Hspa1a-RGD:61307|Hspa1a-RGD:620913|Hspa1a-RGD:2081|Mllt11-RGD:619912|Slc22a5-RGD:2081|Cblb-RGD:61307|Slc2a1-RGD:628847|Slc2a1-RGD:70500|Slc2a1-RGD:621506|Slc2a1-RGD:2806|Gpi-RGD:68415|Eif4ebp1-RGD:2081|Kdm3a-RGD:621227|Rasd1-RGD:621506|Mmp9-RGD:2806|Tspo-RGD:621252|Dnm1l-RGD:621252|Dnm1l-RGD:2543|Lrif1-RGD:61307|Slc35e1-RGD:2806|Hltf-RGD:621725|Map3k8-RGD:1306661|Insig1-RGD:1303089|Insig1-RGD:2081|Prkcq-RGD:1306661|Prkcq-RGD:70500|Prkcq-RGD:2943|Prkcq-RGD:619912|Bnip3l-RGD:621252|Dnajb1-RGD:620913|Insig2-RGD:3731|Slc27a1-RGD:3770|Klc4-RGD:3731|Klc4-RGD:61307|Nr4a3-RGD:628847|Nr4a3-RGD:2943|Nr4a3-RGD:2806|Nr4a3-RGD:2081|Il33-RGD:3770|Il33-RGD:619912|Hp-RGD:621252|Vopp1-RGD:2806|Vopp1-RGD:61307|Vopp1-RGD:2081|Scrn1-RGD:61307|Zmynd10-RGD:3770|Zmynd10-RGD:628847|Pdk1-RGD:620574|Noct-RGD:61307|Gzf1-RGD:61307|Prrc1-RGD:3731|Prrc1-RGD:2806|Prrc1-RGD:2081|Pfkl-RGD:2543|Egln3-RGD:2943|Egln3-RGD:68415|Prepl-RGD:619912|Bcr-RGD:61307|Bcr-RGD:621725|Bcr-RGD:2081|Atp11b-RGD:2179|Zap70-RGD:61307|Cited2-RGD:2943|Bcl6-RGD:2806|Bcl6-RGD:620913|Tbl2-RGD:621506|Vegfa-RGD:621252|Kdr-RGD:70500|Kdr-RGD:621252|Kdr-RGD:1303089|Vldlr-RGD:621227|Dnajb9-RGD:68415|Nol4-RGD:2081|Dusp1-RGD:708428|Dusp1-RGD:3046|Dusp1-RGD:2081|Dlc1-RGD:2081|Hk2-RGD:2543|Eif2ak4-RGD:70500|Eif2ak4-RGD:621506|Eif2ak4-RGD:621252|Ksr1-
GO biological process	GO:0071380	cellular response to prostaglandin E stimulus	18	1.00E-04	0.01653	61	0		Appl1-RGD:3387|Appl1-RGD:2081|Narf-RGD:2716|Acap1-RGD:2081|Bhlhe40-RGD:3371|Foxo3-RGD:2081|Cd200-RGD:628641|Sec22b-RGD:3387|Sec22b-RGD:2716|Sec22b-RGD:3371|Egln1-RGD:2081|Foxo4-RGD:2081|Vwa1-RGD:2081|Pfkfb3-RGD:620893|Ndrg1-RGD:2624|Ndrg1-RGD:621074|Ndrg1-RGD:619816|Hbp1-RGD:2081|Id3-RGD:620832|Id3-RGD:3371|Hspa1a-RGD:2081|Slc22a5-RGD:61925|Slc22a5-RGD:2081|Slc2a1-RGD:620832|Slc2a1-RGD:3387|Slc2a1-RGD:620893|Eif4ebp1-RGD:2081|Gng2-RGD:2716|Acvrl1-RGD:628641|Insig1-RGD:2081|Prkcq-RGD:619816|Prkcq-RGD:620893|Dnajb1-RGD:2624|Stbd1-RGD:3371|Nr4a3-RGD:3387|Nr4a3-RGD:2081|Clcn3-RGD:61798|Clcn3-RGD:620269|Vopp1-RGD:61925|Vopp1-RGD:2081|Parp16-RGD:2716|Prrc1-RGD:2081|Prkag2-RGD:3387|Prkag2-RGD:620893|Pfkl-RGD:621248|Prepl-RGD:620893|Bcr-RGD:2081|Bcl6-RGD:61925|Bcl6-RGD:619816|Vegfa-RGD:2624|Vegfa-RGD:621241|Kdr-RGD:2624|Kdr-RGD:620893|Pgk1-RGD:2624|Nol4-RGD:2081|Dusp1-RGD:2081|Dlc1-RGD:2081|Eif2ak4-RGD:3387|Eif2ak4-RGD:620893|Ksr1-RGD:3387|Ksr1-RGD:620893
GO biological process	GO:0004656	procollagen-proline 4-dioxygenase activity	4	0	0.02073	17	2	P4ha1|P4ha2	P4ha1-RGD:3244|P4ha1-P4ha2|P4ha2-P4ha1|P4ha2-RGD:735150|P4ha2-RGD:3244|Alkbh5-RGD:3244|Ero1a-RGD:3244|Egln1-RGD:735150|Egln1-RGD:3244|Egln1-P4ha2|Surf4-RGD:3244|Jmjd6-P4ha1|Egln3-P4ha1|Egln3-P4ha2|Plod2-P4ha1|Plod2-RGD:3244|Plod2-P4ha2

GO biological process	GO:1903377	negative regulation of oxidative stress-induced neuron intrinsic apoptotic signaling pathway	5	0.001	0.02536	26	0		Narf-RGD:1549738|Bhlhe40-RGD:61928|Foxo3-RGD:61928|Sec22b-RGD:621808|Egln1-RGD:61928|Hagh-RGD:621808|Faslg-RGD:621808|Hbp1-RGD:61928|Kdm3a-RGD:61928|Dnm1l-RGD:1305769|Bnip3-RGD:1305769|Bnip3-RGD:61928|Tomm7-RGD:1305769|Bnip3l-RGD:1305769|Dnajb1-RGD:61928|Basp1-RGD:621808|Ifi27-RGD:621808|Nr4a3-RGD:621808|Pdk1-RGD:61928|Pfkl-RGD:61928|Egln3-RGD:61928|Vegfa-RGD:61928|Pgk1-RGD:621808|Pgk1-RGD:61928|Dnajb9-RGD:61928|Hk2-RGD:1305769
GO biological process	GO:1903147	negative regulation of autophagy of mitochondrion	8	4.00E-04	0.01646	27	1	Tspo	Narf-Tspo|Foxo3-RGD:3889|Gpi-RGD:1307949|Eif4ebp1-RGD:3889|Kdm3a-RGD:3889|Jmjd6-Tspo|Dnm1l-RGD:1305769|Bnip3-Tspo|Bnip3-RGD:1305769|Bnip3-RGD:3889|Insig1-RGD:3889|Prkcq-RGD:3889|Tomm7-Tspo|Tomm7-RGD:1305769|Tomm7-RGD:1560038|Bnip3l-RGD:1305769|Zmynd10-RGD:3889|Pfkl-RGD:3889|Egln3-RGD:3889|Prepl-Tspo|Bcl6-RGD:3889|Ell-RGD:3889|Kdr-RGD:3889|Kdr-RGD:1307949|Hk2-Tspo|Hk2-RGD:1305769|Hk2-RGD:1560038
GO biological process	GO:0072656	maintenance of protein location in mitochondrion	5	0	0.04	41	2	Tspo|Hk2	Appl1-RGD:2081|Narf-Tspo|Acap1-RGD:2081|Foxo3-RGD:2081|Egln1-RGD:2081|Foxo4-RGD:2081|Vwa1-RGD:2081|Pfkfb3-Hk2|Hbp1-RGD:2081|Hspa1a-RGD:2081|Slc22a5-RGD:2081|Gpi-Hk2|Mat2b-Hk2|Eif4ebp1-RGD:2081|Jmjd6-Tspo|Tspo-Hk2|Dnm1l-Hk2|Dnm1l-RGD:1305769|Bnip3-Tspo|Bnip3-Hk2|Bnip3-RGD:1305769|Slc35e1-Hk2|Insig1-RGD:2081|Tomm7-Tspo|Tomm7-Hk2|Tomm7-RGD:1305769|Bnip3l-Hk2|Bnip3l-RGD:1305769|Nr4a3-RGD:2081|Vopp1-RGD:2081|Scrn1-Hk2|Prrc1-RGD:2081|Pfkl-Hk2|Prepl-Tspo|Bcr-RGD:2081|Nol4-RGD:2081|Dusp1-RGD:2081|Dlc1-RGD:2081|Hk2-Tspo|Hk2-RGD:1305769|Hk2-RGD:2796
GO biological process	GO:0045838	positive regulation of membrane potential	25	0	0.01756	90	0		Appl1-RGD:2082|Appl1-RGD:2081|Appl1-RGD:70982|Narf-RGD:621531|Acap1-RGD:2081|Bhlhe40-RGD:3130|Foxo3-RGD:3130|Foxo3-RGD:620103|Foxo3-RGD:2081|Alkbh5-RGD:2518|Sec22b-RGD:70951|Sec22b-RGD:70982|Ero1a-RGD:621595|Egln1-RGD:2081|Foxo4-RGD:2081|Vwa1-RGD:2081|Dnajc3-RGD:620160|Scpep1-RGD:1308285|Hbp1-RGD:3130|Hbp1-RGD:2081|Hspa1a-RGD:621531|Hspa1a-RGD:3708|Hspa1a-RGD:2081|Mllt11-RGD:3708|Slc22a5-RGD:2082|Slc22a5-RGD:2081|Slc2a1-RGD:2082|Mat2b-RGD:621531|Eif4ebp1-RGD:3130|Eif4ebp1-RGD:2081|Tspo-RGD:621460|Tspo-RGD:620160|Tspo-RGD:620103|Dnm1l-RGD:628843|Dnm1l-RGD:1308285|Dnm1l-RGD:620160|Dnm1l-RGD:70982|Bnip3-RGD:628843|Bnip3-RGD:621460|Lrif1-RGD:621531|Slc35e1-RGD:2082|Vsir-RGD:620103|Insig1-RGD:628871|Insig1-RGD:2081|Insig1-RGD:70982|Insig1-RGD:621595|Prkcq-RGD:70982|Tomm7-RGD:628843|Tomm7-RGD:621460|Tomm7-RGD:620160|Higd1a-RGD:621460|Slc16a3-RGD:3130|Insig2-RGD:1308285|Insig2-RGD:70982|Insig2-RGD:621595|Klc4-RGD:3708|Klc4-RGD:2082|Nr4a3-RGD:708413|Nr4a3-RGD:3130|Nr4a3-RGD:2082|Nr4a3-RGD:2081|Nr4a3-RGD:70982|Mnt-RGD:3130|Il33-RGD:620160|Hp-RGD:1587346|Vopp1-RGD:2081|Scrn1-RGD:621531|Scrn1-RGD:3708|Zmynd10-RGD:70951|Zmynd10-RGD:621595|Pdk1-RGD:3130|Noct-RGD:3708|Prrc1-RGD:2082|Prrc1-RGD:2081|Prrc1-RGD:70982|Pfkl-RGD:621595|Egln3-RGD:70982|Bcr-RGD:2081|Kdr-RGD:2082|Plau-RGD:3130|Actn1-RGD:620157|Pgk1-RGD:621595|Dnajb9-RGD:621595|Nol4-RGD:2081|Dusp1-RGD:2081|Dlc1-RGD:2081|Hk2-RGD:628843|Hk2-RGD:621460|Hk2-RGD:2082|Hk2-RGD:70982

GO biological process	GO:0031264	death-inducing signaling complex	6	7.00E-04	0.01544	19	0	Arrdc3-RGD:2275|Alkbh5-RGD:619831|Sec22b-RGD:620945|Sec22b-RGD:2275|Hikeshi-RGD:619831|Faslg-RGD:620847|Faslg-RGD:619831|Faslg-RGD:620945|Faslg-RGD:2275|Cblb-RGD:2275|Efna1-RGD:620945|Efna1-RGD:2275|Casp6-RGD:620945|Casp6-RGD:2275|Map3k8-RGD:1310158|Wnt4-RGD:619831|Zap70-RGD:1310158|Cdkn1b-RGD:2275|Eif2ak4-RGD:628700
GO biological process	GO:0010918	positive regulation of mitochondrial membrane potential	17	0	0.02123	74	0	Appl1-RGD:2082|Appl1-RGD:2081|Appl1-RGD:70982|Acap1-RGD:2081|Bhlhe40-RGD:3130|Foxo3-RGD:3130|Foxo3-RGD:620103|Foxo3-RGD:2081|Sec22b-RGD:70982|Ero1a-RGD:621595|Egln1-RGD:2081|Foxo4-RGD:2081|Vwa1-RGD:2081|Dnajc3-RGD:620160|Scpep1-RGD:1308285|Hbp1-RGD:3130|Hbp1-RGD:2081|Hspa1a-RGD:2081|Slc22a5-RGD:2082|Slc22a5-RGD:2081|Slc2a1-RGD:2082|Eif4ebp1-RGD:3130|Eif4ebp1-RGD:2081|Tspo-RGD:621460|Tspo-RGD:620160|Tspo-RGD:620103|Dnm1l-RGD:628843|Dnm1l-RGD:1308285|Dnm1l-RGD:620160|Dnm1l-RGD:70982|Bnip3-RGD:628843|Bnip3-RGD:621460|Slc35e1-RGD:2082|Vsir-RGD:620103|Insig1-RGD:2081|Insig1-RGD:621595|Insig1-RGD:70982|Prkcq-RGD:70982|Tomm7-RGD:628843|Tomm7-RGD:621460|Tomm7-RGD:620160|Higd1a-RGD:621460|Slc16a3-RGD:3130|Insig2-RGD:1308285|Insig2-RGD:621595|Insig2-RGD:70982|Klc4-RGD:2082|Nr4a3-RGD:3130|Nr4a3-RGD:2082|Nr4a3-RGD:2081|Nr4a3-RGD:70982|Mnt-RGD:3130|Il33-RGD:620160|Hp-RGD:1587346|Vopp1-RGD:2081|Zmynd10-RGD:621595|Pdk1-RGD:3130|Prrc1-RGD:2082|Prrc1-RGD:2081|Prrc1-RGD:70982|Pfkl-RGD:621595|Egln3-RGD:70982|Bcr-RGD:2081|Kdr-RGD:2082|Plau-RGD:3130|Pgk1-RGD:621595|Dnajb9-RGD:621595|Nol4-RGD:2081|Dusp1-RGD:2081|Dlc1-RGD:2081|Hk2-RGD:628843|Hk2-RGD:621460|Hk2-RGD:2082|Hk2-RGD:70982
