# Supplementary figures and images for: Predicted rat interactome database and gene set linkage analysis
Source: Database (Oxford). 2020 Nov 20;2020:baaa086. doi: 10.1093/database/baaa086 (PMC7678787; doi:10.1093/database/baaa086)

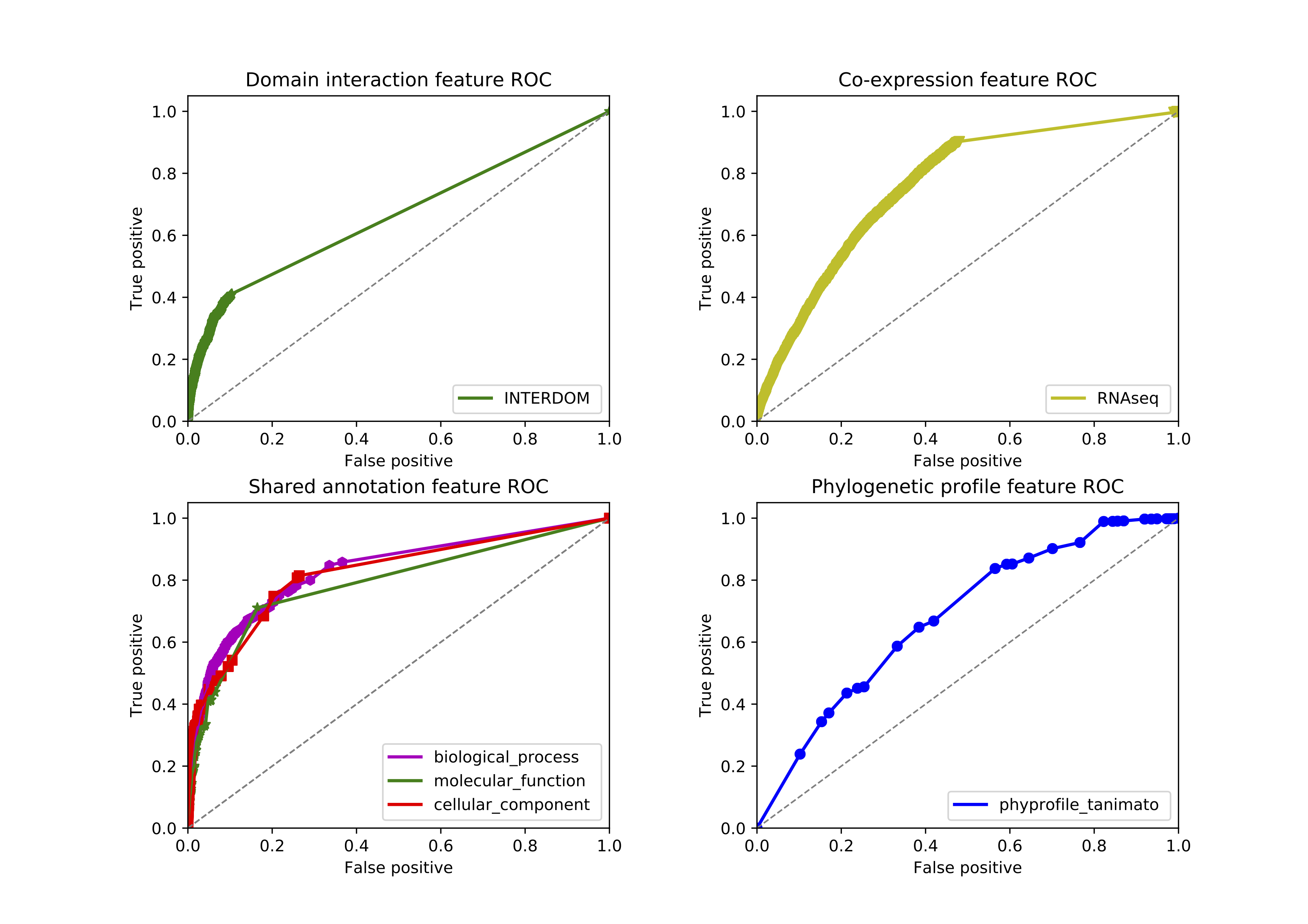

Supplement: baaa086_Supp [file baaa086_supp.zip › Supplementary_Figure_s1_-_rat.png]
